# Plasmon-Induced Hot-State Multiexciton Emission from Quantum Dots Coupled to Metallic Nanocavities

**DOI:** 10.1021/acsnano.6c01353

**Published:** 2026-05-26

**Authors:** Yonatan Ossia, Nadav Chefetz, Adar Levi, Einav Scharf, Oren Goldberg, Sergei Remennik, Atzmon Vakahi, Uriel Levy, Uri Banin

**Affiliations:** † Institute of Chemistry, 98519 The Hebrew University of Jerusalem, Jerusalem 91904, Israel; ‡ Institute of Applied Physics, The Hebrew University of Jerusalem, Jerusalem 91904, Israel; § The Center for Nanoscience and Nanotechnology, The Hebrew University of Jerusalem, Jerusalem 91904, Israel

**Keywords:** nanophotonics, plasmonics, colloidal quantum dots, multiexciton emission, aluminum nanocavities, single-particle spectroscopy, auger suppression

## Abstract

Nonradiative Auger quenching strongly limits multi-exciton (MX) emission in semiconductor quantum dots (QDs), restricting their performance in high-power photonic and electro-optical applications. We report efficient hot-state MX emission by coupling colloidal QDs into plasmonic aluminum nanohole cavities, manifesting significantly enhanced photoluminescence from the 1P_e_–1P_3/2_ transition and higher hot MX states, with emission energies up to 500 meV above the band-edge transition. Power-dependent photoluminescence shows up to 15-fold increase of the hot MX emission compared with pristine QDs on glass, at 10-fold lower nominal MX density. At relatively low excitation powers corresponding to an average exciton population of ∼0.3, a significant photoluminescence blue shift is observed, assigned to efficient emission from multiply charged excitons, indicating cavity-induced photo charging of the QD. Investigating the influence of nanohole size, QD location within the cavity, and power and wavelength-dependent excitation establishes the central role of plasmon-induced energy transfer and hot charge injection from the excited metal cavity to the coupled QD, which, alongside plasmonic enhancement of QD absorption and emission, yields the efficient hot MX emission. This mechanism is supported by numerical simulations and comparing metallic cavities differing by their plasmonic spectral response, providing direct evidence for the importance of state filling, through plasmon-driven metal–QD interactions, for hot MX emission. This distinctive ability to activate otherwise quenched MX states allows broadband and tunable emission from individual QDs embedded in nanosized plasmonic cavities, offering a controlled chip-scale approach for color tuning. These findings promote pathways for high-power applications in photocatalysis, tunable microlasers, broadband light sources, and correlated multiphoton sources for quantum technologies.

## Graphical abstract

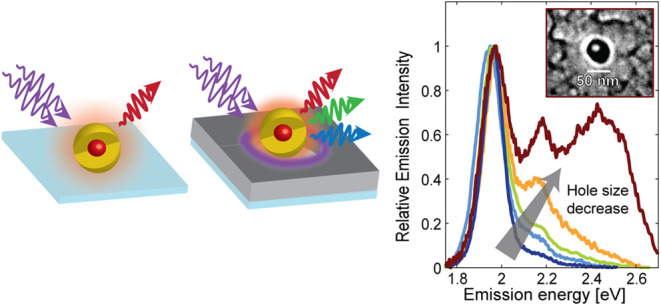

## Introduction

Semiconductor quantum dots (QDs), commonly used in modern electro-optical applications, enhance the electronic properties of bulk semiconductors via size-dependent optical tunability governed by the effect of quantum confinement, creating discretized atom-like excitonic energy states.^1,2^ While the full spectrum of these energy states is revealed in the QD absorption profile, QD photoluminescence (PL) essentially arises from exciton recombination of the lowest-energy 1S_e_–1S_3/2_ conduction band to valence-band (CB-VB) transition, leading to narrow band emission. This is due to the ultrafast relaxation processes of the high-energy excited CB and VB states to the band-edge at (sub)­picosecond time scales,^3–5^ much faster than typical radiative recombination. At higher excitation powers, biexcitons may form, and when more than two excitons are excited in a QD, higher-energy states such as the 1P_e_–1P_3/2_ band transition are populated with multiple hot-excitons (MX), which can radiatively recombine to emit photons of significantly higher energy than the band-edge single and biexciton (1X, BX, respectively) states.^6,7^ However, QD emission intensity tends to saturate with increasing excitation power due to an efficient many-body Auger recombination,^8^ a nonradiative process that is significantly enhanced for small QDs, with a process rate typically orders of magnitude faster than its radiative rate (*k*_MX,NR_ ≫ *k*_MX,R_), resulting in low quantum yield (QY) of MX emission.^9^

Overcoming this nonradiative quenching of MX emission is key to unlocking QDs’ full potential for high-power photonic applications such as QD-based lasing and electroluminescence^10,11^ and correlated multiphoton quantum light sources,^12,13^ and enhancing efficiency in photocatalysis^14,15^ and photovoltaics.^16^ Several strategies have been explored to address the low QY of high-energy MX emission. These include heterostructure engineering of QDs with reduced Auger recombination,^17^ such as seeded nanorods,^18^ quantum shells,^19^ or employing large-volume QDs with semi-bulk character^20^ that reduce carrier wave function overlap and the Auger rates while enhancing MX emission,^7,21^ though nonradiative losses remain dominant. Alternatively, charging QD films by adding electrons to the CB through electrochemical bias or chemical doping lowers the average exciton population in a single QD, ⟨*N*⟩, required for the population inversion of band-edge lasing,^22^ and can facilitate MX emission from the 1P_e_–1P_3/2_ band, while typically quenching overall emission and reducing the 1X QY.^23,24^

An alternative promising approach for scalable emission enhancement has been the coupling of QDs to optical and plasmonic nano-cavities. In such systems, subwavelength electromagnetic confinement modifies the free-space optical properties of both the cavity and QD,^25,26^ giving rise to distinctive optoelectronic behavior governed by the coupling strength, defined as the coherent energy exchange rate between a quantum emitter and an electromagnetic mode, with field enhancement provided by cavity or plasmonic effects. Under weak coupling, QDs exhibit altered photoemission where plasmonic cavities act as nanoantennas, altering the local density of optical states (LDOS). This leads to Purcell-effect-driven changes in emission rates, fluorescence enhancement or quenching,^27–29^ and even modified far-field polarization.^30^ In the strong coupling regime, exciton–plasmon interactions mix light and matter into hybrid polaritons, evidenced by Rabi splitting and hybridized states in single QDs coupled to bowtie^31^ and nanoparticle-on-mirror nanocavities.^32^ Importantly, if cavity coupling enhances QD radiative rates sufficiently to compete with Auger recombination and band-edge relaxation, higher-energy multiexciton (MX) states can also emit. In PbS QDs, coupling to nanoparticle-on-mirror cavities showed greatly shortened PL lifetimes and a Stokes shift reduction, linked to enabling band-edge radiative recombination before relaxation to intraband trap states.^33^ Furthermore, coupled metal-QD nanostructures provide a platform for studying exciton–dipole interactions,^34,35^ inducing energy^36^ and charge transfer.^37,38^ Energy and charge transfer can proceed through the coherent plasmon oscillations of nonthermal electron–hole pairs generated at the metal–semiconductor interface,^39^ or through tunneling of nonequilibrium energetic electrons from excited metal-CB states into the adjacent semiconductor CB, when the coupling is strong enough to overcome the Schottky barrier.^40^ These hot charge (HCT) and energy transfer (ET) pathways boost the free carrier population in the semiconductor beyond its intrinsic absorption, and have become a central focus in current research due to their relevance in plasmonic photodetectors,^41,42^ solar energy harvesting, and photocatalysis.^43–45^ Metal–semiconductor HCT has previously been shown in colloidal hybrid-metal–semiconductor nanocrystals, such as gold-tipped CdSe nanorods,^38,46^ and in Au-TiO_2_ nanocomposites, where HCT exhibits a size-dependent response to the metal domain, arising from a combination of direct and indirect plasmon-induced pathways along with available interband transitions in the metal CB.^47^ Metal–semiconductor ET has been explored from a theoretical perspective^48,49^ and in metal–insulator–semiconductor photocatalysis,^36^ where the barrier between the metal and semiconductor reduces the yield of HCT.

While HCT and ET have not yet been demonstrated to directly modify the emissive behavior of semiconductor QDs, a comparable mechanism was reported between gold nanoparticles on MoS_2_ monolayers, where electron doping of the MoS_2_ drove a lattice phase transition that yielded a red shift in the band-edge PL and strengthened higher-energy emissive transitions.^50^ Aluminum (Al) supports the same plasmonic and interband HCT/ET mechanisms as the commonly used gold, exhibiting plasmonic functionality that extends into the blue and ultraviolet regions of the spectrum, and manifesting higher resistance to oxidation than silver.^43^ Aluminum nanocrystals have been shown to be capable of injecting charge into coupled semiconductors for enhanced photocatalysis,^51,52^ even across their 2–4 nm native oxide barrier. Recent atomistic models indicate that the energy distribution of hot electrons and holes in aluminum nanoparticles is nearly uniform up to the excitation energy, enabling both hot electron and hole transfer.^53^ However, electron transfer across the ∼4 nm Al_2_O_3_ layer is expected to be more efficient due to the lower potential barrier between the aluminum Fermi level and the CdS conduction band, as well as the smaller effective mass of electrons. In addition, the aluminum Fermi level lies closer to the CdS conduction band minimum than that of gold,^43^ promoting more efficient electron transfer.

Herein, we report on achieving efficient MX PL from above band-edge states in single CdSe/CdS core–shell QDs coupled to Al-nanohole plasmonic cavities. Emission from the 1P_e_–1P_3/2_ transition and higher MX states appears up to 500 meV (≈130 nm blue shift) above the 1S_e_–1S_3/2_ single-exciton band-edge transition, at average exciton population: ⟨*N*⟩, over ten times lower than pristine QDs on glass and previous ambient studies. We combine both excitation power and wavelength-dependent measurements of MX PL in nanoholes of varying size and metal composition with scanning electron microscopy (SEM) imaging of the in-hole QD location, as well as numerical Finite-difference time-domain (FDTD) simulations. Together, these results point to a dual mechanism combining a moderate plasmon-enhanced QD absorption/emission, with an efficient QD state filling by plasmon-induced HCT and ET from the excited metal cavities to adjacent QDs, driving MX enhancement at both low and high excitation powers.

## Results and Discussion

### Enhanced MX Emission in QDs Coupled to Al Nanohole Cavities

Metallic nanohole cavities with varying diameters were fabricated in 30 nm thick metal films evaporated on glass microscope slides, using either an electron beam lithography (EBL) and lift-off process, or focused ion beam milling (FIB) of the nanohole patterns in the film (additional details are in methods). Highly emissive CdSe/CdS core–shell QDs, with an average overall diameter of 16 nm (2.2/5.5 nm core radius/shell thickness), were synthesized and characterized following well-established procedures.^54^ A dilute solution of QDs was drop-cast onto microscope slides containing nanohole arrays spaced 4 μm apart to minimize interactions between neighboring holes. Optical characterization of individual QD-nanohole systems was performed on a custom-built inverted microscope setup, described in previous works.^55,56^ Following optical characterization, nanohole dimensions and QD positions within the cavities were verified by SEM imaging of the very same cavity. High-resolution STEM (scanning transmission electron microscopy) cross sections combined with energy dispersive spectroscopy (EDS) elemental mapping on selected nanoholes were also done, verifying QD composition and height within the nanohole, using our previously reported on-chip correlation method (supporting Figures S1,S2).^55,56^

Figure 1(a) presents a schematic illustration of exciton state filling in a pristine semiconductor QD, and (b) a QD coupled to a plasmonic cavity. Figure 1c presents the PL spectra of a single QD on a glass substrate under varying excitation powers of a 405 nm pulsed picosecond laser. The primary emission peak, centered at 1.920 ± 0.005 eV, corresponds to the 1X transition in the 1S_e_–1S_3/2_ band. The increase in excitation power raises the likelihood of generating multiple excitons in the QD, quantified by the Poissonian-average number of laser-induced excitons ⟨N⟩ per pulse, calculated from the PL saturation fit explained in detail later (Supporting Figure S3 and Note S1 ). The main emission peak shows a slight blue shift and eventually saturates at ⟨*N*⟩ = 8.6 ± 0.5 (41fJ per pulse), originating from the contribution of additional MX states (biexcitons, triexcitons, and possibly charged MX species). Despite the high ⟨*N*⟩, where excitons or charges occupy higher-energy states, the QD emission is dominated by 1S_e_–1S_3/2_ band-edge recombination. At very high excitation powers corresponding formally to ⟨*N*⟩= 66 ± 5 (315 fJ), a faint secondary peak emerges at 2.10 ± 0.02 eV, attributed to a transition involving the 1P_e_–1P_3/2_ band exciton.^57^ Figure 1d shows the results of a similar experiment for a single QD embedded in a 110 nm diameter nanohole within a 30 nm thick Aluminum film on glass. In this case, a blue-shifted 1S_e_–1S_3/2_ transition is already evident at low excitation powers. Moreover, a secondary peak corresponding to the 1P_e_–1P_3/2_ transition already emerges at moderate power excitation corresponding to ⟨*N*⟩ = 3.7 ± 0.6 (32 fJ), which is about 20 times lower than the exciton population threshold observed on the glass substrate (where the QD absorption cross section is nearly double due to less light entering the nanohole, Supporting Figure S3). The intensity of the 1P_e_–1P_3/2_ peak increases with rising ⟨*N*⟩, accompanied by the appearance of additional higher-energy transitions, extending up to 2.45 ± 0.05 eV, as constrained by a 460 nm long-pass filter in the detection path. To quantify high-band MX emission from energy levels above the band edge, we compare the integrated areas of Gaussian-fitted PL peaks corresponding in energy to the 1P_e_–1P_3/2_ and higher band-transitions, with those within the 1S_e_–1S_3/2_ band, providing a measure of the relative high-energy MX emission $\frac{{\mathrm{PL}}_{highMX}}{{\mathrm{PL}}_{1S_{e}-1S_{3/2}}}$ .

**Figure 1:**
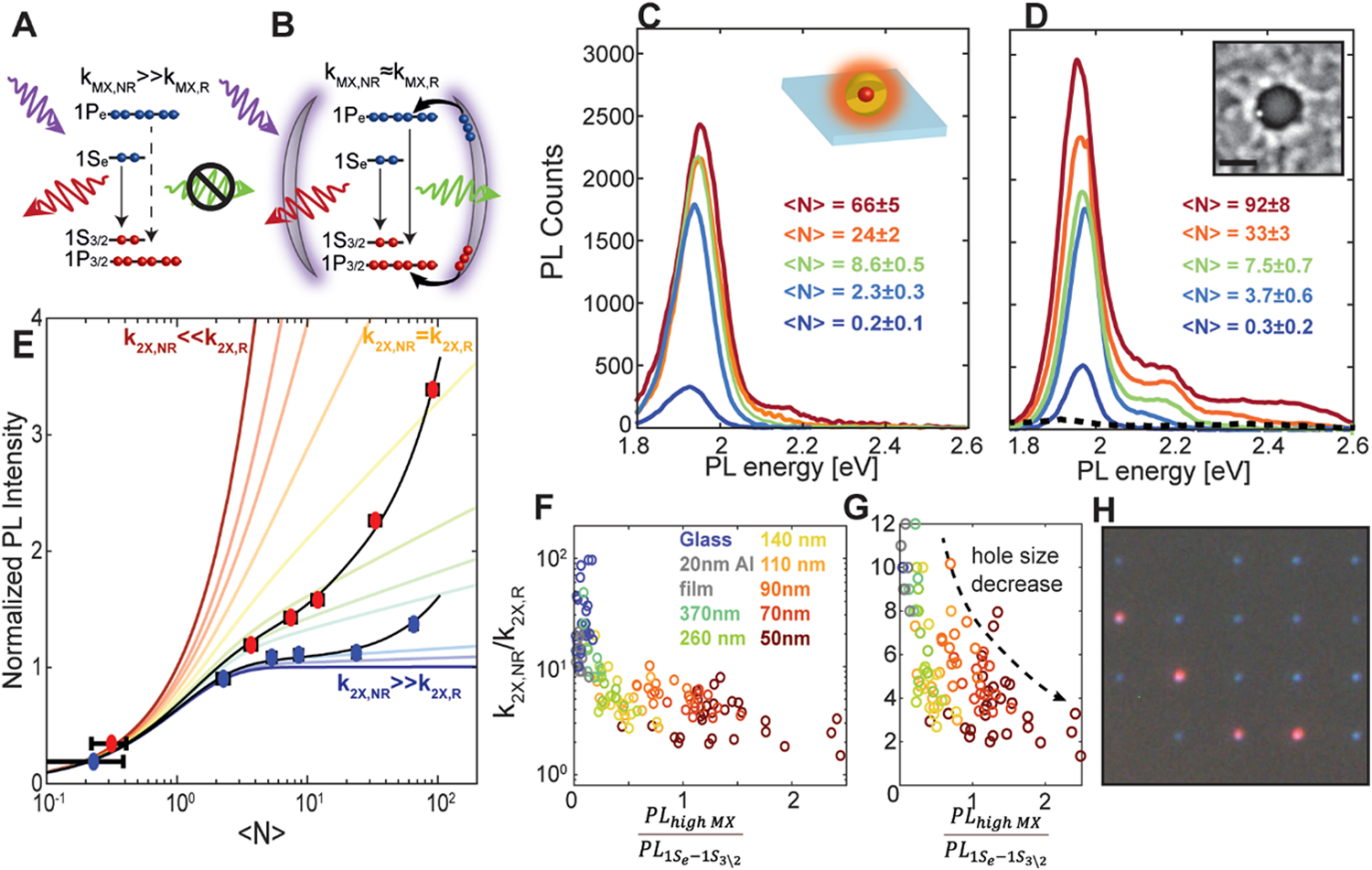
(A) Schematic of MX recombination in QDs: in MX states above the band edge, the nonradiative Auger process dominates. Thus, emission primarily arises from band-edge (1S_e_–1S_3/2_) recombination of single- or biexcitons. (B) Coupling a QD to a plasmonic metal cavity reduces the nonradiative/radiative rate ratio (*k*_MX,NR_/*k*_MX,R_), enhancing MX emission, and enabling additional QD-metal plasmon charge transfer. Emission then includes both band-edge photons (1.85–2.03 eV) and higher-energy states (above 2.04 eV). (C) PL spectra of a single QD on glass with increasing excitation power (in terms of ⟨*N*⟩ = average excitons per pulse, power normalized by the fitted absorption *σ̃*). High-energy MX emission (1P_e_–1P_3/2_) appears at ⟨*N*⟩= 66 ± 5, where its intensity is 5.7 ± 0.4% of the 1S_e_–1S_3/2_ PL. (D) PL spectra of a single QD in a 110 nm nanohole (inset SEM, scale 100 nm) in a 30 nm Al film. Here, the 1P_e_–1P_3/2_ to 1S_e_–1S_3/2_ PL ratio reaches 10.7 ± 0.6% at ⟨*N*⟩ = 3.7 ± 0.6. At ⟨*N*⟩ = 33 ± 3, the ratio reaches 43 ± 3% and continues rising. The dashed black line is a reference PL spectrum from a nanohole without any QDs at 760 fJ per pulse excitation power. (E) Normalized PL versus Poissonian ⟨*N*⟩, with colored lines showing simulated saturation for various *k*_2X,NR_/*k*_2X,R_ ratios, from Auger-free MX emission (red) to single-exciton-limited emission (blue). Circles show data from the QDs on glass (panel C, blue), matching the PL saturation trend and ⟨*N*⟩ dependence reported in previous works,^7^ and within the Al nanohole (panel D, red), respectively, with error bars from laser power measurements. Black lines are fits including a linear rise above ⟨*N*⟩ ≈ 20 corresponding to the quasi-CW excitation regime. (F) *k*_2X,NR_/*k*_2X,R_ from saturation fits for single QDs on glass (dark blue), thin Al film (gray), and nanoholes of various diameters ranging from 370 to 50 nm (green-red), plotted against $\frac{{\mathrm{PL}}_{\mathrm{highMX}}}{{\mathrm{PL}}_{1S_{e}-1S_{3/2}}}$ ratio, at 760 fJ per pulse excitation power (⟨*N*⟩>40). (G) Close-up of the low *k*_2X,NR_/*k*_2X,R_ values in F related to QDs in different-sized nanoholes. (H) Wide-field PL and brightfield image of the QD-hole substrate, with QD-filled holes (red PL) and empty holes (faint blue transmission).

For the QD in the 110 nm nanohole, the ratio of $\frac{{\mathrm{PL}}_{\mathrm{highMX}}}{{\mathrm{PL}}_{1S_{e}-1S_{3/2}}}$ reaches 0.43 ± 0.03 at ⟨*N*⟩ = 33 ± 3 (286 fJ), more than an order of magnitude higher than measured on glass at doubled ⟨N⟩. We also note that QDs in nanoholes exhibit reduced PL intensity fluctuations. This behavior has been attributed to QY equalization between charged and neutral excitons within the same QD,^58^ and a similar equalization is expected to extend to higher-energy MX transitions as well. The effect is even more pronounced for QDs with thinner CdS shells (Supporting Figure S5). The saturation of a single QD’s PL intensity with increasing excitation power has been shown for QDs of similar size and structure^6,7,59^ following the model

1 $$I=\sum_{N=1}^{\infty }\mathrm{Pois}\left(N,\left\langle N\right\rangle \right)\sum_{m=1}^{N}Q_{\mathrm{mX}}$$

where *I* is the normalized PL intensity, Pois is the Poissonian probability to excite *N* excitons in the QD at a laser power exciting ⟨*N*⟩ average excitons per pulse, and *Q*_mX_ is the QY of the *m* ^th^ exciton, which can be modeled as $Q_{\mathrm{mX}}={\left(1+\left(m-1\right)\frac{k_{2X,\mathrm{NR}}}{k_{2X,R}}\right)}^{-1}$, assuming both radiative and nonradiative rates scale linearly with the number of available recombination pathways.^7,8,60^ The parameter $\frac{k_{2X,\mathrm{NR}}}{k_{2X,R}}$ is the ratio between the nonradiative Auger recombination and the radiative recombination pathways of the BX state, which is proportional to the overlap between the carrier wave functions. Figure 1e shows colored lines of the plotted model in eq 1 for different values of $\frac{k_{2X,\mathrm{NR}}}{k_{2X,R}}$ . The total PL intensity for both QDs (circles, glass-blue, nanohole-red), measured using an avalanche photodiode (APD), and normalized to the 1X saturation PL intensity value, is plotted on top. eq 1 remains valid only while MX recombination lifetimes are longer than the laser pulse width (approximately 100 ps for our lasers). Beyond this point, more than one excitation cycle can occur within a single pulse, leading to a quasi-continuous wave excitation regime for the QD. Using these assumptions, we use an adjusted equation to fit our measured PL intensity

2 $$I_{\mathrm{PL}}=Q\cdot \left(\sum_{N=1}^{\infty }P\left(N,{\mathrm{\sigma ̃}\cdot p}_{\mathrm{Laser}}\right)\sum_{m=1}^{N}{\left(1+\left(m-1\right)\frac{k_{2X,\mathrm{NR}}}{k_{2X,R}}\right)}^{-1}\right)+C\cdot p_{\mathrm{Lase}r}\left(1-e^{-p_{\mathrm{Laser}}/p_{\mathrm{CW}}}\right)$$

The first term in the equation corresponds to the pulsed saturation model discussed earlier, with a PL 1X intensity fit parameter Q, where ⟨*N*⟩ = *σ̃*·*p*_Laser_ is the average number of excitons created per laser pulse. Here, *p*_Laser_ is the laser pulse power, and *σ̃* is the excitation pulse area normalized absorption cross section, where *σ̃^–^* ^1^ represents the laser power needed to generate one exciton in the QD on average. The second term introduces a laser power-dependent linear contribution^61^ above a threshold power *p*_CW_, which accounts for quasi-continuous excitation when *p*_Laser_ exceeds a regime in which ⟨*N*⟩ excitons can be generated with MX decay times shorter than the pulse duration, adding a laser power-dependent rise in the PL due to multiple excitation cycles within the pulse.^7^ Because these ultrafast MX transitions make up only a small fraction of the excited-state dynamics, their linear contribution to the PL dependence of a dipole emitter is captured by the constant C, which incorporates the effective absorption and emission-rate terms under CW excitation.^62^ Including this linear term in *I*_PL_ is particularly important for accurately extracting *σ̃* and $\frac{k_{2X,\mathrm{NR}}}{k_{2X,R}}$ in smaller nanoholes (Supporting Figure S4), where PL decay times are significantly faster than on glass.

By fitting the data to eq 2 (details and extracted values of $\mathrm{\sigma ̃},Q,\frac{k_{2X,\mathrm{NR}}}{k_{2X,R}},C,\mathrm{and}\;p_{\mathrm{CW}}$ are provided in Note S1 and Figure S4), we determine ⟨*N*⟩ for each QD configuration at every excitation power and compare the $\frac{k_{2X,\mathrm{NR}}}{k_{2X,R}}$ ratios. For the QD on glass, the dominance of nonradiative Auger decay over radiative recombination $\frac{k_{2X,\mathrm{NR}}}{k_{2X,R}}=30\pm 10,k_{2X,\mathrm{NR}}\gg k_{2X,R}$ limits the PL output to mainly the 1S_e_–1S_3/2_ single- and biexciton transitions. In the nanohole, the fitted ratio drops to $\frac{k_{2X,\mathrm{NR}}}{k_{2X,R}}=6.2\pm 0.7$ , indicating a substantial increase in MX radiative QY. As a result, higher-order MX states contribute more strongly to the PL spectra. The same analysis was carried out for QDs on glass, on a 20 nm thick Al film, and in nanoholes of different diameters. Extracted $\frac{k_{2X,\mathrm{NR}}}{k_{2X,R}}$ values from the PL saturation fits versus high excitation power $\frac{{\mathrm{PL}}_{\mathrm{highMX}}}{{\mathrm{PL}}_{1S_{e}-1S_{3/2}}}$ ratios of single QDs are shown in Figure 1f. A clear trend shows a decrease in $\frac{k_{2X,\mathrm{NR}}}{k_{2X,R}}$ correlates with increasing $\frac{{\mathrm{PL}}_{\mathrm{highMX}}}{{\mathrm{PL}}_{1S_{e}-1S_{3/2}}}$ . QDs on glass or on a thin Al film (blue and gray circles, respectively) exhibit the lowest $\frac{{\mathrm{PL}}_{\mathrm{highMX}}}{{\mathrm{PL}}_{1S_{e}-1S_{3/2}}}$ values and the highest $\frac{k_{2X,\mathrm{NR}}}{k_{2X,R}}$ ratios. In the Al nanoholes, QDs coupled to smaller nanoholes yield higher $\frac{{\mathrm{PL}}_{\mathrm{highMX}}}{{\mathrm{PL}}_{1S_{e}-1S_{3/2}}}$ ratios and lower $\frac{k_{2X,\mathrm{NR}}}{k_{2X,R}}$ values (Figure 1g, presenting a close-up to the relevant area in Figure 1f).

These measurements specifically focused on nanoholes containing a single QD, verified through wide-field PL imaging (Figure 1h) and by post-measurement SEM imaging of each nanohole measured, confirming single versus multiple occupancy. Notably, comparing single and double QD occupancy in nanoholes of equal diameter (Supporting Figure S6) shows comparable PL saturation profiles and $\frac{{\mathrm{PL}}_{\mathrm{highMX}}}{{\mathrm{PL}}_{1S_{e}-1S_{3/2}}}$ intensity ratios. The main differences arise from changes in the overall absorption cross section and in the scaling of the 1X emission intensity, driven by variations in the *σ̃* and *Q* fit factors in eq 2. In some cases, nanoholes smaller than 90 nm containing more than two QDs manifested additional MX enhancement beyond that observed for single or double QD occupancy (Supporting Figure S7). This suggests that the fundamental MX enhancement mechanism remains consistent across different occupancy levels, underscoring metal-QD interactions as the primary driver in this system and supporting its use with both single and multiple quantum emitters.

### Energy Band Spectra and Temporal Assortment

To disentangle the different emission pathways in nanohole-coupled QDs, we examined their temporal characteristics. Utilizing a recently developed, heralded spectroscopy technique measuring PL of QD aggregates with a linear APD array coupled to a spectrograph,^63^ the temporal and spectral features of each QD emission event are resolved. This enables the direct identification of different emissive transitions based on differences in their correlated PL spectra and relaxation times.

Figure 2a shows time-resolved PL spectra from a QD-aggregate on glass, with each spectrum representing a 20 ps PL time-bin over a 2 ns post-excitation window. Immediately after excitation (purple shades), the PL reveals a spectrum containing up to five distinct features. For the QD sample used in this work,^63^ these comprise: (i) the lowest-energy 1X and BX emission within the 1S_e_–1S_3/2_ band at 1.93 ± 0.01 eV, (ii) a multiply charged exciton (CX) component from the same band that appears while higher-band energy states are also occupied, which leads to a pronounced blue shift of the emission due to even higher coulomb repulsion between the excited carriers (Δ*E* ≈ + 50 ± 10 meV), (iii) MX emission from the 1P_e_–1P_3/2_ band at 2.10 ± 0.02 eV, and (iv, v) two broader MX features linked to higher-orbital transitions at 2.23 ± 0.05 and 2.30 ± 0.05 eV. We note that the 1X-BX splitting in this system is 30 ± 10 meV,^63^ and for simplicity, we treat these contributions together.

**Figure 2:**
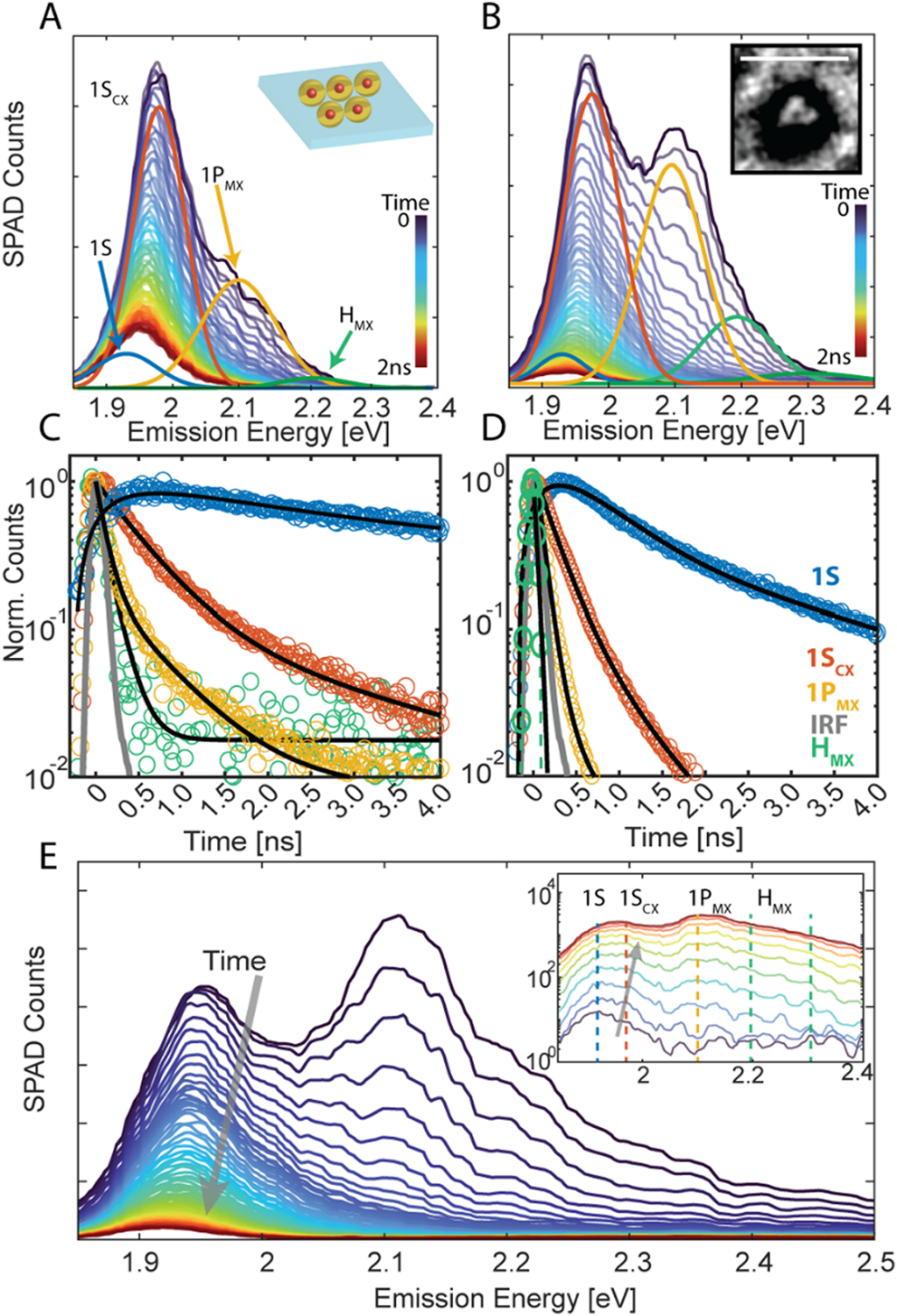
(A) Time-dependent PL spectra of a QD-aggregate on glass, excited at 102 fJ laser power per pulse, taken using a single-photon-avalanche-detector array (SPAD-λ), and 20 ps time binning. (B) Time-dependent PL spectra of a 70 nm diameter nanohole containing 3 closely packed QDs (corresponding SEM image in inset, scale is 100 nm), with the same laser power as in panel (A). Each PL spectrum in panels (A, B) is fit to a sum of 5 Gaussian peaks identified as the 1S_e_–1S_3/2_ transition of single and biexcitons (blue), 1S_CX_ transition (orange), 1P_e_–1P_3/2_ band MX transitions (yellow), and transitions from higher-energy bands (H_MX_, green). A detailed explanation of the 5 Gaussian fitting procedure and constraints is given in the Supporting Information Note S2. (C) Time-dependent decay of the distinguished PL peaks from the fits in panel (A). All peaks are fit according to the procedure described in Note S2, considering both single and multiexcitonic emission components from each energy band. (D) Time-dependent decay of the distinguished PL peak areas from the fits in panel (B) showing fast MX emission close to the instrument response function (IRF, gray), and a slower 1S 1X-BX decay with a rise time of 360 ± 20 ps (same in C,D) after the MX maxima, which can indicate state-filling decay up to that time. (E) Time-dependent PL spectra of the same QD-hole system in panel (B) at high excitation power (285 fJ in each laser pulse, approximated ⟨*N*⟩ =14 ± 4 using Supporting Figure S26). Here, the decay of the MX bands is clearly seen before decay from the 1S-band. The inset shows the opposite trend in log scale for the PL rise (at 20 ps time bins), establishing an exciton-accumulation state-filling emission mechanism.

Figure 2b shows a comparable measurement of a 70 nm Al-nanohole containing 3 aggregated QDs, under identical excitation conditions. While the same first three emission peaks appear for both glass and nanohole cases at matching energies, the higher-energy MX features (above the 1P_e_–1P_3/2_ band) are significantly suppressed for the QDs on glass, despite approximated high ⟨*N*⟩ values (⟨*N*⟩_glass_ = 15 ± 8, ⟨*N*⟩_70nm_ = 5 ± 1, based on the statistically averaged ⟨*σ̃*⟩ of full single QD saturation measurements shown in Supporting Figure S26) placing both systems within the MX regime. The 1P_e_–1P_3/2_ band peak is seen for the nanohole case already at low power excitation (7.8 fJ corresponding to ⟨*N*⟩ = 0.4 ± 0.1, Supporting Figure S8), where the probability of generating three or more excitons is very low.

The PL in the 0–2 ns window for QDs on glass is dominated by the 1S_e_–1S_3/2_ CX emission, consistent with previous reports,^64^ while features iii–v appear only weakly. This reflects strong MX suppression in glass-supported QDs, where rapid many-body Auger recombination outpaces the intrinsic MX radiative rate.^7^ Within the 0–2 ns window, the MX features (iii–v) of both QD cases decay almost entirely, accompanied by a red shift in the PL, such that only transitions from the 1S_e_–1S_3/2_ band persist at later times (red shades).

By integrating the fitted area of each peak across time bins, we obtain time-resolved PL decay dynamics for each emissive transition. Figure 2c,d compares biexponential fits for the two 1S_e_–1S_3/2_ band peaks and the 1P_e_–1P_3/2_ peak, showing consistently faster decay in the nanohole configuration (*D*) than for QDs on glass (C). On glass, the 1S decay follows a clear monoexponential profile with a 3.0 ± 0.6 ns lifetime, shorter than that expected for neutral excitons in single QDs due to contributions from charged 1X/BX emission and inter-QD dipole interactions. In contrast, QDs in the nanohole show a clear biexponential decay with time constants *t*_1_ = 0.61 ± 0.04 ns and *t*_2_ = 2.2 ± 0.1 ns, with nearly equal amplitudes (0.53 and 0.47). The additional fast component may arise from a dominant biexciton and Trion population within the 1S decay peak compared to QDs on glass, or from additional plasmon-induced interparticle energy transfer pathways between closely spaced emitters.^65^ The resulting average lifetime of 1.4 ± 0.1 ns is about half that measured on glass. Comparing the S-band CX and 1P_e_–1P_3/2_ MX lifetimes on glass (650 ± 50 ps and 210 ± 20 ps, respectively) with those in nanohole-coupled QDs (290 ± 40 ps and 100 ± 20 ps, respectively) shows that both channels decay 2-fold faster in nanoholes. For the higher-energy H_MX_ peaks (glass: 160 ± 20 ps; nanohole: 42 ± 6 ps), we observe a 4-fold acceleration in the nanohole, yet the decay time is shorter than the instrument response (70 ± 20 ps decay tail). This indicates a stronger plasmonic emission enhancement of the higher-energy MX transitions, although the exact magnitude may be affected by the limited signal-to-noise ratio of these peaks. The observed rate acceleration across all transitions suggests a broadband plasmonic enhancement spanning much of the visible spectrum, which remains significant even at high excitation powers where absorption saturation usually weakens such effects due to transition state filling.^62^ In both samples, the 1S-band 1X/BX peak rises more slowly than the MX peaks, with a 360 ± 20 ps rise time that reflects its origin in single and biexciton recombination, happening after relaxation of the higher-energy MX states. These effects are highlighted in Figure 2e, which shows the PL decay from the same nanohole at high ⟨*N*⟩, where the 1P_e_–1P_3/2_ emission temporarily surpasses the 1S-band PL immediately after excitation. The inset further illustrates the state-filling dynamics by plotting the PL rise on a logarithmic scale, showing how the emission evolves from low to high exciton occupancy (purple to red) within the laser pulse duration. At the start of the pulse, when the exciton population is still low, emission originates mainly from the 1X 1S_e_–1S_3/2_ state. As absorbed photons excite additional excitons during the pulse, higher-energy transitions become possible and dominate due to their rapid recombination time scales. In the final stage, the PL is again dominated by the 1S_e_–1S_3/2_ band emission due to its longer relaxation time scales. The inset therefore visualizes a clear state-filling process: early population of higher-energy states becomes radiatively accessible as lower states fill, temporarily preventing intraband relaxation paths and allowing high-energy emission to prominently emerge.

### Al Nanohole Size-Dependent MX Emission Enhancement

We continue with a systematic set of PL spectral and lifetime measurements on single QDs embedded in Al nanoholes of varying diameters, using excitation powers spanning from below the single-exciton threshold to the multiexciton regime. Figure 3a shows PL emission from single QDs in nanoholes with diameters ranging from 50 to 370 nm, excited with laser pulses corresponding to ⟨*N*⟩ < 0.3 based on the fitted absorption cross section. At this low ⟨*N*⟩, the probability of generating three or more excitons is below 0.36%. As the nanohole diameter decreases, the emission spectrum blue shifts markedly, with peak shifts of 52 ± 6 meV for a QD in a 50 nm Al nanohole relative to glass, and 74 ± 10 meV for a QD on a 20 nm Al film compared to glass. This shift suggests a dominant multiply charged 1S_e_–1S_3/2_ CX emission, despite occurring at ⟨*N*⟩ value too low to statistically populate this state for the QD alone.

**Figure 3:**
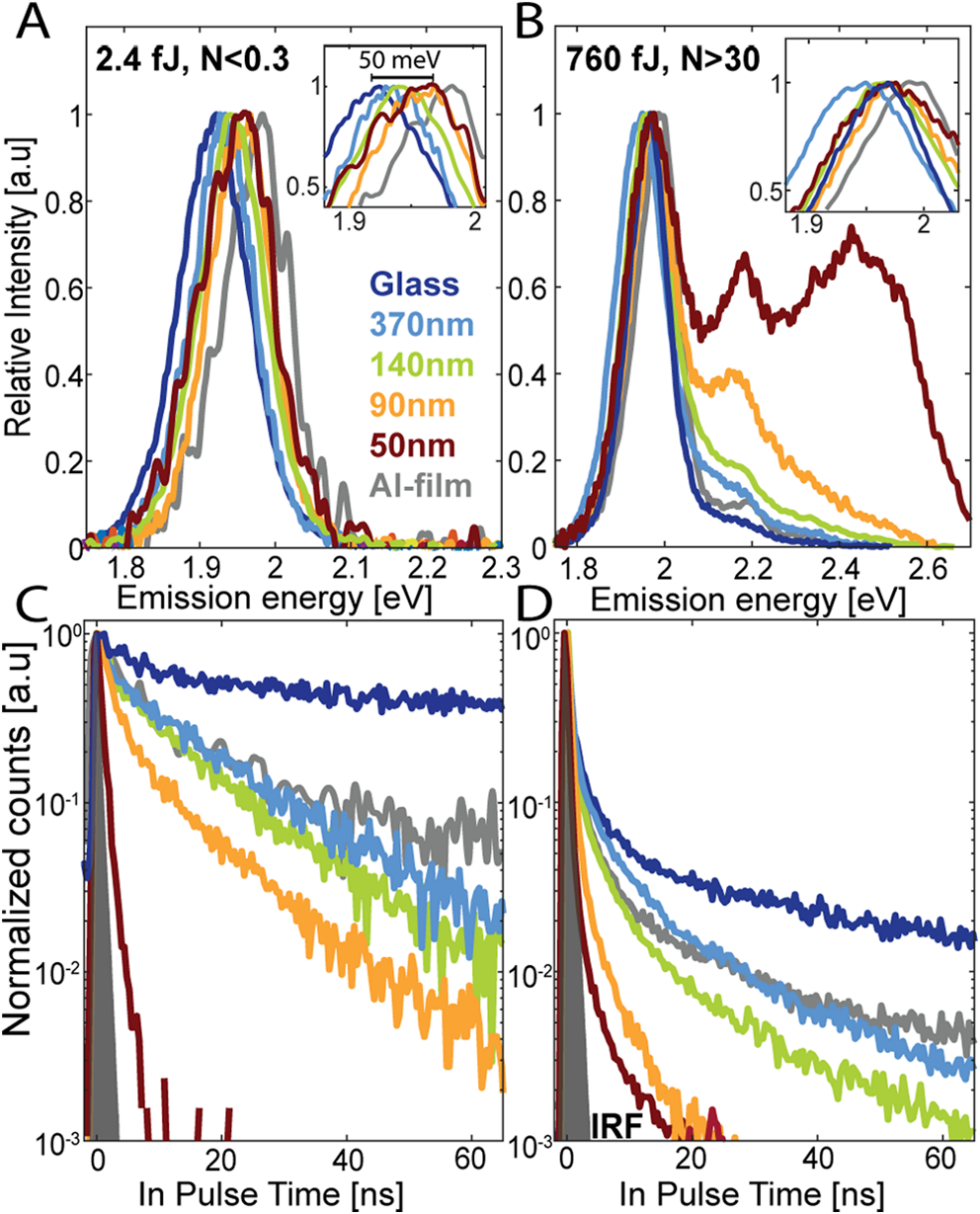
(A) Low-power (2.6 fJ) maxima-normalized PL spectra of a single QD on glass (dark blue) and in different diameter Al nanoholes (370 nm-light blue, 140 nm-green, 90 nm-purple, 50 nm-dark red, 20 nm Al film-gray). Inset: close-up of PL peaks showing a 52 ± 6 meV blue shift between a pristine QD on glass and a QD in a 50 nm diameter nanohole (dark red). Additional nanohole spectra show consistent PL blue-shifting with decreasing hole size. (B) High-power (760 fJ) maxima-normalized PL spectra of the same QDs in panel (A). The 1S_e_–1S_3/2_ peak position (inset) remains constant, indicating full state filling of this band in all cases. The $\frac{{\mathrm{PL}}_{\mathrm{highMX}}}{{\mathrm{PL}}_{1S_{e}-1S_{3/2}}}$ intensity ratios are 0.12 ± 0.04 (glass), 0.24 ± 0.07 (370 nm), 0.40 ± 0.07 (140 nm), 1.0 ± 0.1 (90 nm), 2.4 ± 0.2 (50 nm), and 0.13 ± 0.05 (Al film). (C) PL decay curves from the QDs measured in panel (A), fitted with a biexponential model with weighted lifetimes: τ_glass_ = 88 ± 3 ns, τ_370 nm_ = 12.5 ± 0.8 ns, τ_140 nm_ = 9.1 ± 0.5 ns, τ_90 nm_ = 5.2 ± 0.4 ns, τ_50 nm_ = 0.9 ± 0.1 ns, τ_Al‑film_ = 11 ± 2 ns. (D) PL decay curves for B: τ_glass_ = 5.8 ± 0.6 ns, τ_370 nm_ = 1.9 ± 0.2 ns, τ_140 nm_ = 1.4 ± 0.2 ns, τ_90 nm_ = 1.0 ± 0.2 ns, τ_50 nm_ = 0.7 ± 0.2 ns, τ_Al‑film_ = 2.1 ± 0.2 ns. APD instrument response (τ_IRF_ = 0.7 ± 0.1 ns) shown in semitransparent gray-fill.

At high excitation power (Figure 3b), corresponding to ⟨*N*⟩ > 30, the 1S_e_–1S_3/2_ peak energy varies slightly without correlation to hole size, indicating full saturation of this transition, accompanied by a rise in higher-energy MX band emission as the hole size decreases (15-fold enhancement in the 50 nm hole relative to the QD-on glass). Time-resolved PL decay measurements follow the same trend, with the QD on glass (dark blue) exhibiting a lifetime 7 times longer than that in the 370 nm nanohole (light blue), and 100 times longer than in the 50 nm nanohole (dark red) under low excitation power (Figure 3c). At high excitation power (Figure 3d), all hole sizes exhibit a similar fast decay component associated with IRF-limited MX recombination. Variations in the slower decay component result in smaller differences in the extracted lifetimes: 5.8 ± 0.6 ns for the QD on glass, 1.9 ± 0.2 ns for the 370 nm nanohole, and 0.7 ± 0.2 ns for the 50 nm nanohole, the latter limited by the APD response time (IRF-gray). Measurements of the QD PL spectra in sub-70 nm Al nanoholes show that, with increasing laser power quantified by ⟨*N*⟩, the 1S_e_–1S_3/2_ CX emission rises and saturates at 1<⟨*N*⟩<4 (Supporting Figure S9). Beyond this range, the spectrum is increasingly dominated by 1P_e_–1P_3/2_ and higher-energy MX transitions. The nanostructured near fields at the metal–dielectric interface may also modify the optical selection rules in QDs,^66^ enabling otherwise forbidden transitions as an alternative pathway for emission enhancement. However, no corresponding spectral shift to higher-energy emission has been reported in previous works in Al nanoholes with diameters above 130 nm,^67,68^ suggesting that the enhanced MX emission in those works primarily originates from biexcitons within the 1S_e_–1S_3/2_ band, which emit at nearly the same energy as the 1X transition. This can indicate nonradiative energy transfer to the metal, which preferentially quenches 1X over BX emission. A comparison of integrated PL peak areas (Supporting Figure S10) in our system shows that 1S emission is about two times weaker for QDs in Al nanoholes compared to glass. In contrast, CX S-band emission is over 10-fold stronger, with peak values at 90–110 nm nanohole diameters. MX emission from higher-energy bands is more than 80-fold higher, with a maximum at 50–70 nm, under moderate excitation. Surprisingly, the QD on the Al film shows here the lowest high-energy MX emission ratio and longest PL lifetimes after glass, indicating that high-energy MX transition rates are only weakly enhanced on the Al film.

### QD In-Cavity Location Correlated with MX Emission

Plasmonic enhancement of multiphoton emission from QDs has been demonstrated using various metallic nanostructures.^34,68–70^ These studies reported increased photon bunching at zero-time delay, measured with two APDs in a Hanbury Brown–Twiss (HBT) setup. The resulting second-order correlation function’s *g* ^2^(0) value reflects the biexciton-to-single-exciton QY at very low ⟨*N*⟩.^71^ An increase in *g* ^2^(0) has been observed with increased proximity between the metal nanostructures and QDs.^69,72^ This increase is attributed to the stronger resonant optical fields near the metal–dielectric surface, which disproportionately enhance the radiative transition rates of MX states compared to 1X due to the *|E|* ^2*n*^ field dependence of the n^th^ exciton’s emission rate in the QD-cavity.^34^

Figure 4a presents the second-order correlation functions of a single QD on glass (blue) and on the edge of an Al-nanohole (red), measured at various excitation powers corresponding to increasing ⟨*N*⟩ values. Despite the higher ⟨*N*⟩ values for the QD on glass, the *g* ^2^(0) contrast is substantially greater for the nanohole-coupled QD. Statistical analysis across QDs in various hole sizes shows a general increase in *g* ^2^(0) values, correlated with higher MX emission intensity and shorter PL lifetimes (Supporting Figures S11,S12), though results vary significantly also among QDs of the same nanohole size, with single QDs in some of the 370 nm nanoholes showing *g* ^2^(0) values under 0.1 at 2.4 fJ, similar to the pristine QDs on glass. Figure 4b presents representative examples of the *g* ^2^(0) contrast increasing with laser pulse power for QDs on glass and in two nanohole size regimes, highlighting substantial differences at low excitation powers, and between single QDs within nanoholes of the same diameter.

**Figure 4:**
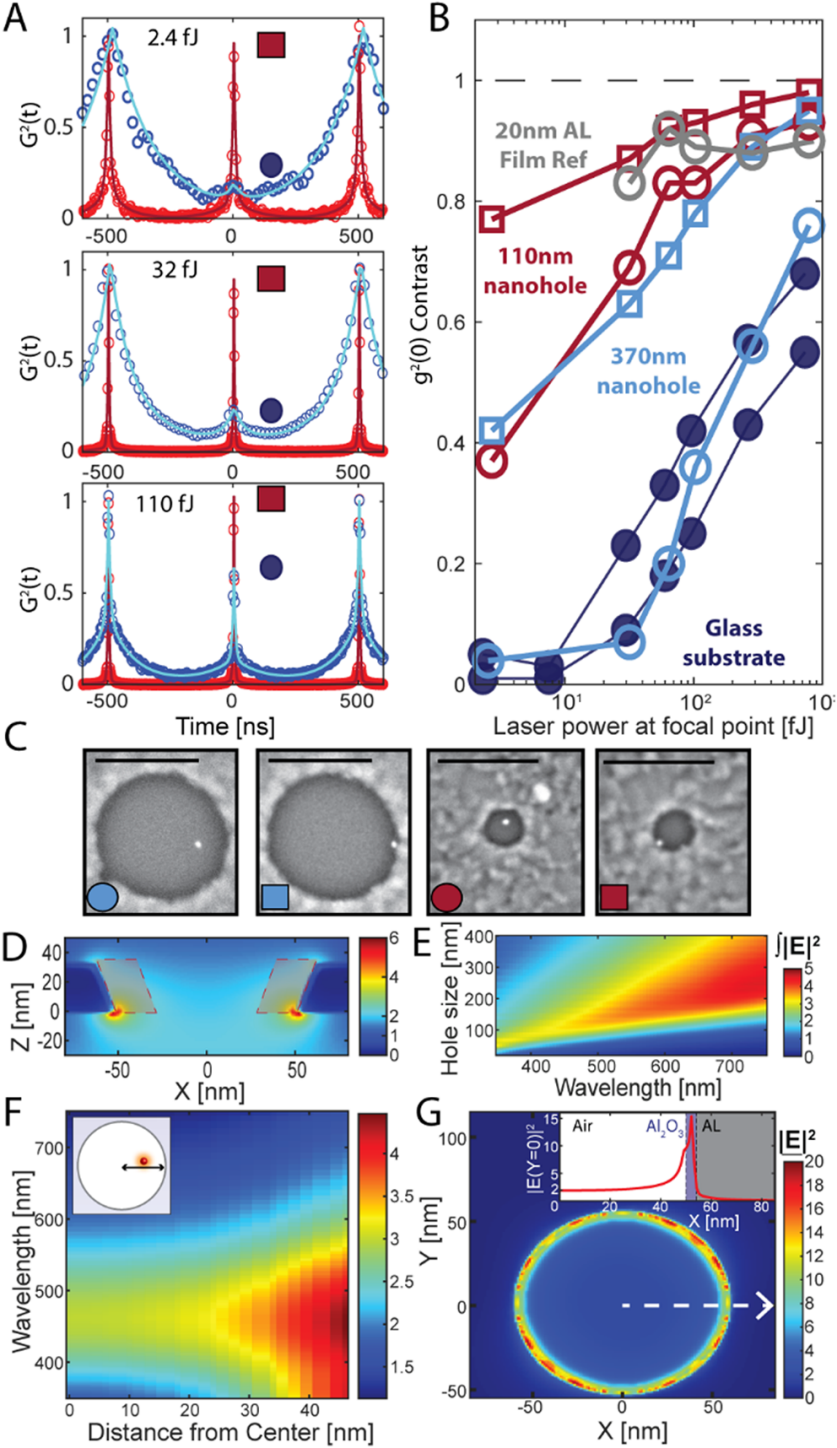
(A) Second-order coincidence counts *G* ^2^(t) (circles) for a QD on glass (blue) and a QD adjacent to the sidewall of a 110 nm diameter nanohole (red), at 3 different laser excitation powers (in fJ per pulse). Lines are the fitted correlation function from which the *g* ^2^(0) contrast is calculated. (B) *g* ^2^(0) saturation versus laser-point excitation power for 2 reference QDs on glass (dark blue circles), reference QD on 20 nm Al film (gray circles), a QD on the bottom of a 370 nm hole (light blue circle), a QD on the sidewall of a 370 nm hole (light blue squares), a QD on the bottom of a 110 nm hole (dark red circles), and a QD on the sidewall of a 110 nm hole (dark red squares) with its *G* ^2^(t) function is shown in panel (A). The calculated ⟨*N*⟩ values at 2.4 fJ are 0.41/0.39 ± 0.01 (glass, dark blue), 0.12, 0.08 ± 0.01 (light blue circle/square, respectively), and 0.32, 0.18 ± 0.02, 0.04 (dark red circle/square, respectively), all in the subexciton excitation regime. (C) SEM images of the QD locations in holes shown in panel (B) (respective marker shape and color); scale bar is 300 nm. (D) XZ plane simulation of |*E*| of a 405 nm wavelength X polarized plane-wave source, showing enhanced density on the sides and bottom of the 100 nm diameter (bottom diameter) hole. (E) Simulated |*E*|^2^ enhancement of a circularly polarized plane-wave source, on the 20 nm edge area of the nanohole sidewall, normalized to the edge area volume: $\left\langle {\left|E\right|}^{2}\right\rangle =\frac{\int {\left|E\left(r\right)\right|}^{2}}{\int r}$ , showing a broad enhancement in the excitation wavelengths and QD emission spectral range. (F) simulated radiative rate enhancement of a polarization-averaged dipole emitter in a 100 nm diameter hole in Al film, changing the dipole *X* location relative to the hole’s center. (G) *XY* plane simulation of |*E*|^2^ of a 405 nm wavelength circularly polarized plane-wave source at the bottom of the hole, showing higher field-density near the Al_2_O_3_ sidewalls (the inset is a 1D plot at *Y* = 0, following the white arrow).

Following SEM imaging of the same nanoholes (Figure 4c), corroborating single QD occupation, links *g* ^2^(0) enhancement with QD proximity to the nanohole wall. In the 370 nm nanohole measurements, a single QD positioned 92 nm from the metal wall (light blue circles, detailed analysis in Supporting Figure S13) shows *g* ^2^(0) values comparable to those of QDs on glass (dark blue circles). In contrast, another QD positioned adjacent to the Al wall (light blue squares) exhibits significantly higher *g* ^2^(0) values. A similar trend is observed for 110 nm nanoholes: a QD 38 nm from the wall shows lower *g* ^2^(0). At high excitation powers, the *g* ^2^(0) values of both QDs in the 110 nm holes converge, similar to the *g* ^2^(0) values of single QDs on an Al film (gray), which do not show significant MX PL. However, QDs in Al nanoholes positioned close to the sidewalls consistently display higher $\frac{{\mathrm{PL}}_{\mathrm{highMX}}}{{\mathrm{PL}}_{1S_{e}-1S_{3/2}}}$ values (Supporting Figure S14) and shorter lifetimes than the QDs in the glass part of the nanohole, or those adjacent to the walls of larger nanoholes.

This behavior can be partly linked to the higher density of photonic modes and stronger excitation fields at the metal–dielectric interface. To further support this, we ran two separate 3D finite-difference time-domain (FDTD) simulations. The first quantifies how the excitation laser interacts with the Al nanohole film, enhancing both the scattering cross section and the magnitude of localized near-field |*E*|^2^ near the cavity sidewall, boosting QD absorption (supporting Figures S16–S20, and 4d–g). The second examines how the radiative rate of a polarization-averaged dipole emitter inside the hole varies with its distance from the Al sidewall, as shown in Figure 4f.

These simulations confirm the presence of localized surface plasmon resonance (LSPR) modes with a relatively broadband spectral distribution whose near fields are predominantly localized along the sidewalls of the cavity, consistent with recent cathodoluminescence measurements,^73^ enhancing both excitation and radiative emission for QDs near the Al wall. However, the simulated |*E*|^2^ at the Al_2_O_3_–air interface varies very little with nanohole size decrease and is mostly dependent on the Z height in the nanohole (Supporting Figures S17–S20). Additionally, the calculated near-field dipole emission enhancement (Supporting Figure S23) is still 4–10-fold smaller than the seen lifetime shortening at sub-100 nm nanoholes (Supporting Figure S12), suggesting the role of additional recombination mechanisms. Although the S-band emission of QDs decreases near the Al wall due to ohmic losses that reduce the relative QY of both 1X and MX transitions, the $\frac{{\mathrm{PL}}_{\mathrm{highMX}}}{{\mathrm{PL}}_{1S_{e}-1S_{3/2}}}$ remains strongly enhanced in wall-coupled QDs as the hole size decreases, even when their *g* ^2^(0) values are similar. Measurements of rectangular nanoholes (Supporting Figure S24) show that $\frac{{\mathrm{PL}}_{\mathrm{highMX}}}{{\mathrm{PL}}_{1S_{e}-1S_{3/2}}}$ emission is strongest for QDs located near the structure corners, while both *g* ^2^(0) and the MX lifetime remain nearly unchanged. This spatial dependence on curvature and QD-metal contact area, combined with the observation of high-energy MX emission even at low excitation power, supports a plasmon-driven metal-to-QD charge injection pathway that contributes to the enhanced MX emission.

### Excitation Wavelength Dependence

Plasmonic excitations at metal–dielectric interfaces eventually decay, converting plasmon carrier energy into heat at subnanosecond time scales.^74,75^ This fast-heating process can, by itself, alter and enhance the semiconductor photophysics and state occupancy. Excitation-wavelength-dependent measurements can assist in distinguishing between these mechanisms.^75,76^ At low excitation power, the PL intensity follows the QD absorption profile, rising sharply at the band edge and increasing at higher energies due to larger state degeneracy, which increases the absorption cross section.^57,77,78^ Figure 5a shows power-dependent PL from a single QD excited at 405, 450, and 515 nm, in separate measurements under comparable pulse-power conditions. At high power, all three wavelengths produce similar emission levels, indicating MX PL saturation. However, saturation under 515 nm excitation requires much higher laser power. The fitted *σ̃* at 515 nm is an order of magnitude lower than at 450 and 405 nm, consistent with its 10-fold lower absorption (Supporting Figure S25), as 515 nm photons excite only the smaller CdSe core states while 450 and 405 nm photons also access the larger CdS shell. The slightly lower *σ̃* extracted for 405 nm, despite stronger absorption, has been linked to reduced PL QY at higher photon energies due to additional nonradiative pathways such as hot-carrier-induced surface trapping.^79–81^

**Figure 5:**
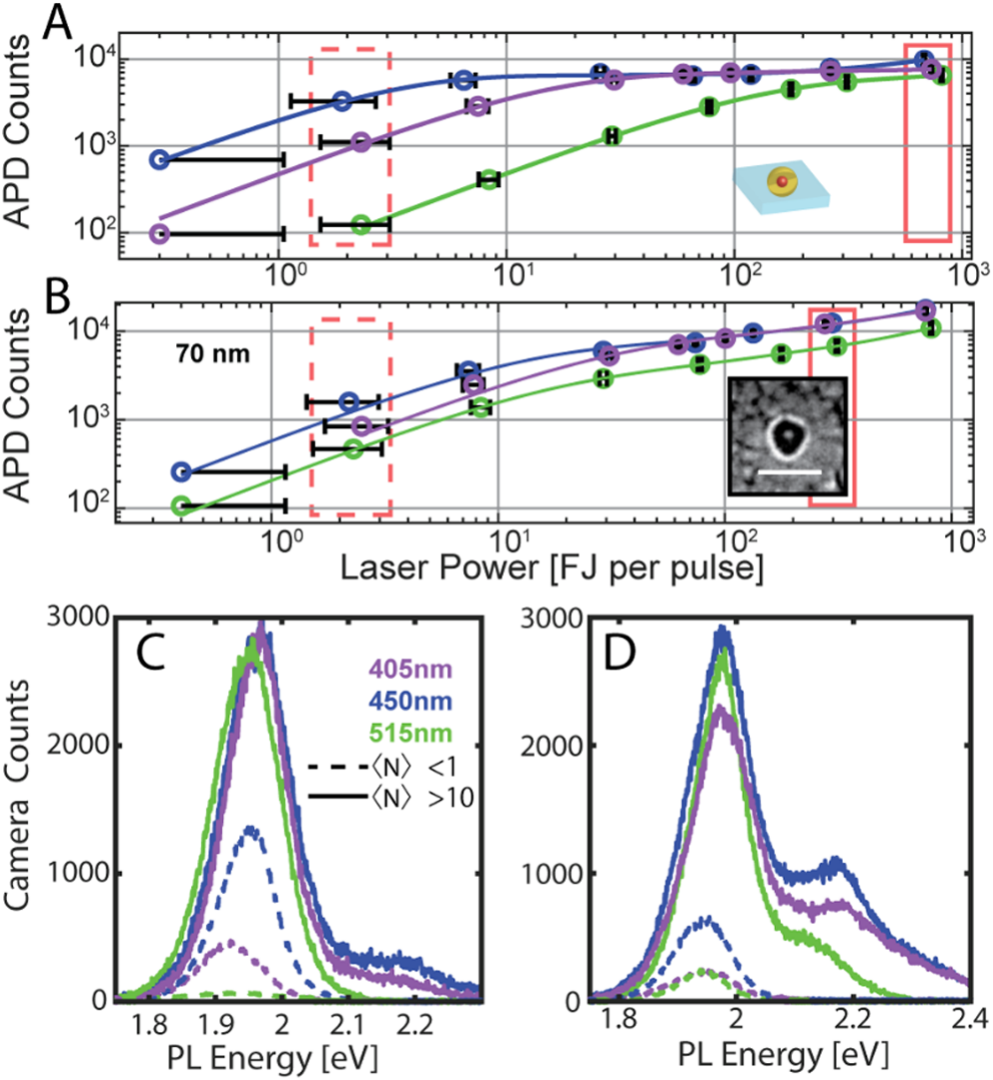
(A) APD-PL counts of a single QD on glass, versus laser power for 3 subsequent measurements with different pulsed laser wavelengths: 405 (purple), 450 (blue), and 515 (green) nm, fitted to eq 2. The fitted *σ̃* ^–1^ values are (fJ): 12.4 ± 0.2, 2.7 ± 0.5, 104.3 ± 0.4, for the 405, 450, and 515 nm wavelengths, respectively. (B) PL counts of a single QD in a 70 nm Al hole (inset, scale bar is 100 nm) measured at 3 different wavelengths. The fitted *σ̃* ^–1^ values are (fJ): 21 ± 2, 10 ± 1, 10.4 ± 0.1, for the 405, 450, and 515 nm wavelengths, respectively. The lower count rate for the 515 nm excitation is partially linked to the different long-pass filter (550 nm instead of 460 nm) used for the green laser measurement. (C, D) PL spectra of the measurements in panels (A, B) (red rectangles), at low power (dashed lines, ⟨*N*⟩<1) and high power (full lines, ⟨*N*⟩>10) excitation, with color indicating laser wavelength.

Repetition of the wavelength and power-dependent measurements on a single QD in a 70 nm Al nanohole (Figure 5b) shows that *σ̃* values decrease relative to glass for the 450 and 405 nm excitation, consistent with added laser reflection and absorption losses in the Al layer. Notably, the 515 nm excitation *σ̃* rises by an order of magnitude, becoming comparable to that of the 450 nm excitation. Figure 5c,d compares the PL spectra at low excitation fluence (2.3 fJ pulses, ⟨*N*⟩<1, dashed lines) and high excitation fluence (760–790 fJ on glass, 280–310 fJ in the nanohole, full lines) for the glass (Figure 5c) and nanohole (Figure 5d) cases. On glass, the changes in the relative intensities of the 1X-BX and MX transitions follow the effective ⟨*N*⟩ generated at each wavelength, reflecting the wavelength dependence of *σ̃*. Inside the nanohole, this dependence is less straightforward, with noticeable differences across both power regimes. Still, the $\frac{{\mathrm{PL}}_{1P_{e}-1P_{3/2}}}{{\mathrm{PL}}_{1S_{e}-1S_{3/2}}}$ ratio for 515 nm excitation is much closer to the values at 450 and 405 nm than on glass, since the effective ⟨*N*⟩ values produced at comparable excitation powers are closer.

Statistical comparisons of the fitted Q, *σ̃*, and the power-dependent $\frac{{\mathrm{PL}}_{1P_{e}-1P_{3/2}}}{{\mathrm{PL}}_{1S_{e}-1S_{3/2}}}$ for QDs on glass, and in 90 and 70 nm Al nanoholes (Supporting Figure S26), confirm enhanced absorption and exciton generation for the 515 nm low-energy excitation in sub-100 nm nanoholes, yielding higher PL output and $\frac{{\mathrm{PL}}_{1P_{e}-1P_{3/2}}}{{\mathrm{PL}}_{1S_{e}-1S_{3/2}}}$ ratios per excitation power. The approximately 10-fold increase in exciton generation observed for nanohole-coupled QDs under 515 nm excitation cannot be accounted for by the near-field absorption enhancement at the nanohole sidewalls, which remains relatively modest (∼2–3 fold) across the 405–515 nm range (Supporting Figure S17). This additional enhancement is therefore attributed to metal-QD interactions, involving charge and energy transfer pathways from the cavity, whose large absorption cross section supports strong broadband LSPR excitation, to the QD, effectively increasing its absorption cross section. These results demonstrate enhanced QD absorption at wavelengths that are otherwise weakly absorbed, enabled by the proximity to a strongly absorbing plasmonic nanocavity. Such enhancement can support efficient operation in applications that face limited absorption, including resonant band-edge excitation for coherent state tomography, solar-driven photocatalysis, and photovoltaics.

### MX Emission Enhancement in Different Metal Nanoholes

Our analysis of QD emission in Al nanoholes demonstrates significant MX enhancement under both low and high excitation powers. This enhancement is especially noticeable in smaller Al nanoholes, where the increased QD–metal contact area likely facilitates charge transfer.

To separate plasmonic effects from interband HCT in the observed MX enhancement, we next compare MX emission from QDs embedded in nanoholes formed in metal films with distinct plasmonic responses. We begin by measuring the power-dependent MX emission from single QDs in Al nanoholes spanning diameters from 370 nm down to 50 nm, using 405 nm excitation, and benchmarking against QDs on glass and on a 20 nm Al-on-glass film to isolate contributions arising specifically from localized surface plasmons (LSPR) in the nanoholes. Figure 6a shows the $\frac{{\mathrm{PL}}_{\mathrm{highMX}}}{{\mathrm{PL}}_{1S_{e}-1S_{3/2}}}$ ratio versus ⟨*N*⟩, with each colored line marking the averaged response of more than 10 QDs for each hole size and excitation power. On glass, PL_high MX_ remains very low and exceeds 5% only once ⟨*N*⟩ surpasses 20. This ⟨*N*⟩ threshold decreases slightly on the Al film, and drops sharply in Al nanoholes. In 50 nm holes, substantial PL_high MX_ already appears at ⟨*N*⟩ = 1. Such low ⟨*N*⟩ values barely populate the 1P_e_–1P_3/2_ or higher-energy excitonic states that normally give rise to PL_high MX_. Combined with the pronounced low-power 1S_CX_ blue-shifted PL for all AL-QD structures, these results indicate that the observed MX state filling cannot be explained by standard plasmon-enhanced absorption or emission, but instead reflects additional mechanisms introduced by the QD–metal nanohole environment.^62,67,68^

**Figure 6:**
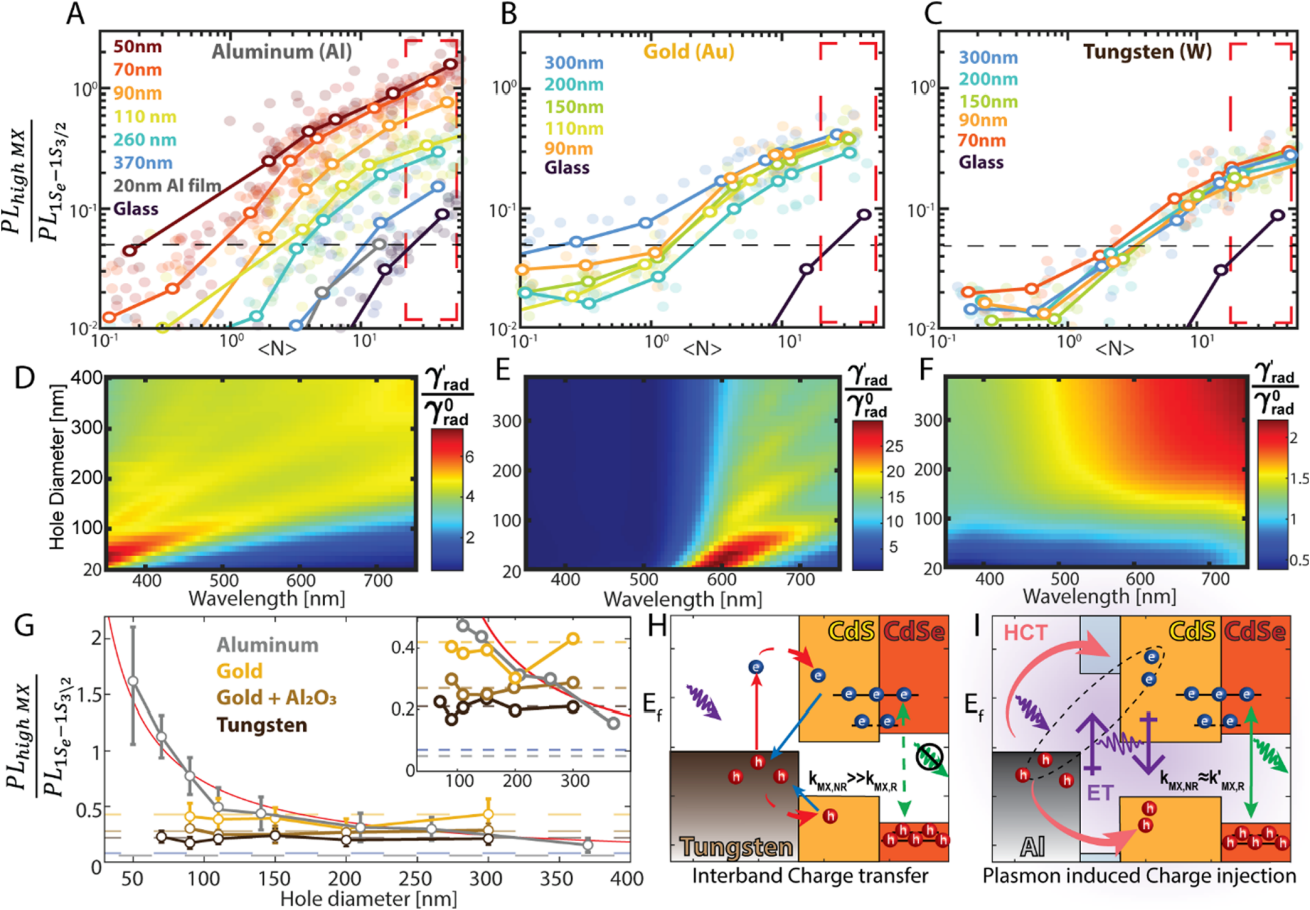
(A–C) $\frac{{\mathrm{PL}}_{\mathrm{highMX}}}{{\mathrm{PL}}_{1S_{e}-1S_{3/2}}}$ versus the average ⟨*N*⟩ populated excitons (log–log) from PL saturation fits (semitransparent circles), for QDs in Al nanoholes (A), Au nanoholes (B), and tungsten nanoholes (C), which do not support plasmon resonance in the visible spectral range. Averaged same nanohole diameter values are shown as thick lines with open circles. Colors indicate nanohole diameter. (D–F) Simulated radiative rate enhancement for a polarization-averaged dipole emitter 8 nm from a nanohole sidewall in a 30 nm Al film (D), gold film (E), and tungsten film (F), as a function of hole diameter. (G) high power (760 fj per pulse) $\frac{{\mathrm{PL}}_{\mathrm{highMX}}}{{\mathrm{PL}}_{1S_{e}-1S_{3/2}}}$ average of QDs in different metal nanoholes, versus nanohole diameter. Red line is an inverse diameter fit to the Al-nanohole data, slope = 69 ± 5 nm. Flat dashed lines are constants fit for the Au (0.42 ± 0.05), Au/Al_2_O_3_ (0.27 ± 0.04), and tungsten (0.21 ± 0.03) $\frac{{\mathrm{PL}}_{\mathrm{highMX}}}{{\mathrm{PL}}_{1S_{e}-1S_{3/2}}}$ data. The inset shows the low value $\frac{{\mathrm{PL}}_{\mathrm{highMX}}}{{\mathrm{PL}}_{1S_{e}-1S_{3/2}}}$ results, with reference glass $\frac{PL_{\mathrm{highMX}}}{{\mathrm{PL}}_{1S_{e}-1S_{3/2}}}=0.07$ (blue), and 20 nm Al film $\frac{{\mathrm{PL}}_{\mathrm{highMX}}}{{\mathrm{PL}}_{1S_{e}-1S_{3/2}}}=0.05$ (dashed gray). (H) Schematic energy band diagram for tungsten nanoholes showing only Interband-excitation charge transfer. Red (blue) arrows indicate forward (backward) transfer from metal to semiconductor. (I) Schematic energy band diagram for Al nanoholes (with native Al_2_O_3_ layer) with adjacent CdSe/CdS QDs, showing plasmon-induced charge transfer mechanism (electrons generated in CdS, holes in Al interface), and plasmon energy transfer (ET) exciting additional neutral excitons in the QD through nonradiative plasmon decay (purple arrows representing dipole–dipole interactions). In this case, the plasmon resonance also enhances the hot MX emission (green arrow).

FDTD simulations of the radiative enhancement (Figure 6d) and scattering spectra (Supporting Figure S21) for Al nanoholes with varying radii show a systematic blue shift and strengthening of the resonance field magnitude as the hole diameter decreases, reaching wavelengths below 400 nm for 50 nm holes, consistent with the hybridized surface plasmon polariton (SPP) modes of the film coupled with the LSPR modes of the hole.^82,83^ Enhancement is notably stronger when the dipole orientation lies in the film plane (*X*–*Y* direction), as it efficiently couples to LSPR modes confined within the hole, whereas the perpendicular orientation primarily interacts with SPP modes and undergoes energy dissipation into the metal film (see Supporting Figure S27). Despite the consistent trend and calculated values aligning with previous reports,^67,68^ the pronounced increase in the experimental emission rate and PL_high MX_ enhancement at very low ⟨*N*⟩ values are not fully captured by these simulations, pointing to the contribution of additional HCT and ET mechanisms contributing to PL_high MX_ band population.

Figure 6b shows the excitation power-dependent MX emission from QDs embedded in nanoholes within a 30 nm thick gold film, with diameters ranging from 300 to 90 nm. Unlike the Al-nanohole case, here, the ratio of $\frac{{\mathrm{PL}}_{\mathrm{highMX}}}{{\mathrm{PL}}_{1S_{e}-1S_{3/2}}}$ plotted against ⟨*N*⟩ shows no clear dependence on hole diameter and is markedly lower. Simulations of radiative rate enhancement in Au nanohole systems show that LSPR enhancement occurs at wavelengths above 570 nm (Figure 6e), with little dependence on hole size for diameters larger than 50 nm. Identical measurements were also performed on a second Au nanohole substrate coated with a 4 ± 1 nm Al_2_O_3_ layer (Supporting Figure S28). The oxide barrier, analogous to the Al native-oxide layer, leads to additional suppression of the $\frac{{\mathrm{PL}}_{\mathrm{highMX}}}{{\mathrm{PL}}_{1S_{e}-1S_{3/2}}}$ enhancement and displays negligible size dependence, consistent with simulation results.

A further set of experiments was carried out on nanoholes in a 30 nm tungsten (W) film. Owing to tungsten’s positive real refractive index across the visible range,^84,85^ this material cannot support surface or localized plasmon resonances within the studied spectral window. Consequently, only interband HCT contributes to MX state filling. QDs in W-nanoholes exhibit the lowest $\frac{{\mathrm{PL}}_{\mathrm{highMX}}}{{\mathrm{PL}}_{1S_{e}-1S_{3/2}}}$ ratios and longest decay lifetimes of all metallic nanohole systems investigated (Figures 6c,f,g and S29), and weak spectral variations in rate enhancement simulations, attributed to the metal-air refractive index contrast, with no plasmonic enhancement and negligible dependence on nanohole size.

Figure 6g presents the averaged $\frac{{\mathrm{PL}}_{\mathrm{highMX}}}{{\mathrm{PL}}_{1S_{e}-1S_{3/2}}}$ ratios as a function of nanohole diameter for the different metal films under high excitation power (760 fJ, ⟨*N*⟩ = 20–60). Al nanoholes display a clear inverse-diameter (1/D) trend, with a sharp rise in MX emission for diameters below 110 nm. This behavior matches stronger LSPR confinement in smaller holes, where enhanced mode compression produces a blue-shifted, broadened resonance and faster energy dissipation.^82^ In contrast, nanoholes in the other metals show weaker MX emission that remains nearly unchanged with diameter, though still elevated relative to QDs on glass or on Al films with the native oxide barrier.

While all metal nanoholes exhibit some contribution from nonplasmonic interband tunneling HCT (Figure 6H), including those with an added 4 nm oxide barrier, this process alone is nearly an order of magnitude weaker in non-plasmonic tungsten nanoholes compared to sub-90 nm Al nanoholes. The broadband absorption of Al nanostructures gives rise to plasmon oscillations, which can relax by generating electron–hole pairs at the metal–semiconductor interface. In this process, hot charges preferentially transfer to the QD; electron transfer is more probable due to the low CB offset, with the thin Al_2_O_3_ barrier possibly suppressing back-transfer, increasing the multiply charged exciton population at low excitation powers (Figure 6I). In sub-90 nm Al nanoholes, strong QD-cavity coupling enables plasmon decay pathways that resonantly excite additional excitons in the QD via plasmon-dipole ET, effectively enhancing the QD absorption cross section to approach that of the Al cavity, as reflected in the excitation-wavelength-dependent measurements. The observed multiexciton PL enhancement arises from faster radiative decay of high-energy multiexciton states relative to single-exciton emission. These MX states are efficiently populated by photon absorption and additional plasmon-driven HCT and ET. In parallel, near-field enhancement, ultrasmall mode volumes, and strong QD-metal coupling boost radiative MX recombination rates while suppressing multicarrier Auger losses.

### Conclusions and Outlook

We demonstrate a significant enhancement of hot MX emission in QDs coupled to individual Al nanohole cavities. Spectral and temporal PL analyses resolve the 1X-BX and multiply charged transitions within the 1S_e_–1S_3/2_ band, along with MX emission from the 1P_e_–1P_3/2_ and higher-energy bands, which show emissive transitions once the 1S_e_–1S_3/2_ band-states are fully occupied, preventing rapid relaxation to the band edge. The broad plasmon resonance of Al nanoholes enables simultaneous cavity coupling to different energy QD transitions, shown across a 130 nm spectral range. MX emission QY increases sharply with stronger QD-cavity coupling strength, achieved by reducing nanohole diameters below 90 nm. Simulations with varying nanohole sizes, together with excitation power and wavelength-dependent PL measurements, show that smaller nanoholes promote stronger MX emission through increased photonic mode density and enhanced near-field effects. This is supported by the experimentally observed an ∼4-fold reduction in the H_MX_ lifetime in 70 nm nanoholes, compared to only an ∼2-fold reduction for lower-energy transitions. However, the observed blue shift of the 1S_e_–1S__3_/_2__ transition at excitation powers corresponding to low ⟨*N*⟩, and the ultrashort PL lifetimes seen in sub-90 nm holes (2 orders of magnitude shorter than on-glass QDs), indicate the presence of an additional MX PL enhancement pathway. Scanning electron microscopy shows that enhanced MX emission correlates with QD placement adjacent to the nanohole wall, with MX emission intensity increasing with QD-metal contact area for apertures of similar size.

Experiments conducted across metal nanoholes supporting distinct visible-range LSPR responses indicate that MX enhancement occurs in cavities, which support plasmon-mediated interactions between the metal and the adjacent QD, which boosts emissive state filling at low excitation powers and suppresses MX nonradiative recombination at high powers. Excitation-wavelength-dependent measurements further verify the non-thermal plasmonic HCT and ET origin of the MX enhancement and reveal a 10-fold increase in QD absorption forlow-energy wavelengths that are weakly absorptive for pristine QDs, due to the highly absorptive metal surroundings. These results establish Al nanohole cavity-QD systems as an accessible and cost-effective platform for exploring cavity light-matter interactions beyond the weak coupling limit, manifesting plasmon-driven hot charge and energy transfer as an efficient pathway for enhancing broadband QD emission from hot MX transitions. By activating otherwise quenched MX states, this approach enables color-tunable PL from individual QDs in plasmonic cavities, with potential applications ranging from photocatalysis, chip-scale light sources and detectors, tunable microlasers, and correlated multiphoton sources for quantum technologies.

## Methods

### Fabrication of Nanohole Cavity Arrays

Nanohole arrays with diameters ranging between 50 and 400 nm in thin metal films were fabricated either by an electron lithography lift-off process or by an ion milling process using a scanning electron microscope (SEM) equipped with a dual beam focused ion beam (DB-FIB, Helios Nanolab 460F1Lite) that allows ion milling of high-precision nanoscale features in metal films. Special care was taken in the ion milling process to not mill into the bottom SiO_2_ layer, and the resulting nanohole arrays show the same properties as the nanoholes made by the lithography lift-off process (where accurate nanohole features were made down to 80 nm diameter).

Aluminum film thickness was chosen at 30 nm, which transmits less than 5% of the excitation light to QDs not within the nanoholes, while still minimizing QD emission loss (see Supporting Figure S30).

Standard glass cover slides (22 × 22 mm^2^ BOROFLOAT 33 glass) of 175 μm thickness were cleaned in a Piranha solution for 10 min. For the FIB nanohole milling process, a layer of aluminum is evaporated (Angstrom Quantum Series evaporator) on the cleaned substrates, and nanohole patterns containing 15 × 15 nanoholes and an orientation marker are milled with the gallium ion beam. For the E-beam lithography and liftoff process, a 300 nm thick layer of negative E-beam resist, ma-N 2403 (Micro Resist Technology), was spin-coated on the cleaned substrates and prebaked at 90 °C for 1 min. Due to the substrate’s lack of conductivity, a conductive polymer, E-Spacer 300Z, was spin-coated at 2000 rpm for 30 s on top of the baked E-beam resist. Afterward, the substrate was exposed to 100 keV at 500 pA E-beam using ELS-G100, Elionix E-Beam Lithography System, to form the desired patterned matrices of different sized, holes, and rectangles. After the exposure, the E-Spacer was removed by immersion in deionized water for 1 min, followed by blow-drying the substrate with N_2_ gas. Then, the resist was developed for 30 s with ma-D 525 (Micro Resist Technology) and immediately rinsed with DI water and dried with N_2_ gas to reveal 300 nm pillars of the exposed resist in the desired pattern. Afterward, a 30 nm layer of aluminum is evaporated (Angstrom Quantum Series evaporator) on top of the cover slide, followed by a liftoff process. The cover slide was immersed overnight in *N*-methylpyrrolidone (NMP) to dissolve the resist pillars, and then sonicated for a few minutes for complete removal of the resist pillars, creating holes in the aluminum in the desired shape and size. For the aluminum oxide-covered Au nanohole substrates, the same process was applied, followed by 5 nm of of Al_2_O_3_ using atomic layer deposition (ALD) Savannah G2, Vecco.

### QD Synthesis and Deposition in Metallic Nanoholes

CdSe/CdS QDs with core radius/shell thickness dimensions of 2.2/5.5 nm were synthesized following a well-established procedure.^86^ An ensemble characterization (TEM size statistics, absorption, and PL spectra in solution) is shown in Figure S25. The coupling of QDs to the nanohole walls is done by drop-casting 25–100 μL of QDs dispersed in a 1/9 hexane:octane solution, onto the glass substrates with the nanoholes, while monitoring the QD filling in our wide-field inverted PL microscope setup, to see when a sufficient amount of nanoholes have QDs inside them, seen by a red PL spot in the nanohole location. The ultralow QD concentration (∼10^4^ dilution from a 1–10 nM stock solution concentration, sonicated for 5 min to reduce particle clustering) reduces the possibility of multiple QDs within or near a single nanohole. Nevertheless, all nanoholes optically measured in this work were later imaged by SEM to know the exact location and quantity of QDs near them, and selected nanoholes were cut out of the substrate and put on a FIB-lift-out TEM-grid, as detailed in previous works.^55,56^

### Electron Microscopy

Scanning electron microscopy (SEM) of nanoholes containing QDs was performed using an Apreo 2S SEM (Thermo Fisher Scientific) in immersion mode at 5 kV, 0.1 nA, and an ∼6 mm working distance. Images were acquired using the T1 detector optimized for backscattered electrons (BSE), providing high Z-contrast that enables differentiation between the metal film and the coupled CdSe/CdS QDs. High-resolution scanning transmission electron microscopy (STEM) was done on nanohole cross sections using an aberration-probe-corrected STEM instrument (Themis Z G3, Thermo Fisher) using a HAADF detector at low-electron-current settings, and a Super-X EDS analysis system (Thermo Fisher).

### Single Nanohole and QD Optical Spectroscopy Setup

The Photoluminescence (PL) of nanoholes with single QDs is measured in an inverted microscope setup (Nikon ECLIPSE Ti) in the epi-luminescence configuration described in previous works^55,56^ which illuminates the nanohole bottom through the glass substrate, simultaneously measuring the PL spectrum on an EMCCD camera, the PL lifetime, and antibunching on a pair of Avalanche photodiodes (APDs, Excelitas SPCM-AQRH-14) in the Hanbury Brown–Twiss setup,^87^ using a multichannel Time Tagger 20 (Swabian Instruments). Prior to the single nanohole measurement, a wide-field PL image of a 140 × 140 μm^2^ region of interest (ROI) is first taken by a color camera (Thorlabs CS126CU) using a 370 nm fiber-coupled wide-field LED (Prizmatix) for excitation, and then single QDs are excited and optically measured with a diffraction-limited location precision within this ROI. Using a motorized filter wheel (Thorlabs FW212CNEB) loaded with neutral density (ND) filters in the excitation laser optical path, a sequence of PL measurements with laser excitation powers ranging from 0.3 to 760 fJ per laser pulse are done for each nanohole, using a circularly polarized pulsed picosecond diode laser at 405 nm and also (for the results discussed in Figure 5) 450 and 515 nm pulsed lasers of the same series (EPL405, EPL450, EPL515, Edinburgh instruments) and excitation power to measure wavelength-dependent PL. All three lasers used have a similar excitation spot size when focused at the QD emission plane (∼0.5 μm FWHM measured from the laser back reflection on the color CMOS camera). The PL in-pulse decay time is fitted to a triexponent model, accounting for the different charging events.^88^ All of the data for the time-dependent spectrum, photon time traces, fluorescence lifetime, and second-order photon correlation are taken and analyzed from the time-tagged, time-resolved, and laser-power-resolved data by using a home-written MATLAB (2025b) code.

### SPAD-λ Optical Setup

The measurements shown in Figure 2 were performed in the same inverted microscope configuration and oil-immersion objective (×100 with a numerical aperture of 1.4, Nikon Achromat) using the 405 nm pulsed laser. Using an additional microscope output, the PL signal was focused to the slit of a spectrograph (Acton SP-2150i, Princeton Instruments) with a telescope (AC254–300-A, AC254–100-AB, Thorlabs). The light was diffracted by a grating in the spectrograph (300 grooves mm^–1^, blazed at 500 nm) and then detected by an APD array (SPAD-λ, PI Imaging). The APD array was triggered by the laser, using a constant fraction discriminator (FLIM LABORATORIES) to turn the laser’s NIM output to TTL.

### Numerical FDTD Simulations

Full-wave finite-difference time-domain (FDTD) simulations were performed using commercial software (FDTD Solutions, Lumerical Inc.). All of the simulations were performed with a mesh size of 1 nm in a rectangle larger by 100 nm than the nanohole dimensions, and 4 nm in the rest of the simulation setup region (700 nm *X* and *Y* span, 400 nm *Z* span), with perfectly matched layer (PML) boundaries, and conformal meshing to address the issue of the usage of different materials. For the far-field radiation enhancement simulation, a dipole source was used with an emission spectrum between 350 and 750 nm and averaging 3 simulations of a *Z*/*X*/*Y* oriented dipole to account for the randomly oriented exciton dipole in the QD, as shown in previous works.^29,89^ By varying the dipole position in a nanohole, the nanohole radius, and material, the average enhancement of the QD radiative rate is calculated for transitions from the band edge (645 nm) to high-energy MX states, and the resulting response spectrum is shown in the main text figures, normalized by the QD on glass substrate case. The scattering spectrum and excitation *E⃗* field enhancement inside the nanohole are simulated by a total-field scattered-field (TFSF) source of the same wavelength range, with the scattered source power measured outside the source boundaries. The relative E-field magnitude measured at *Z* = 0 nm *XY* plane and *Y* = 0 *XZ* plane, and the transmitted power out of the nanohole are simulated by a plane-wave source of the same wavelength range. More details about the FDTD simulations geometry are given in supporting Figure S15. We note that the simulated dipole enhancement values in the gold nanoholes are significantly higher than those measured. We associate the lower magnitude of enhancement with higher ohmic loss in the Au thin film due to the polycrystallinity and grain boundaries of the evaporated metal layer.^90^

## Supplementary Material



## Data Availability

The data that support the findings of this study are available from the corresponding author upon reasonable request.

## Notes

Details of the MX PL spectral and lifetime fitting procedures; supplementary PL measurements of single- and multi-QD-occupied nanoholes; statistical analysis of PL peak energies, MX PL intensity, *g* ^2^(0), and amplitude-weighted lifetimes; finite-difference time-domain (FDTD) simulations; QD ensemble PL and absorption spectra, and size distribution analysis; as well as supplementary comparison measurements of QDs coupled to Au/Al_2_O_3_ and tungsten nanoholes (PDF)
