# Supplementary material for: Plasmon-Induced Hot-State Multiexciton Emission from Quantum Dots Coupled to Metallic Nanocavities
Source: ACS Nano. 2026 May 26;20(27):19241–55. doi: 10.1021/acsnano.6c01353 (PMC13374494; doi:10.1021/acsnano.6c01353)
Supplement: Supplementary file 1 [file nn6c01353_si_001.pdf]

Supporting Information

Plasmon-Induced Hot-State Multiexciton Emission from Quantum Dots coupled to Metallic Nanocavities

Yonatan Ossia[1-3], Nadav Chefetz[1,3], Adar Levi[1,3], Einav Scharf[1,3], Oren Goldberg[2,3], Sergei Remennik[3], Atzmon Vakahi[3], Uriel Levy[2,3*] & Uri Banin[1,3*]

1. Institute of Chemistry, The Hebrew University of Jerusalem, Jerusalem 91904, Israel

2. Institute of Applied Physics, The Hebrew University of Jerusalem, Jerusalem 91904, Israel

3. The Center for Nanoscience and Nanotechnology, The Hebrew University of Jerusalem, Jerusalem 91904, Israel

* Corresponding authors: Prof. Uri Banin. uri.banin@mail.huji.ac.il,

Prof. Uriel Levy. ulevy@mail.huji.ac.il

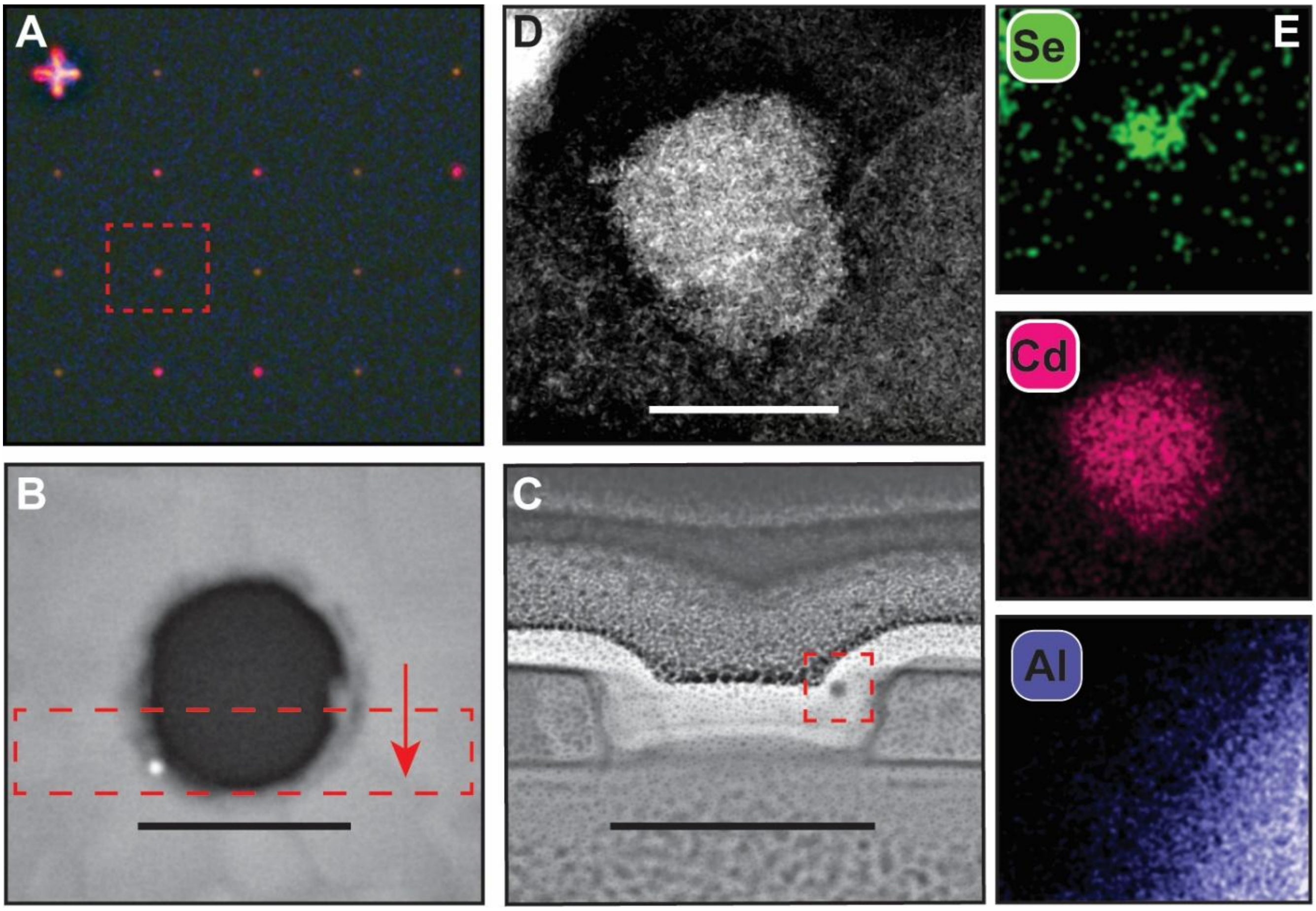

Supporting figure S1: Single Quantum Dot in Aluminum nanohole. (A) Widefield photoluminescence (PL) image of a nanohole array in a 70 nm thick Aluminum film. Nanoholes are spaced 5 microns apart to prevent coupling between close by holes. Bright Red dots are holes with QDs inside. (B) A scanning electron microscope (SEM) image of the 200 nm (black scale bar) diameter nanohole in the red dashed rectangle in panel A, showing a single QD (bright dot) coupled to the top part of the nanohole sidewall. A cross section of this nanohole is imaged in a Transmission electron microscope by High angle Annular Dark field (HAADF) scanning transmission electron microscopy (STEM), showing the nanohole in the Aluminum film (dark ~4 nm frame of the Al layer is the native Al_2O_3 oxidized layer. The single QD is seen in dark contrast on the Al-nanohole sidewall in closeup (D). (E) Energy dispersive spectroscopy (EDS) elemental mapping of the QD confirms its CdSe/CdS core/shell composition.

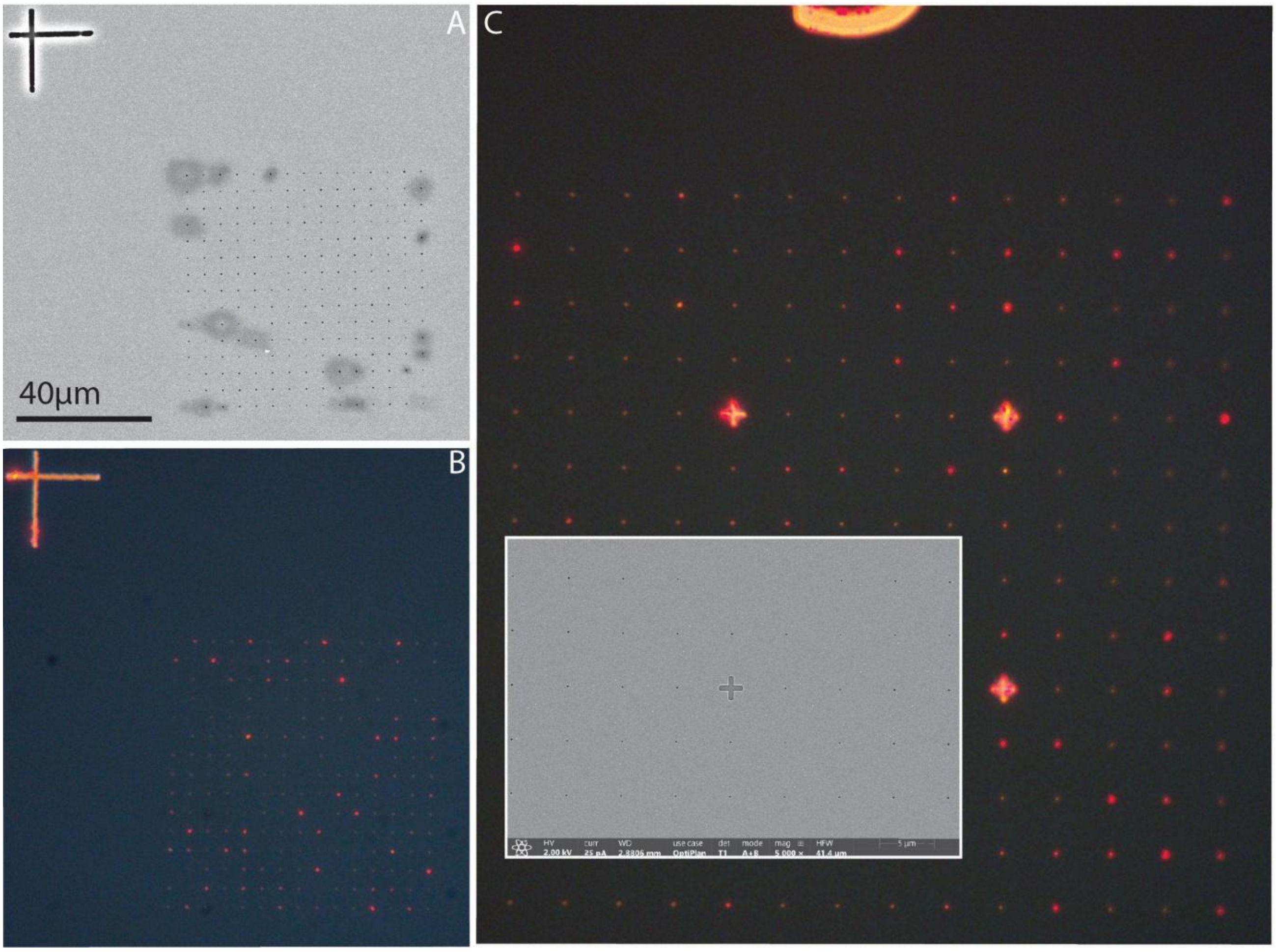

Supporting figure S2: widefield SEM and PL images of nanohole arrays: (A) SEM image of FIB milled nanohole array and (B) the widefield PL microscope image of a nanohole array after the QD drop casting procedure (red color originates from QD PL, weak light in features without QDs coming from illuminating the substrate from above with a white light LED). (C) widefield PL image of nanohole array made by the E-beam lithography and lift off process. Inset shows the corresponding SEM image of an area in the array.

Supplementary note 1: MX saturation fit methodology:

The experimental intensity counts $I(P)$, where P is the laser pulse power, are fitted to the MX intensity saturation equation:

$$I_{PL}(P) = Q \cdot \left(\sum_{N=1}^{\infty} Pois(N, \tilde{\sigma} \cdot P) \sum_{m=1}^{N} \left(1 + (m-1) \frac{k_{2X,NR}}{k_{2X,R}} \right)^{-1} \right)$$

$$+C \cdot P \left(1 - e^{-\frac{P}{p_{CW}}} \right)$$

Where $Poisspdf(N, \sigma \cdot P)$ is the poissonian probability to generate N excitons with a poissonian average $\langle N \rangle = \tilde{\sigma} \cdot P$, $\tilde{\sigma}$ is the absorption cross section (with units of the inverted laser pulse power $\tilde{\sigma} = \frac{\sigma}{A \cdot \hbar\omega}$), multiplied by the relative quantum yield of the mth exciton using the assumption that the MX QY is inversely linearly dependent on the relative non-radiative to radiative rates of the lowest order multiexciton (2X): $\frac{k_{2X,NR}}{k_{2X,R}}$. At high excitation powers where the MX recombination timescale is close to the laser pulse width (100 ps) we add a linear dependency of $I_{PL}(P)$ on the laser power, based on a CW excitation model of fluorescent molecules in metallic nanohole apertures:[1]

$I_{CW}(P) = \frac{k_{em}}{k_{tot}} \frac{\tilde{\sigma} P}{1 + \frac{P}{P_{sat}}} \approx C \cdot P$ where C is a weak CW excitation constant which includes the effective absorption ($\tilde{\sigma}$) and emission-rate (k_{em}, k_{tot}, P_{sat}) terms under CW excitation.

To correctly fit the experimental $I(P)$ data measured using an avalanche photodiode (APD), we constrain the fitting parameters in a multistep process:

1. An initial bound to the $\tilde{\sigma}, Q, C$ is achieved by fitting $I(P)$ to an initial simplified function: $I(P) = Q \cdot \left(1 - e^{-\tilde{\sigma} P}\right) + C \cdot P \cdot \left(1 - e^{-\tilde{\sigma} P}\right)$, from which the residual variance of 1 standard error is taken as the bounds for $\tilde{\sigma}, Q, C$.
2. $I(P)$ is then fit to the MX intensity saturation equation, by nonlinear least-squares optimization with moderately strict convergence tolerances (1e-7). The bounds for $\tilde{\sigma}$, Q, C are taken from the initial fit, P_{CW} is constrained by between the laser power steps where the MX lifetime (lifetime of the 1st photon in 2 photon coincidence events) is close to the APD IRF (0.7 ns) and the previous lower step. This typically gives constraints of between 100-300 fJ in nanoholes and between 250-750 for QDs on glass.

3. $I(P)$ s then fitted to the MX intensity saturation equation again in two steps: first, by constraining $\tilde{\sigma}, \frac{k_{nr}}{kr}$, and P_{CW}, then, with these parameters fixed, fitting Q and C. This approach minimizes potential bias in the fitted variables arising from the multivariable fitting process.

An additional validation of the physical basis of our model is its consistency with previously reported results by Park et al.[2] The intensity saturation behavior of a QD on glass shown in Figure 1E closely reproduces both the trend and the ⟨N⟩ dependence observed for a 16-monolayer CdS sample in that work. Notably, both systems exhibit comparable size distributions and biexciton quantum yields, as determined from the second-order correlation function $g^2(0)$ at low excitation power.

Supplementary note 2: MX PL spectrum fit methodology:

The experimental photoluminescence (PL) spectra for all measurements were fitted over the spectral range of 1.75–2.6 eV using a five-Gaussian model:

$$f(x) = \sum_{i=1}^{5} A_i \cdot e^{-\frac{(x-d_i)^2}{2\sigma_i^2}}$$

Where A_i, d_i, and σ_i represent the amplitude, peak center, and standard deviation of each component, respectively, corresponding to distinct QD energy transitions. The peak centers d_i

were constrained based on several experimental inputs: the low-power PL peak of QDs on glass (1.92 ± 0.01 eV), our recent analysis of energy- and time-resolved multiphoton coincidence measurements using the SPAD-λ setup,[3] and the minima of the second derivative of the ensemble absorption spectrum, which indicate higher-energy transitions. Together, these approaches define the allowed ranges for the PL peak positions as $d_2 = 1.98 \pm 0.02, d_3 = 2.10 \pm 0.03, d_4 = 2.23 \pm 0.05.$ The highest-energy peak is constrained to $d_5 = \ 2.50 \pm 0.08$ due to its proximity the long pass filter edge (460nm).

The FWHM of the 1S and $1S_{CX}$ transitions is constrained by the single-particle PL linewidth at low excitation power: 100±20 meV, while the $1P_{MX}$ and higher-energy states are allowed a broader range, up to twice this width.

Fitting of the power-dependent PL spectra is initiated at the lowest excitation power, where typically only the d_1 peak (on glass) and,, in some cases, the d_2 (primarily in nanoholes) exhibit non-zero amplitudes. For subsequent power steps, the fitting procedure enforces a lower bound of 0.85 relative to the previously fitted peak amplitudes and allows peak center shifts of up to 10 meV to account for charging effects and spectral diffusion between measurements. An example of this fitting procedure for a QD on glass and a QD in a nanohole is provided in Supporting Figure S9.

SPAD-λ PL peak decay fitting:

For each 20 ps time bin, the spectral fitting is performed without constraining the relative peak amplitudes, enabling resolution of both the rise and decay dynamics of each individual PL component. The area of each fitted peak is then integrated independently to construct the PL decay curves shown in Figure 2C–D of the main text. The full rise and decay dynamics are modeled using a multi-exponential function:

$$f(t) = (A_0 - e^{-\frac{t-T_0}{\tau_{rise}}}) - (A_0 - \left(A_1 e^{-\frac{t-d}{\tau_1}} + A_2 e^{-\frac{t}{\tau_2}} + A_0 - A_1 - A_2 \right))$$

where τ_{rise} represents the PL rise time convoluted with the Gaussian excitation pulse. Fitting the experimental data yields a rise time of 60±40 ps for the high-energy transitions, while also resolving the longer rise dynamics of the 1S PL peak for both the QD aggregate on glass and the 70 nm nanohole containing three QDs. The average amplitude weighted decay time for each spectral component is calculated as:

$$\langle\tau\rangle = \frac{A_1\tau_1 + A_2\tau_2}{A_1 + A_2}.$$

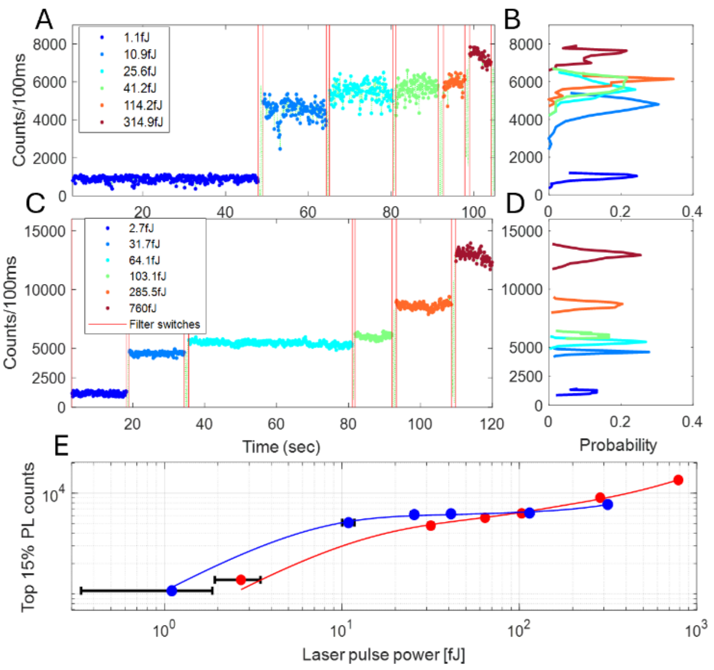

F

$$I_{PL}(P_{Laser}) = Q\cdot\left(\sum_{N=1}^{\infty} Pois(N,\tilde{\sigma}\cdot P_{Laser})\sum_{m=1}^{N}\left(1+(m-1)\frac{k_{2X,NR}}{k_{2X,R}}\right)^{-1}\right) + C\cdot P_{Laser}\left(1-e^{-\frac{P_{Laser}}{p_{CW}}}\right)$$

Fit parameter	QD-Glass	QD-nanohole
$Q\ [counts]$	5650 ± 70	3600 ± 50
$\tilde{\sigma}^{-1}$ [fJ per pulse]	4.8 ± 0.4	8.5 ± 1
$\frac{k_{2X,NR}}{k_{2X,R}}$	33 ± 10	6.2 ± 0.7
$C\ \left[\frac{counts}{fJ}\right]$	6.1 ± 0.4	9.5 ± 0.4
p_{CW} [fJ per pulse]	220 ± 50	150 ± 60

Supporting Figure S3: power dependent emission of single QDs on glass and in a 110nm nanohole. Time resolved PL counts of single QDs on a glass substrate (A) and coupled to the wall of a 110 nm diameter nanohole cavity (C), which are shown in figure 1 in the main text. Colors represent different laser excitation powers (calculated as energy per laser pulse, out of the microscope slide power meter), and the time bins are 100 ms. Probability histograms (B,D) show a broader PL intensity distribution for the QDs on glass at low power excitation, related to a larger amount of PL blinking events.[4] (E) The top 15% PL intensity bins for the PL measurements (circles, A-blue, C-red) at each laser power are plotted versus the laser power (error bars indicate the power error from the power meter (Thorlabs- S170C)). Colored lines are a fit for the intensity saturation function shown in (F) and in the main text as equation 2, with the fit parameters and errors of the QD on glass and in the 110nm nanohole. $\tilde{\sigma}$ represents the laser power needed to generate one exciton in the QD on average.

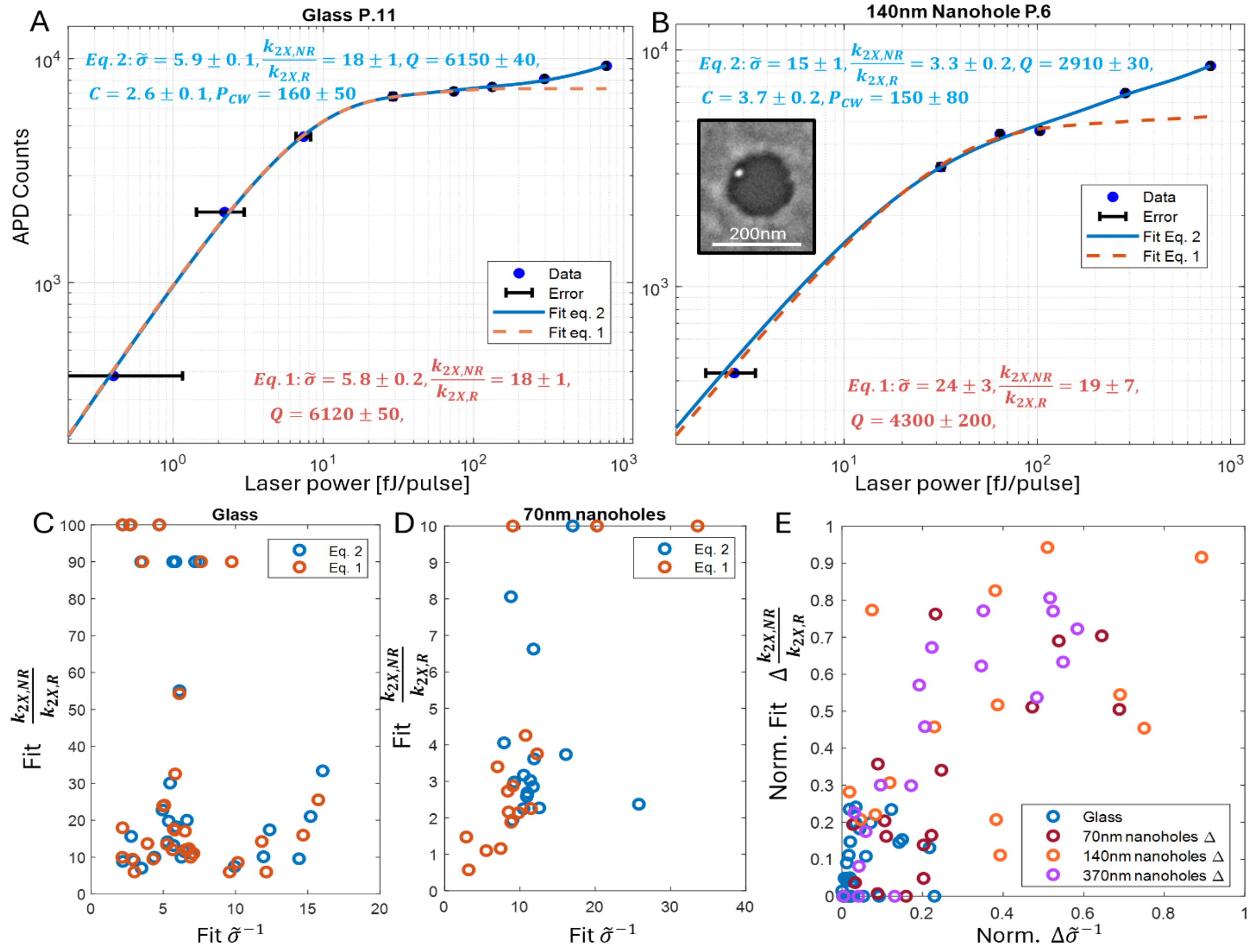

Supporting Figure S4: power dependent emission of single QDs on glass and in a 110nm nanohole. Comparison of the excitation power dependent PL intensity counts of single QDs on glass (A), and in a 140nm nanohole (B, inset shows corresponding SEM image of a single QD in this nanohole). Each PL saturation curve is fitted to both Eq.1 (orange) and Eq.2 (blue) of the main text, highlighting the differences in the saturation constants ($A = \frac{k_{2X,NR}}{k_{2X,R}}$) in the case of the nanohole. A statistical comparison of the fitted $\tilde{\sigma}^{-1}$ and $\frac{k_{2X,NR}}{k_{2X,R}}$ of for eq.1 and eq. 2, is shown for 33 single QDs on glass (C), and in 16 70nm nanoholes (D), showing distinguishable differences for the small nanoholes. (E) The normalized difference between the equation parameters is defined as: $Norm.\Delta\tilde{\sigma}^{-1} = \frac{\left|\widetilde{\sigma_2}^{-1} - \widetilde{\sigma_1}^{-1}\right|}{\widetilde{\sigma_2}^{-1}}$ evaluated for nanoholes of varying diameters and for QDs on glass. This analysis shows that smaller nanoholes exhibit a larger deviation between the two equation formulations, and establishes the validity of the modified eq.2 to represent the combined effect of pulsed and quasi-CW excitation within the nanohole cavities. W note that fitted $\frac{k_{2X,NR}}{k_{2X,R}}$ values above 30 show a high error range due to already being in the $QY_{MX} \ll 1$ regime, for this reason we limit the range of A in the fitting process up to 100.

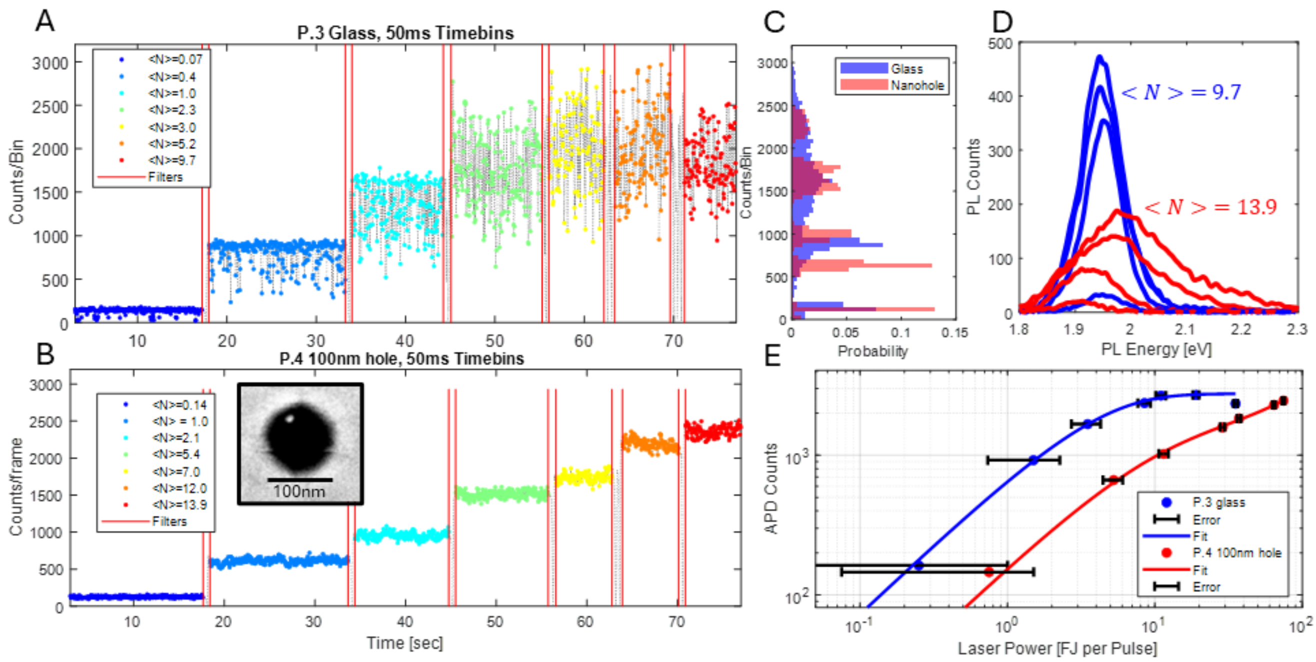

Supporting Figure S5: Enhanced photostability and suppressed PL blinking in nanoholes. (A) Power-dependent PL time trace (50ms time-bins), of a single QD on glass and (B) a single QD in a 100 nm nanohole in an aluminum film. The QDs shown here have a thinner CdS shell (10 monolayers) than those used in the main text (16 monolayers), and therefore exhibit more pronounced blinking due to a lower quantum yield of charged states and stronger coupling to surface traps. Each color corresponds to a different excitation power, with the legend indicating the extracted ⟨N⟩ values obtained from the saturation model fit in (E), highlighting the PL bleaching and intensity reduction observed for the QD on glass. (C) Histograms of PL counts per bin show a broader intensity distribution for each ⟨N⟩ in the QD on glass (blue) compared to the QD in the nanohole (red). (D) The PL spectrum of the nanohole exhibits a clear blue shift and enhanced emission from higher multiexciton states relative to the QD on glass (⟨N⟩ corresponds to the maximum excitation level in the measurement).

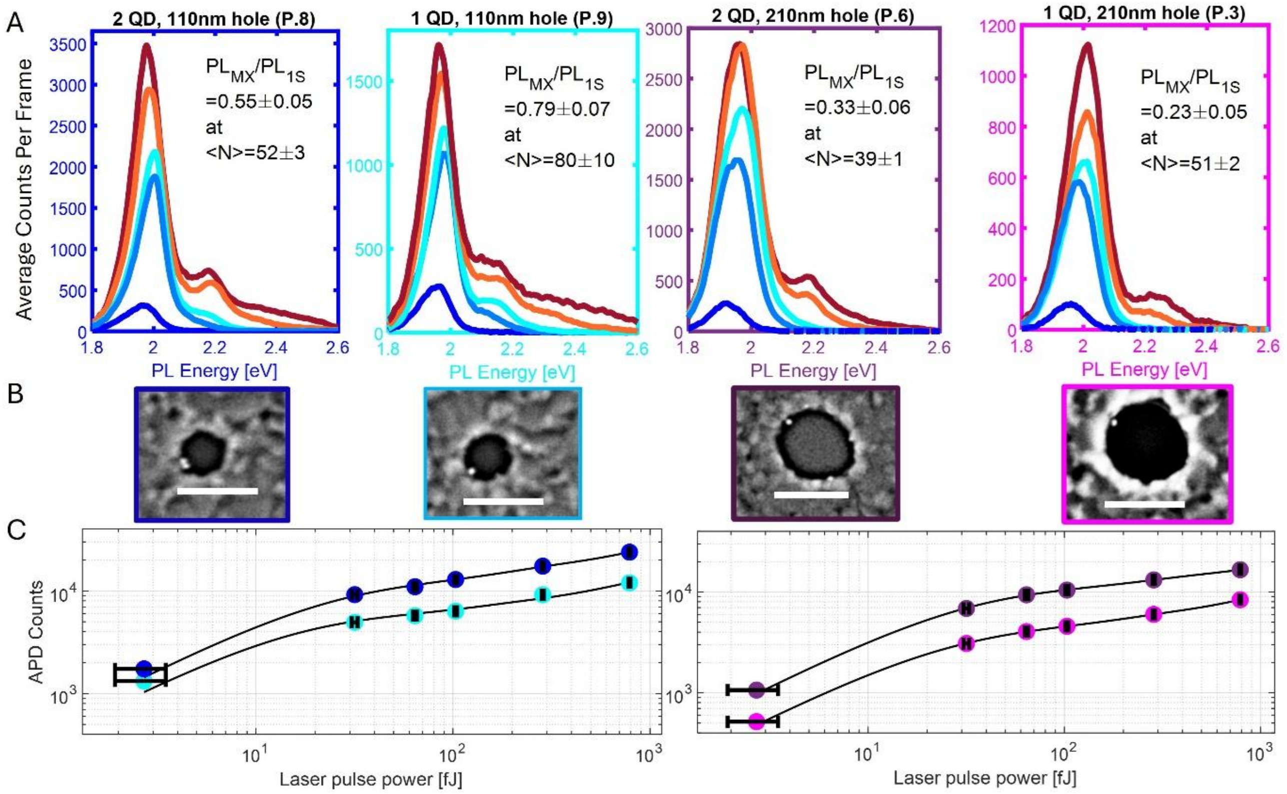

Supporting Figure S6: Power dependent emission of single and dual QD occupied nanoholes. (A) Example PL spectra of nanoholes of different diameters containing either 1 or 2 QDs coupled to the nanohole walls. The colors of the PL spectra indicate the laser pulse excitation power (2.7-787 fJ, colors indicate excitation powers as in figure S1-C). PL_{MX}/PL_{1S} is the ratio between the gaussian fitted PL peaks of the $1P_e$-$1P_{3/2}$ and higher energy MX transitions to the 1S band transitions: $\frac{PL_{high\,MX}}{PL_{1S_e-1S_{3/2}}}$ at the 787 fJ excitation power using the 405 nm pulsed laser diode. Average $\langle N \rangle$ value calculated using the saturation function fitted values $\langle N \rangle = \sigma \cdot P_{laser}$ as in the main text. (B) SEM images of the nanoholes measured in A, clearly showing 1-2 QDs coupled to the aluminum sidewalls (bright dots roughly 16 nm in size). Scale bar is 200 nm for all images, frame colors indicate the correlated PL measurement. (C) The top 15% PL intensity bins for the PL measurements (circles) at each laser power are plotted versus the laser power as in S1, for the 110/210 nm diameter nanoholes (left/right panel) showing similar parameter behavior between cases of 1-2 QDs in same diameter nanoholes, with differences mainly in the overall PL intensity. Black lines are a fit for the intensity saturation function as in supporting figure S1.

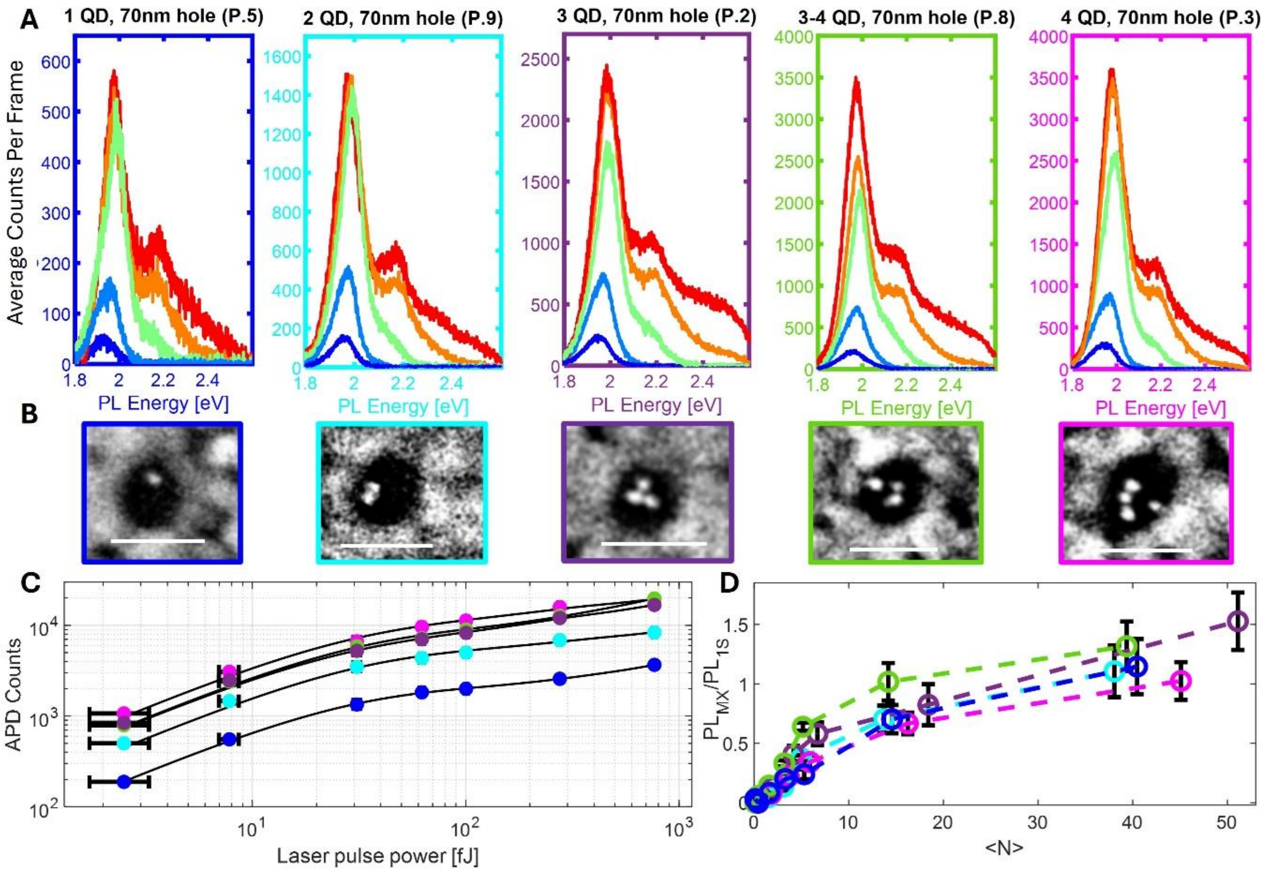

Supporting Figure S7: Power dependent emission of single to multi QD occupied 70 nm nanoholes. (A) PL spectra of QDs in 70 nm diameter nanoholes and their correlated SEM images (B). Scale bars are 100 nm. The top 15% PL intensity bins for the PL measurements (circles) at each laser power are plotted (C) versus the laser power as in supporting figure S1, showing similar behavior of the saturation σ between 1-4 coupled QDs, with differences solely in the overall PL intensity originating from the number of excited QDs. (D) The ratio of $\frac{PL_{high\,MX}}{PL_{1S_e-1S_{3/2}}}$ area for each nanohole versus the calculated pulse excited $\langle N \rangle$ value.

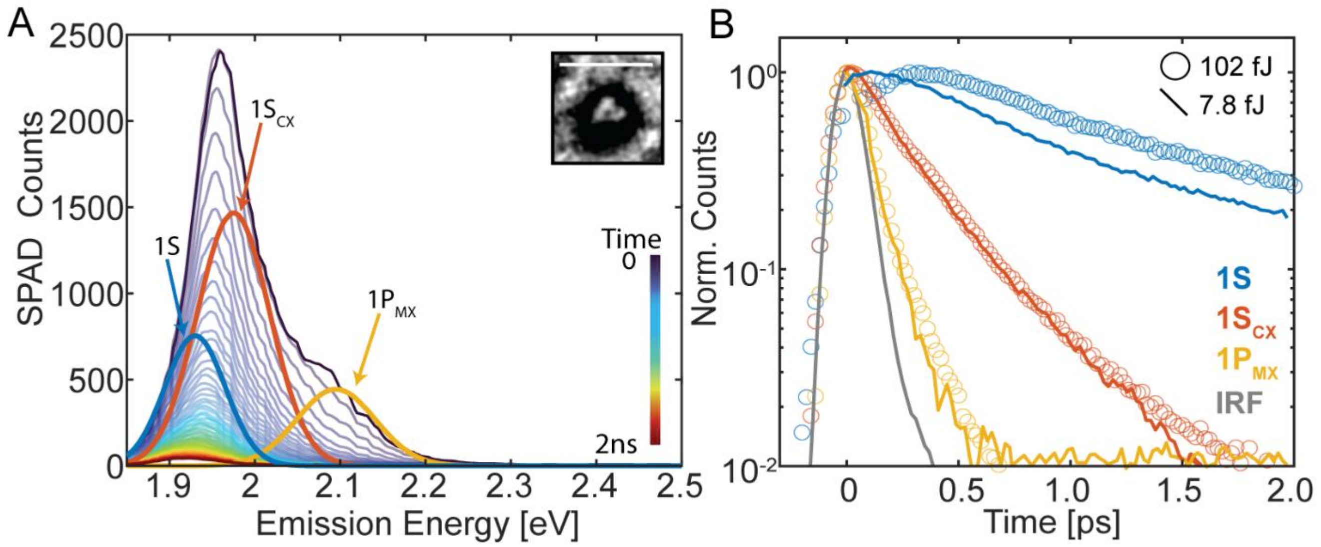

Supporting Figure S8: time and energy dependent PL of QD cluster in nanohole at low power excitation. (**A**) Time dependent PL spectra (40 ps time bins) of 3 QDs in a 70 nm diameter nanohole shown in figure 2B in the main text (SEM image in inset, scale is 100 nm), excited at 7.8 fJ per pulse power with the 405 nm picosecond laser (approximated $\langle N \rangle = 0.4 \pm 0.1$ using Supporting figure S26). Each PL spectrum is fit to a sum of 5 gaussian peaks identified as described in the main text, here showing PL emission from the $1S_e$-$1S_{3/2}$ transition of single and bi-excitons (blue), $1S_{CX}$ transition with N>3 QD exciton states (orange), $1P_e$-$1P_{3/2}$ band MX transitions (yellow). Higher energy transitions do not seem to emit at this power. (B) Time dependent decay of the distinguished PL peaks from the fits in A (colored lines), and the APD-array instrument response function (IRF-grey), compared to the time dependent decay of the same PL peak areas at high excitation power (colored circles, taken from figure 2D in the main text). Negligible differences in the decay dynamics are seen for the high energy MX states.

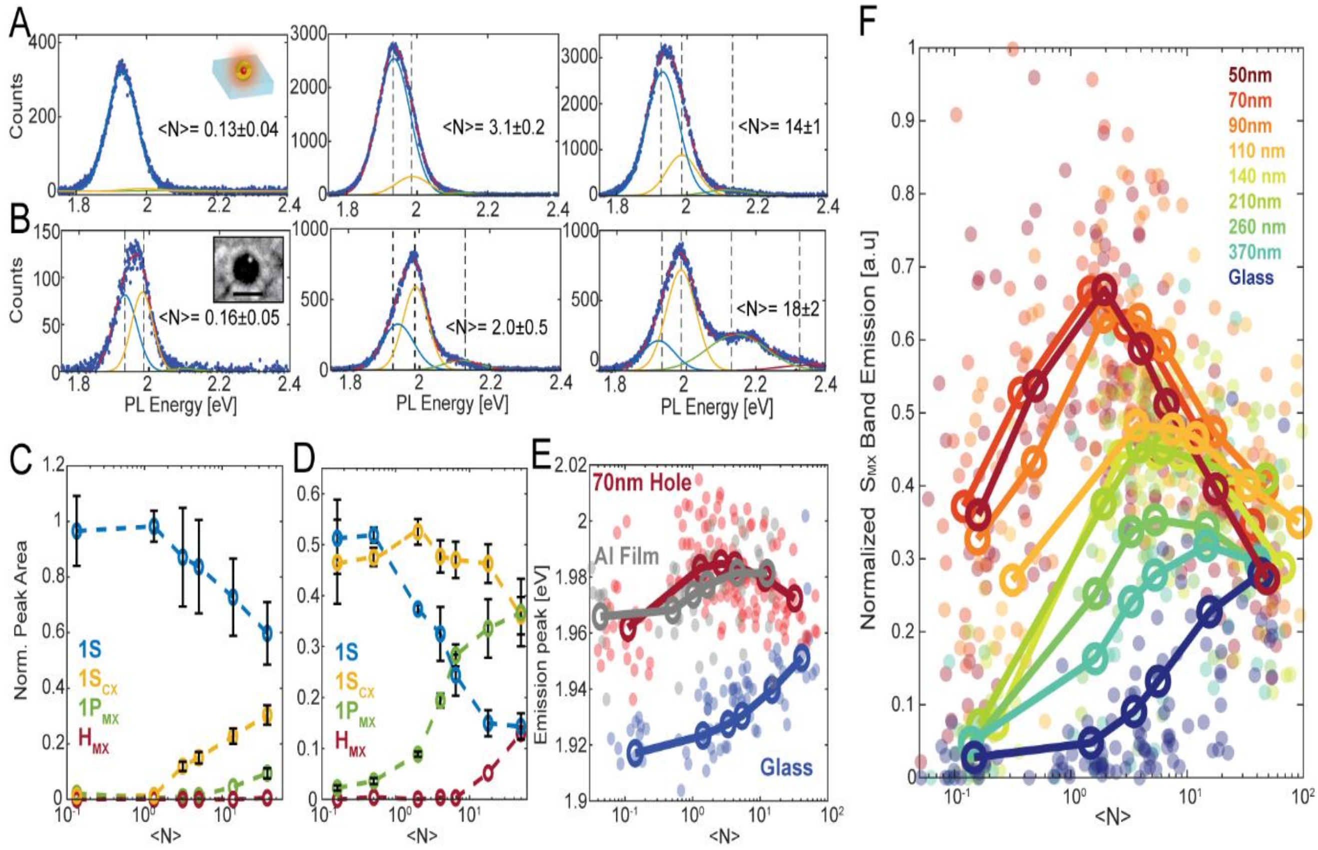

Supporting Figure S9: Spectral PL peak deconvolution and power dependency. (A-B) PL spectra of a QD on (A) glass and (B) in an Al-nanohole of 90 nm diameter (inset shows a SEM image with a scale bar of 100 nm), at similar $\langle N \rangle$ values (inset). Cyan plotted lines are gaussian peak fits to PL emission from the $1S_e$-$1S_{3/2}$ single and bi-exciton state ($1.93 \pm 0.02\ eV$), $1S_e$-$1S_{3/2}$ CX state ($1.99 \pm 0.01\ eV$), $1P_e$–$1P_{3/2}$ band MX ($2.10 \pm 0.03\ eV$), and features associated with higher-orbital MX transitions at 2.17 ± 0.03 eV and 2.30 ± 0.05 eV, respectively (dashed vertical black lines are the fitted PL peak energy centers). (C-D) the integrated PL area of the fitted energy band PL (blue-$1S_{1X/BX}$, yellow-$1S_{CX}$, green-$1P_{MX}$, red-higher MX states), versus the excited $\langle N \rangle$ values for power dependent PL measurements of on glass (C) and in the nanohole (D). (E) the total PL peak center energy versus excited $\langle N \rangle$ values of 12 QDs on glass (blue), 24 single QDs in Al-nanoholes of 70 nm diameter (red), and 10 QDs on a 20 nm Al-film (grey). Circles and thick lines are the averaged values of all QDs from each category at each excitation power. (F) the area normalized PL emission from the $1S_{CX}$ fitted PL peak, for QDs on glass and in different diameter nanoholes, versus the excitation $\langle N \rangle$ values. Circles are the average values of the PL area and $\langle N \rangle$ for each laser excitation power. For Al-nanoholes smaller than 370 nm, a saturation of the $1S_{CX}$ area is seen, with the maximal PL area value height increasing and the maximal PL area $\langle N \rangle$ value shifting to lower values, in nanoholes of decreasing diameter.

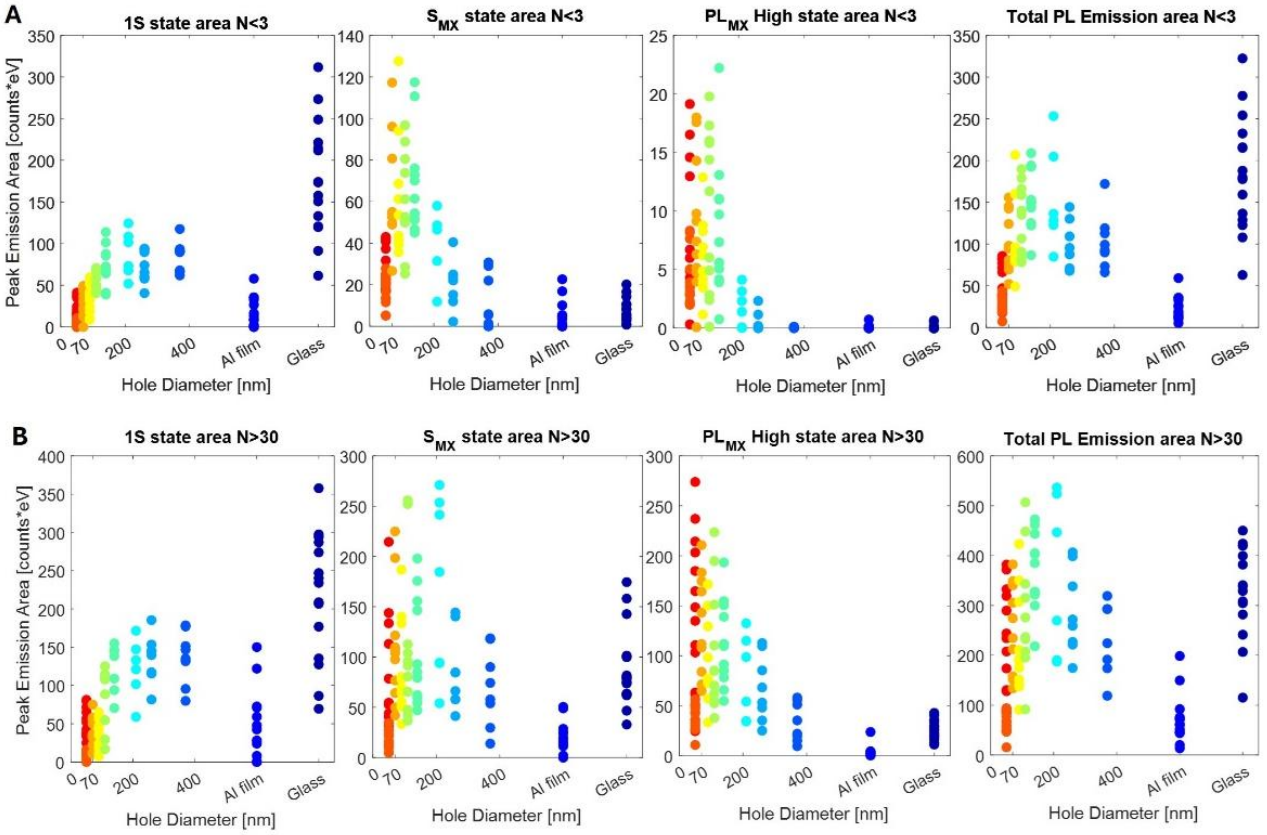

Supporting figure S10: Integrated PL of the different fitted spectral peaks of QDs in Aluminum nanoholes. The absolute PL area of each of the 5 fitted PL peaks for nanoholes of changing diameters (50 nm-370 nm), on a 20 nm Aluminum film and on glass, for (A) laser excitation power of 31 fJ per pulse and (B) 780 fJ, indicating the low and high $\langle N \rangle$ ranges. For both excitation powers the 1S state area is lower in the nanoholes than on glass, due to the reduced quantum yield of a QD near a metal surface. However, the higher energy MX PL state areas are significantly higher for the optimal sized nanoholes, and the total PL area also reaches the intrinsic values of glass at the high excitation powers, not limited by the 1X PL saturation as in the case of QDs on glass.

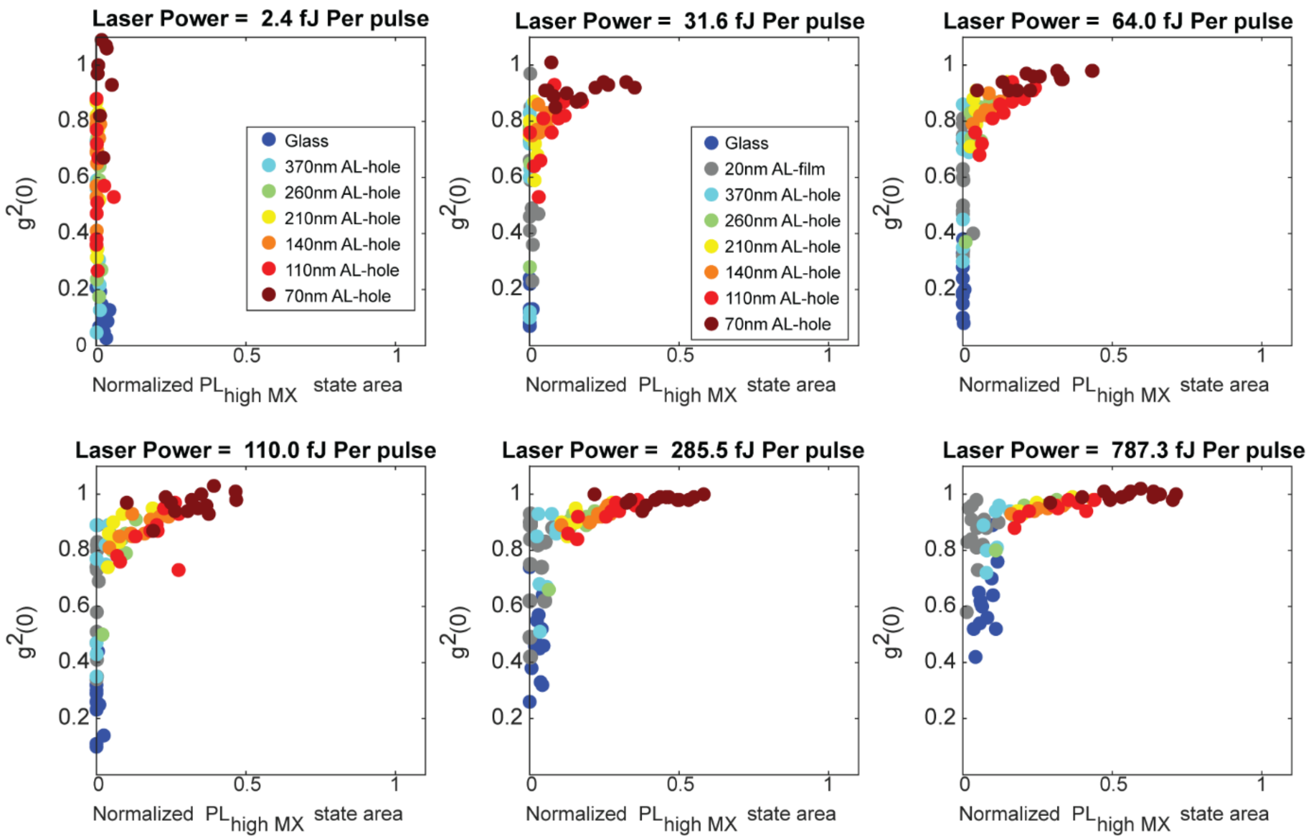

Supporting Figure S11: statistics of second order coincidences contrast ($g^2(0)$) versus high band MX PL signal for varying hole diameters and laser pulse powers. The fitted values of the $g^2(0) = \frac{G^2(0)}{G^2(next\ pulse)}$ for single QDs in Al nanoholes of different diameters (circles referring to the colors in the labels in the first two panels) and on glass (blue) and a 20 nm Al film (grey), versus the ratio of $\frac{PL_{high\ MX}}{PL_{total}}$ area for each nanohole. At the 2.4 fJ laser power, the $g^2(0)$ is already much higher than the pristine QDs on glass, indicating enhanced bi-exciton emission. With increasing the laser power, the $g^2(0)$ saturates, with each QD emitting on average more than 2 photons, with more than half the measured photons originating from energy bands above the 1S band edge.

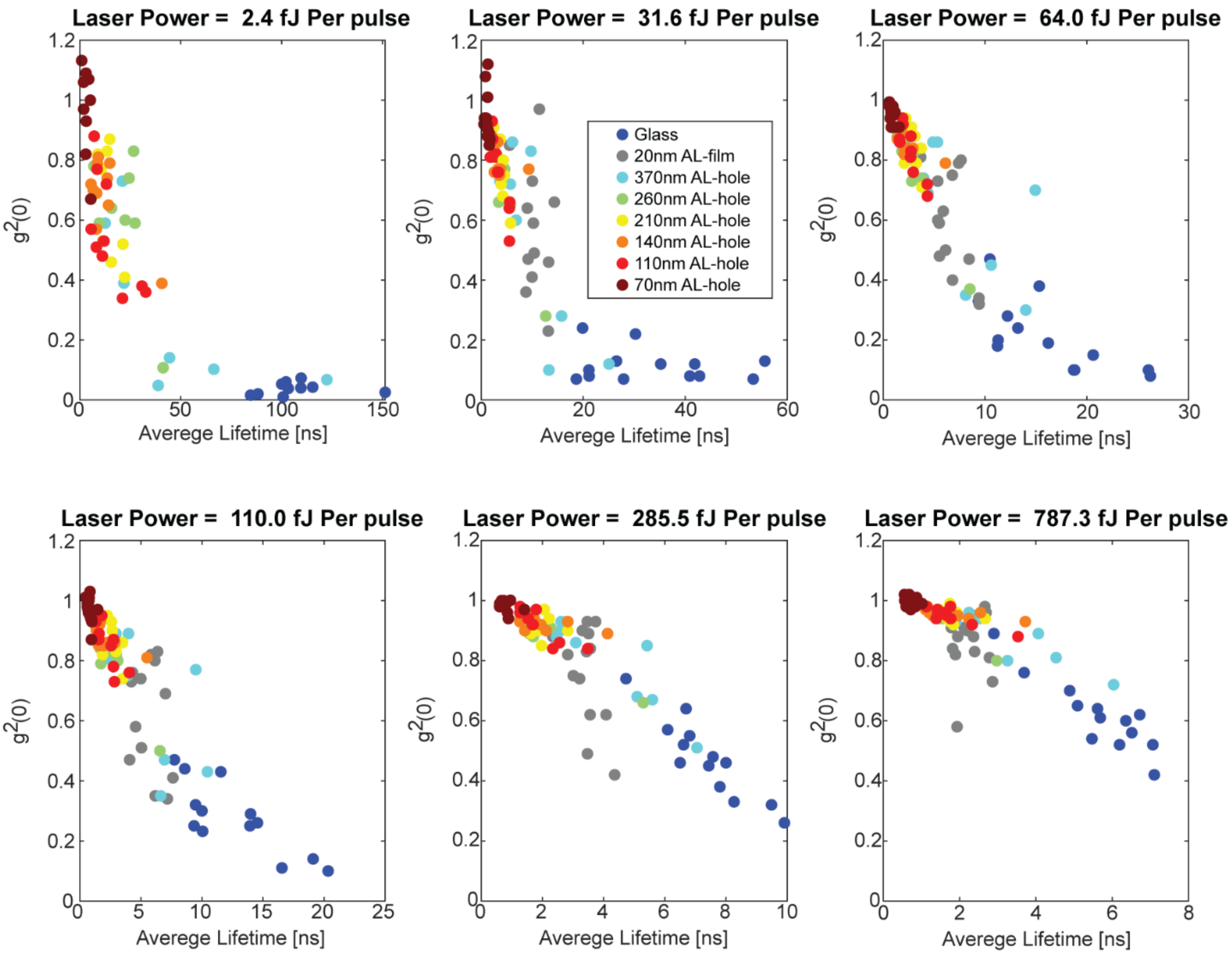

Supporting Figure S12: Statistics of second order coincidences contrast ($g^2(0)$) statistics versus PL lifetime decay, for varying hole diameters and laser pulse powers. For excitation powers above 31 fJ, the PL lifetimes for glass and Al-nanoholes above 260 nm in diameter are dominated by MX radiative and non-radiative interactions, seen by the significant lifetime shortening. Smaller diameter nanoholes show MX dominated PL lifetime at all excitation powers. At 2.4 fJ, the averaged lifetime values mean from the 10 single particle measurements on glass: $\langle \tau_{glass} \rangle = 110 \pm 20ns$ and in the 70nm nanoholes: $\langle \tau_{70nm\ AL-hole} \rangle = 3 \pm 1ns$, (lowest value $\tau = 0.9 \pm 0.1ns$), giving a statistical rate enhancement factor of $\frac{\langle \tau_{glass} \rangle}{\langle \tau_{70nm\ AL-hole} \rangle} = 37 \pm 14$.

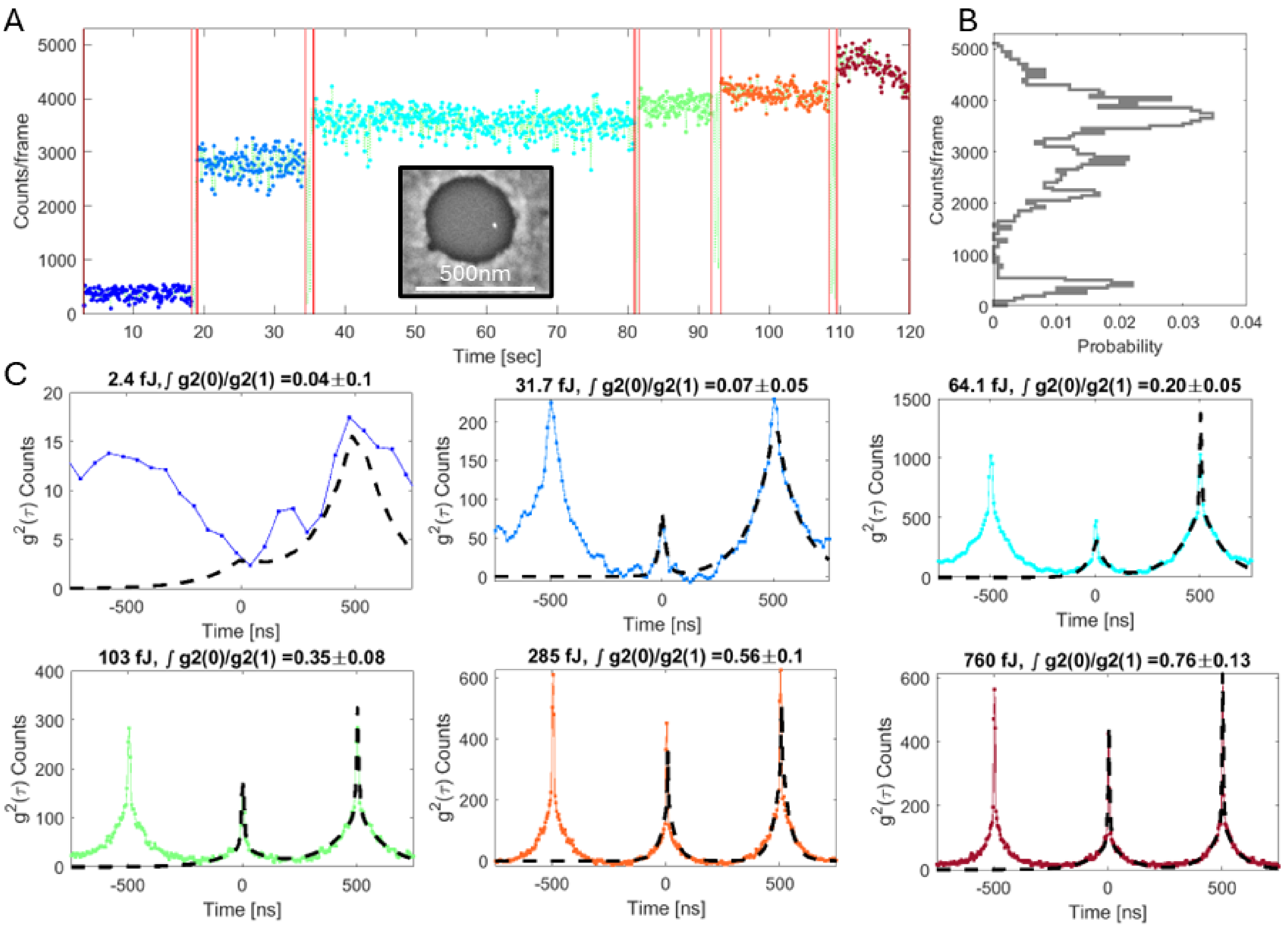

Supporting Figure S13: Power-dependent PL and second-order correlation of a single QD in a 370 nm nanohole. (A) PL time trace as a function of increasing excitation power for a single QD located at the bottom of a 370 nm nanohole (main text Figure 4, light blue circle), showing blinking behavior comparable to that of a QD on glass (Supporting Figure S3). (B) Intensity histogram (counts per bin) compiled from the full power-dependent measurement. (C) Second-order correlation function $G^2(t)$ extracted from the time traces in (A) for different excitation powers. The dashed black lines represent biexponential fits to the coincidence peaks at 0 ns delay and at 500 ns (corresponding to the next laser pulse). The extracted $g^2(0)$ defined as the ratio between the fitted peak amplitudes at 0 ns and 500 ns delay, is indicated in each panel along with the excitation power. For the lowest excitation power (2.4 fJ), where fitting is unreliable due to limited photon counts, $g^2(0)$ was estimated by numerical integration of the central peak relative to the normalized side peaks over a 500 ns window, yielding $g^2(0) = 0.04\pm0.1$

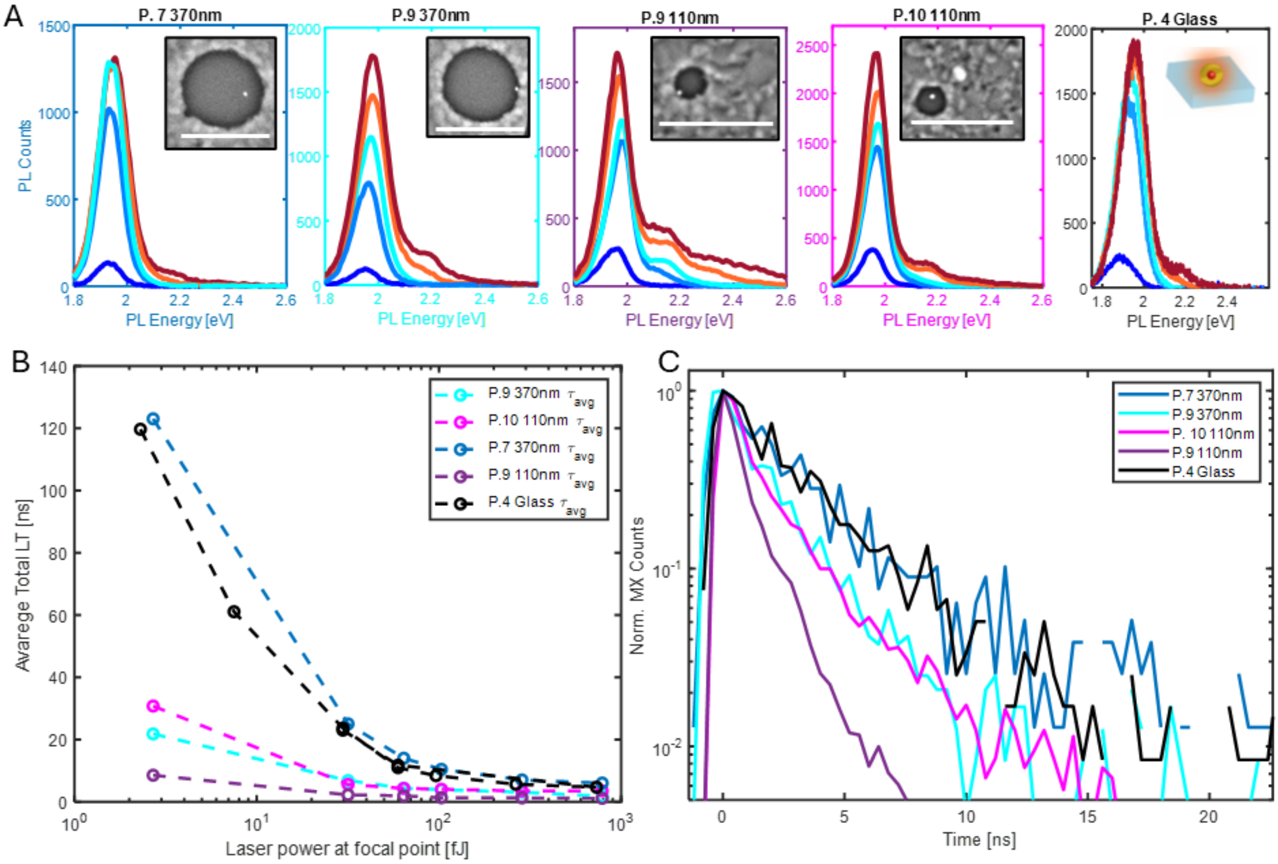

Supporting Figure S14: PL spectra and Lifetimes of single QDs in the center/side of Al-nanoholes. (A) the PL spectra of the single QDs in nanoholes shown in figure 4 in the main text, at laser excitation powers between 2.7 (blue) to 787 fJ (dark red). (B) The weighed PL lifetime (LT) decay value (3 exponential fit) $\tau_{avg} = \frac{A_1 \cdot \tau_1 + A_2 \cdot \tau_2 + A_3 \cdot \tau_3}{A_1 + A_2 + A_3}$ at each excitation power, showing the drastic lifetime shortening in the MX excitation power regime (higher than 31 fJ for all nanoholes with QDs in proximity to the Al-cavity walls). (C) The PL decay lifetime of the first photon in measured photon pair events on the 2 APDs in a Hanbury-Brown-Twiss configuration, which is the decay time of MX events (more than one emitted photon per laser pulse), at 31 fJ excitation power ($1 < \langle N \rangle < 3$). The similar MX lifetime trace between P.7 (370 nm nanohole) and P.4 (glass) combined with similar MX PL at high excitation powers, indicates the plasmonic effect in the 370 nm nanohole far from the wall is negligible.

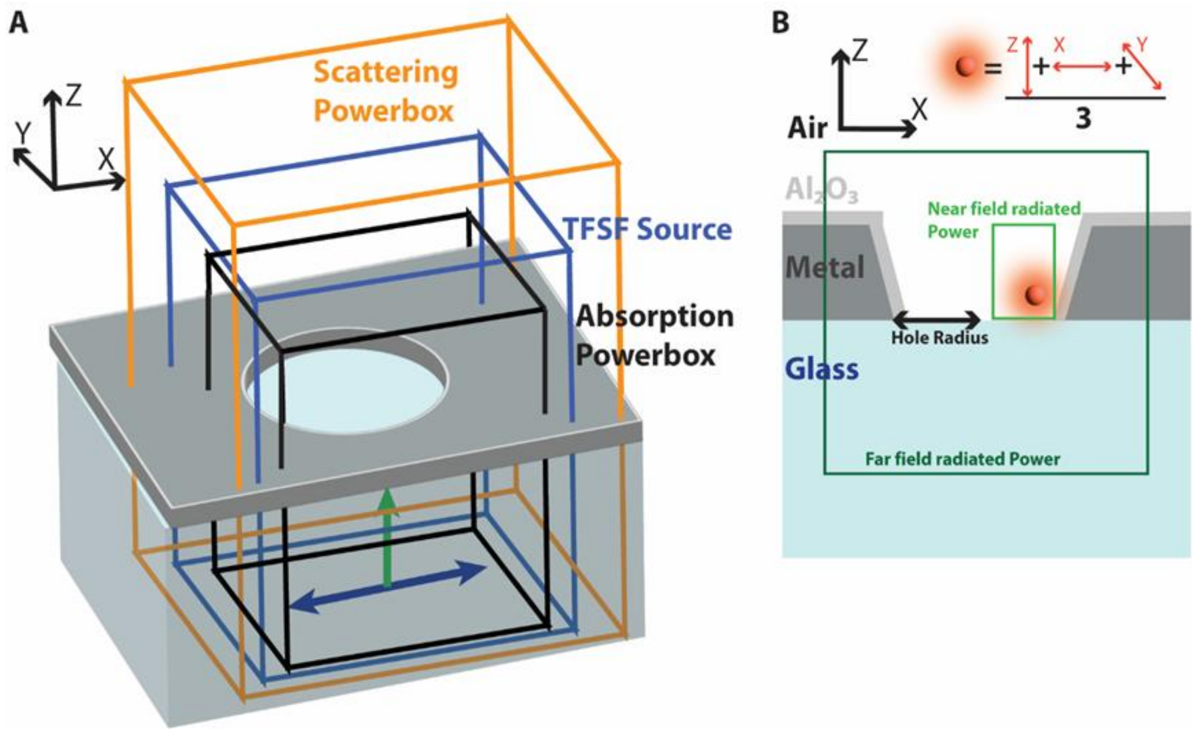

Supporting Figure S15: Finite-Difference Time-Domain simulation setup. (A) a 3D view of the nanohole structure in the glass substrate simulated in Lumerical FDTD engine. For the scattering cross section and E-field enhancement simulations, a Total-Field-Scattered-Field planewave source is used, propagating in the Z direction from the glass side, and polarized along the X axis. A monitor box measuring the power flow inside the TFSF source box is used to calculate the absorbed power by the metal structure, and an additional power flow box outside the TFSF source box is used to measure the scattered power. The scattered power normalized by the nanohole bottom cross section (πR_{hole}^2) gives the area normalized scattering cross section shown in figure S17. The E-field magnitudes in the Z=0 XY plain and Y=0 XZ plain shown in figure 4 in the main text, are measured by a power monitor in each respective plain and averaged for an X and Y polarized plane wave source, to simulate the circularly polarized laser excitation. (B) A XZ plane view of the nanohole structure, the hole radius is defined as the hole radius at Z=0 (which can be corroborated by SEM imaging), up to the Al_2O_3 coating layer. A point dipole source is positioned 8nm above the glass plain (QD center) in the hole, and the non-polarized power radiated is simulated by averaging simulations of the X/Y/Z oriented dipoles. The radiative power enhancement is calculated by measuring the power flow out of the far-field box monitor (dark green). The total dipole power enhancement is calculated by the near-field box monitor (light green). The difference between the values of these two monitors is the radiation absorbed by the metal (ohmic loss). The ratio of the power flow out of the far-field box for a radiating dipole source in the nanocavity relative to the power flow of the same dipole source on glass in equal to the radiative rate of the source in the nanocavity relative to the radiative rate on a glass substrate: $\frac{P_{nanohole}}{P_{glass}} = \frac{\gamma_{nanohole}}{\gamma_{glass}}$. [5,6] where P is the power flow out of the monitor box and γ is the rate of the transition. Due to the encasement of the QD in the nanohole, the ohmic loss and PL scattering drastically reduces the collection efficiency therefore the radiated power outside the nanohole is a truer variable for the emission enhancement of the single and multiexciton transitions.

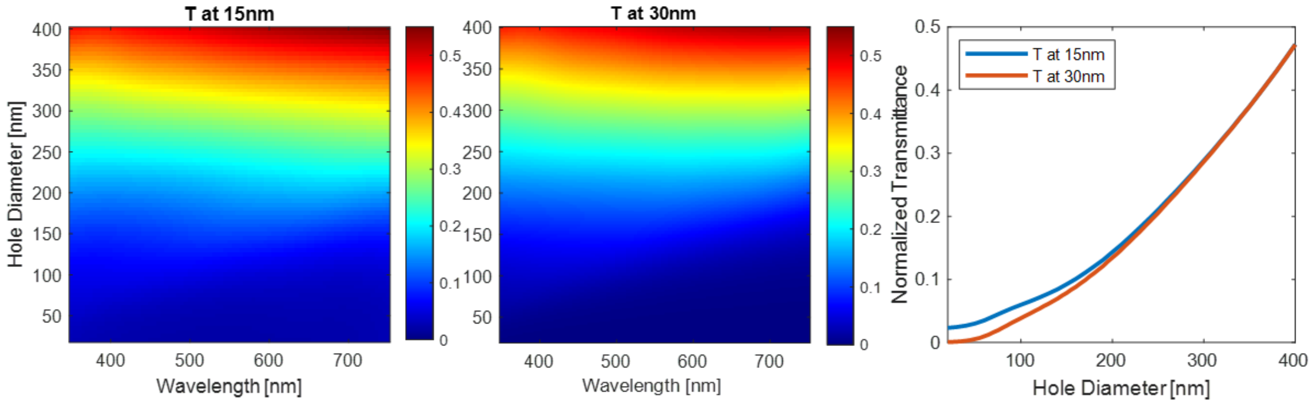

Supporting Figure S16: Plane-wave transmission through Al nanoholes.

FDTD simulations of plane-wave transmission through XY planes within a nanohole, evaluated at mid-height (15 nm above the glass substrate) and near the top (30 nm above the substrate), together with a line profile comparison at 405 nm. The transmission exhibits a broadband spectral response and shows a pronounced decrease at smaller nanohole diameters, consistent with the reduced excitation and absorption observed experimentally for the QDs located above the nanohole bottom.

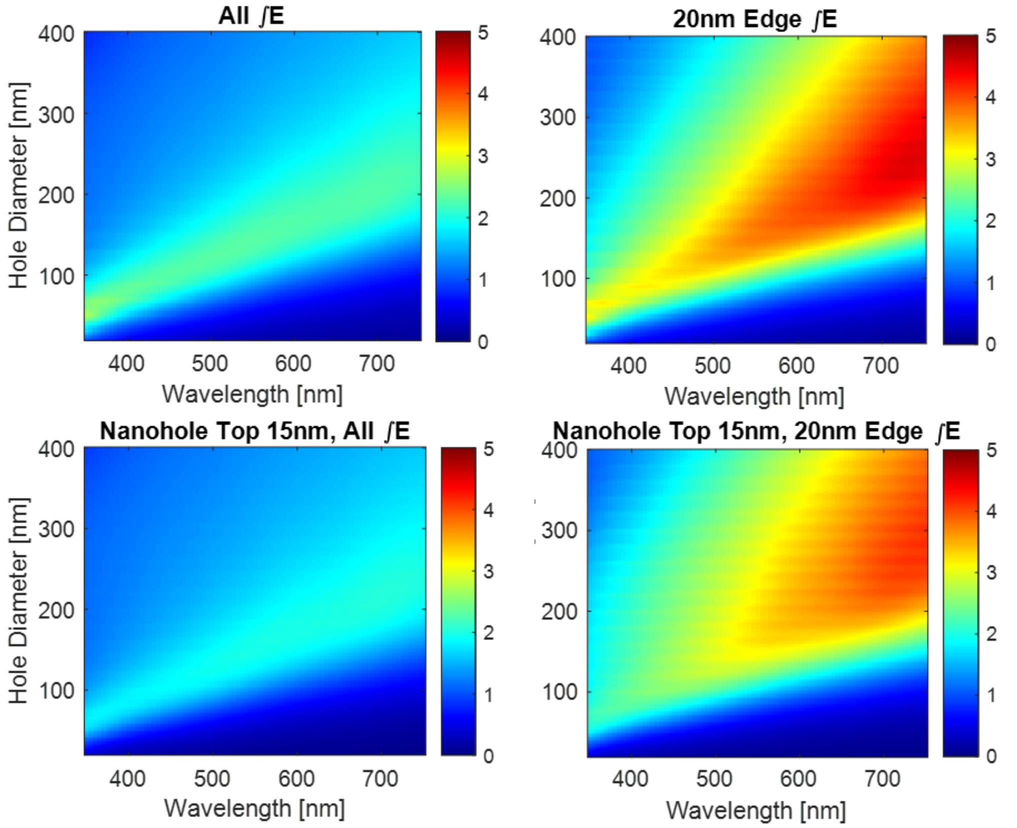

Supporting Figure S17: Near field $|E|^2$ enhancement in nanoholes TFSF source. Simulated $|E|^2$ enhancement of a circularly polarized plane-wave source, compared for the entire nanohole area and the 20nm edge area of the nanohole sidewall, normalized to the area volume: $\langle |E|^2 \rangle = \frac{\int |E(r)|^2}{\int r}$, showing a broad enhancement in the excitation wavelengths and QD emission spectral range. Notable, QDs located in the top and center of the nanohole experience lower field enhancement.

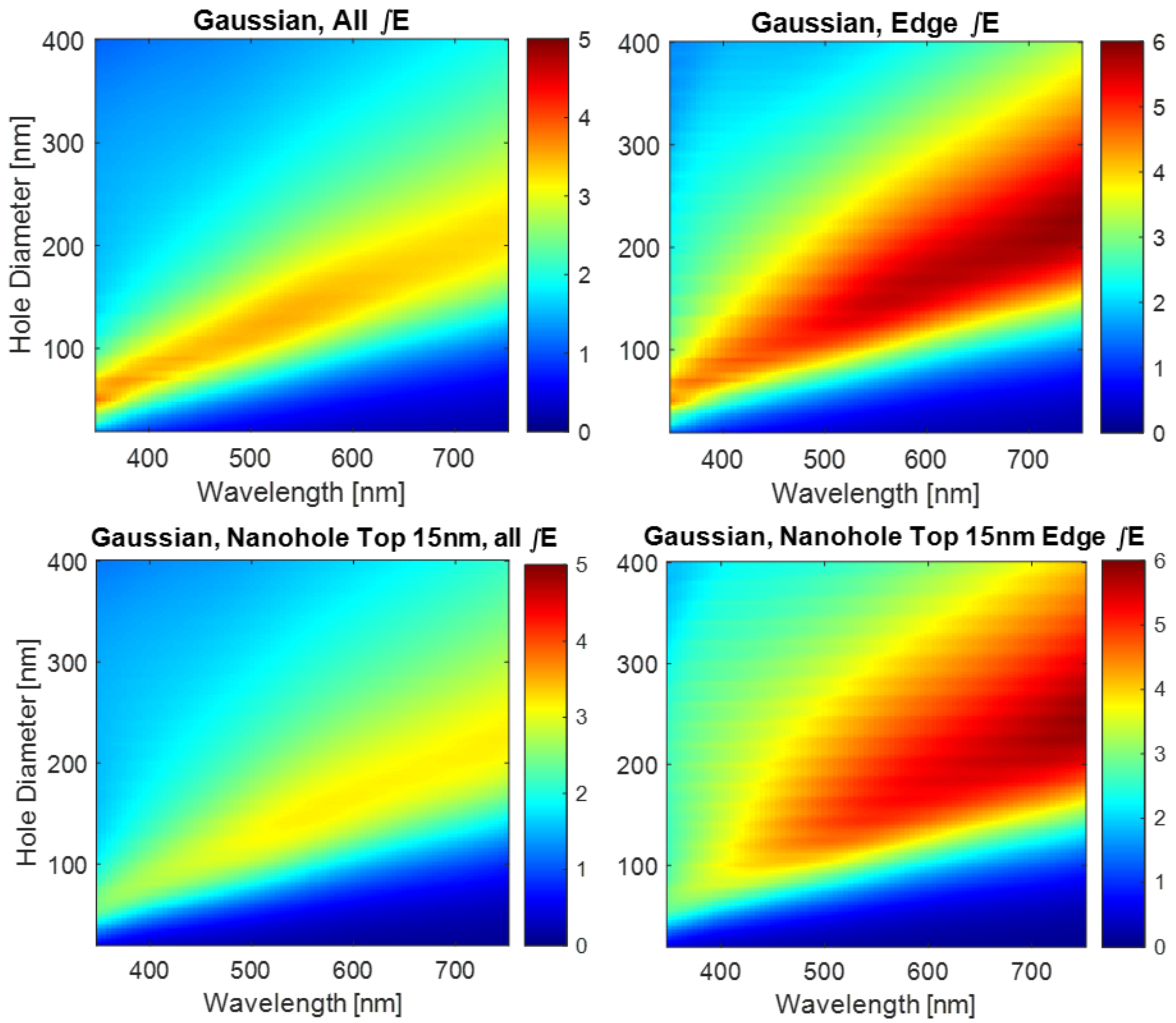

Supporting Figure S18: Near field $|E|^2$ enhancement in nanoholes gaussian source. Simulated $|E|^2$ enhancement of a circularly polarized gaussian-wave source focused through a 1.4 N.A objective, compared for the entire nanohole area and the 20nm edge area of the nanohole sidewall, normalized to the area volume: $\langle |E|^2 \rangle = \frac{\int |E(r)|^2}{\int r}$, showing a broad enhancement in the excitation wavelengths and QD emission spectral range. Enhancement values are slightly higher than for the plain wave TFSF source.

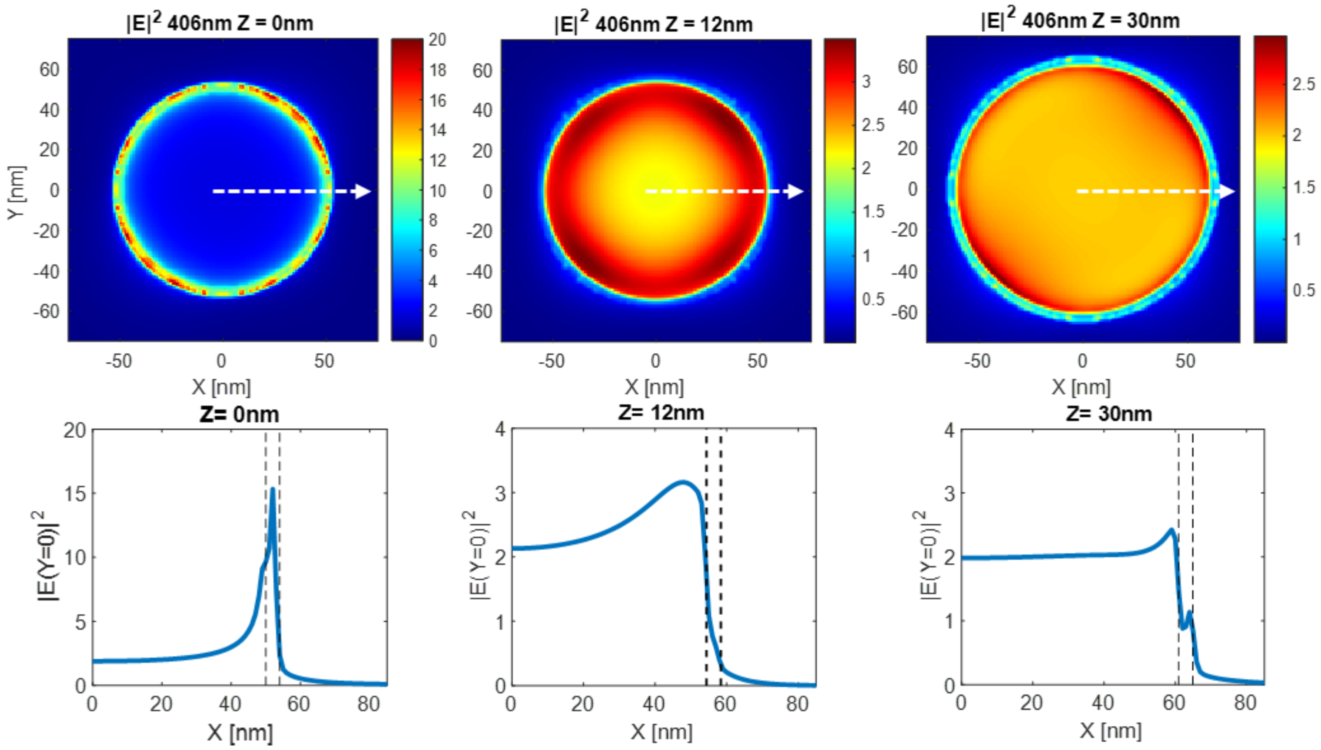

Supporting Figure S19: Near field $|E|^2$ cross section in nanoholes TFSF source. Simulated $|E|^2$ enhancement in the XY plain of a circularly polarized plane-wave source in a 100nm diameter nanohole, at different Z heights. Showing reduction and broadening of the $|E|^2$ magnitude for higher Z locations in the nanohole. Lower panel shows corresponding line plots at Y=0 for each height. Showing the field broadening.

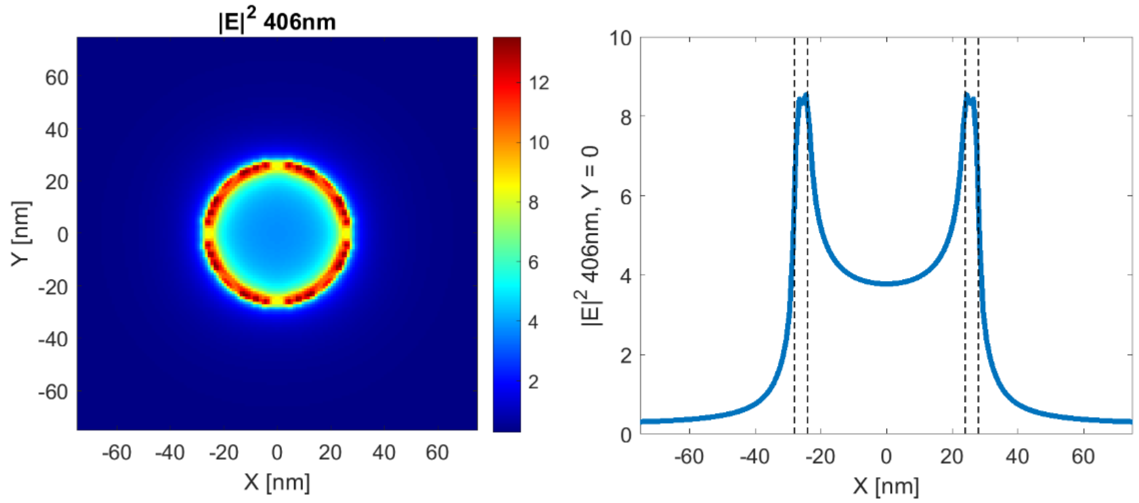

Supporting Figure S20: electric field enhancement in a 50 nm diameter Al-nanohole. XY plane simulation of $|E|^2$ of a 406 nm wavelength circularly polarized plane-wave source at the bottom of the 50 nm diameter Al-nanohole, showing higher field-density near the Al-Al_2O_3 sidewalls. Right panel is a 1D section plot at Y=0, dashed black lines show the Al_2O_3 native oxide regions

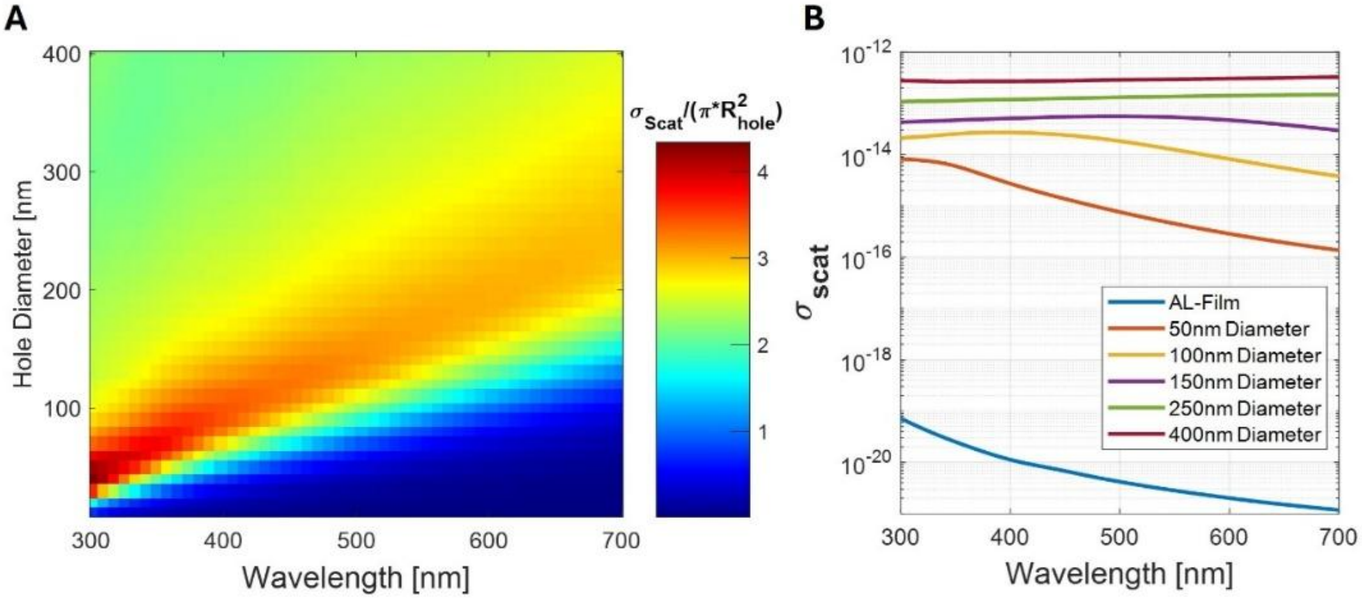

Supporting Figure S21: FDTD scattering from an AL-nanohole. (A) The normalized scattering cross section of a TFSF plain wave in a single nanohole with variable diameter (the absolute scattering cross section is normalized by the hole bottom cross section). (B) Absolute value of the scattering cross section for selected hole diameters and a 35 nm AL-film on glass, showing red-shifting and broadening of the scattering maxima with increasing nanohole diameter.

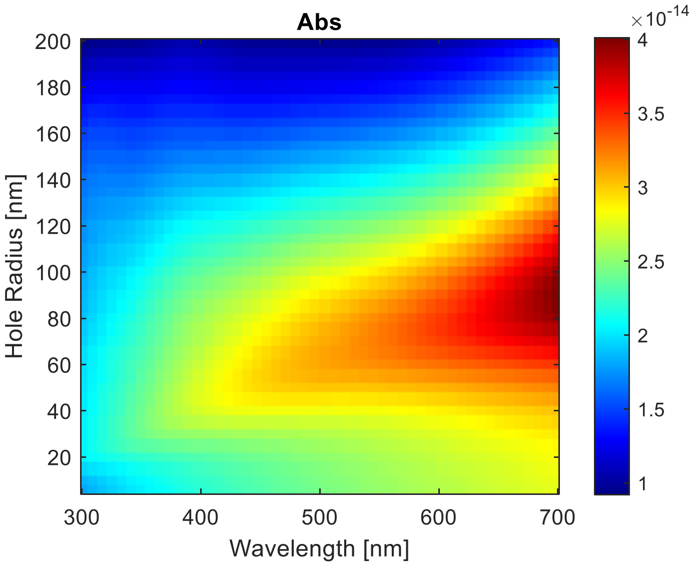

Supporting Figure S22: FDTD absorption of an AL-nanohole. simulated power absorption of a TFSF plain wave source ($0.5*0.5\mu m^2$ square source), showing a maximum for nanohole diameters of 80-240nm (lower diameters reflect more of the source light, as seen in figure S21.

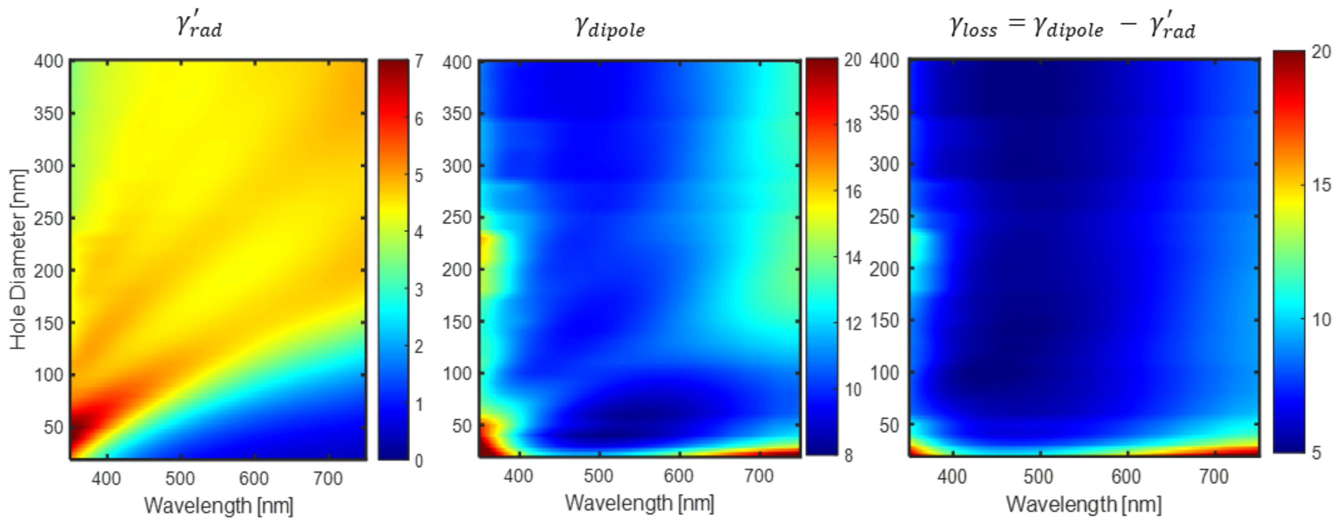

Supporting Figure S23: Simulated near-field dipole enhancement and calculated ohmic loss. FDTD simulation of a dipole emitter 8 nm from the sidewall of an Al-nanohole with varying hole diameter. The left panel shows the dipole power enhancement outside the nanohole transmission box, which is the enhancement in the radiative emission rate combined with the collection efficiency, shown also in figure 6D of the main text. The center panel shows the near field dipole power enhancement, which is the total rate enhancement of the emitter due to the cavity relative to the emission rate on glass (assuming no non-radiative recombination of the 1X and MX transitions). when generating multi-excitons, the recombination rate will be higher due to additional competitive multi-particle recombination pathways. The right panel is a calculation of the relative power absorbed by the metal cavity surroundings by the difference between the near field and far-field transmission boxes, which is the ohmic loss rate of the QD-cavity system, quantifying the metal-QD interaction.

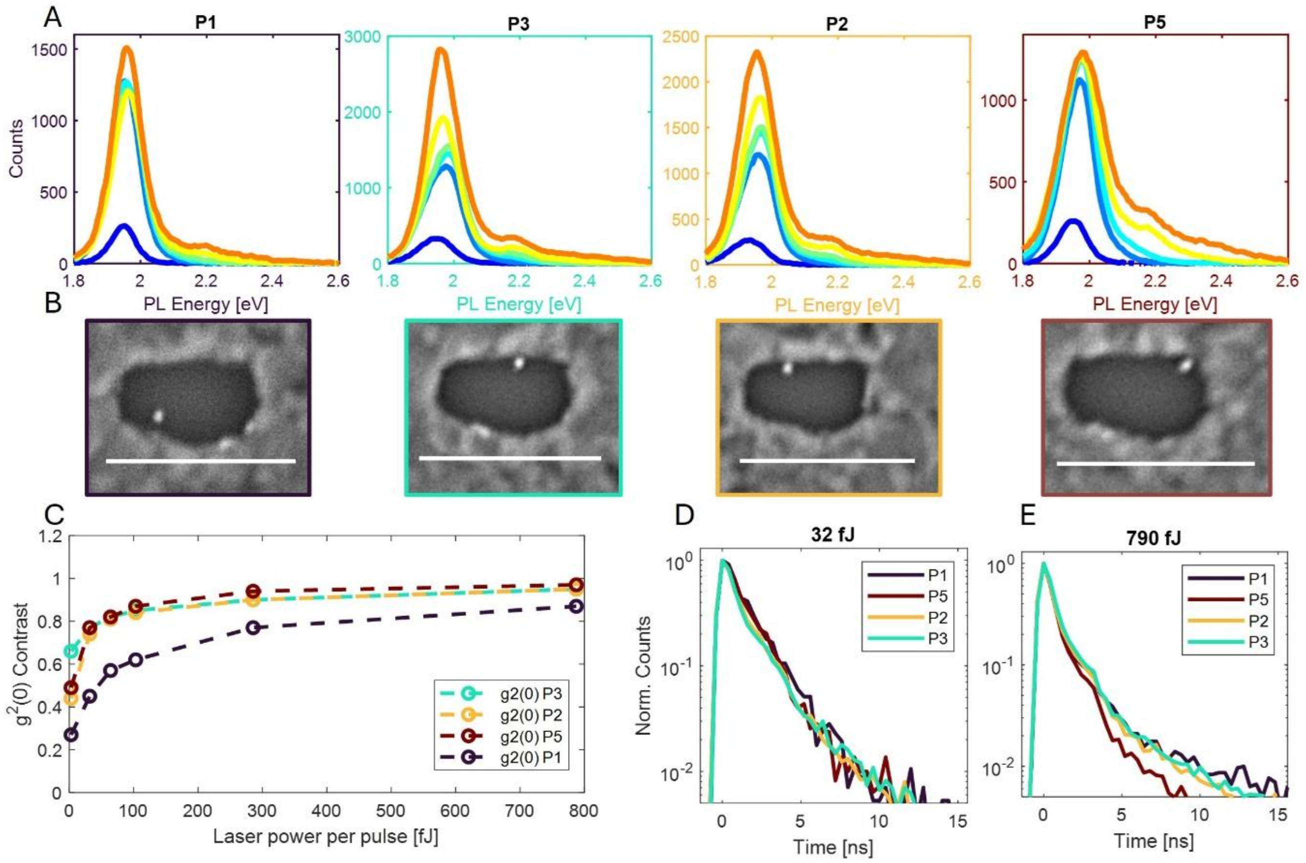

Supporting Figure S24: PL spectra of single QDs in rectangular nanoholes. (A) PL spectra and (B) corresponding SEM images of 4 rectangular nanoholes, differing solely in the position of a single QD inside them (white scale bars are 300 nm). While all the QDs adjacent to the nanohole wall show a similar $g^2(0)$ saturation (C) and PL decay at 32 fJ excitation power (D), P.5 which is located at the rectangle corner shows enhanced high energy MX emission and a shorter PL lifetime at high excitation power (E), indicating faster MX radiative recombination.

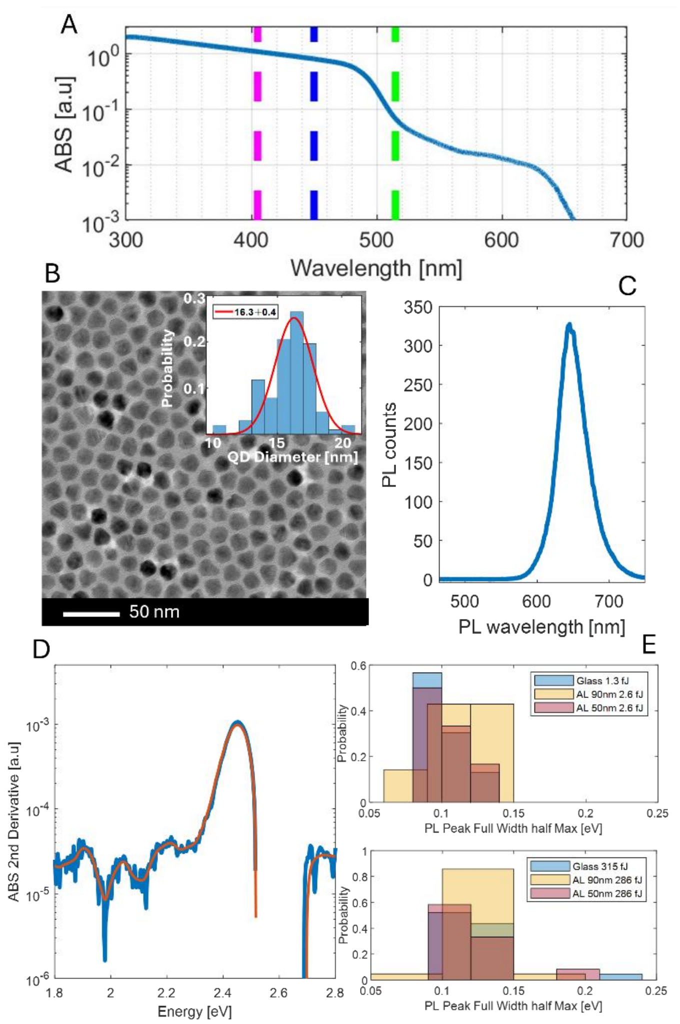

Supporting Figure S25: QD Ensemble properties. (A) The absorption of a dilute solution of QDs in Log scale, with colored dashed lines indicating pulsed laser excitation wavelengths used in the measurements in figure 5 in the main text. Above an order of magnitude difference is seen between the absorption at 515 nm to the 450 and 405 nm wavelengths. (B) Transmission electron microscope image of the CdSe/CdS core/shell QDs used in this work. The diameter statistics of 120 QDs is shown in the inset, with an average diameter of $16.3 \pm 0.4\,nm$. (C) The PL spectra of the ensemble solution, with the central emission wavelength at $646 \pm 2nm\,(1.920 \pm 0.006\,eV)$. (D) The second derivative of the QD absorption spectra (raw-blue, gaussian smoothed-orange). Showing local minima at 1.97 eV, 2.1 eV, and 2.25 eV, fitting expected higher energy MX transitions in the single particle PL spectra fitting procedure. (E) the full-width-half-max (FWHM) of the main PL peak of single QDs on glass and in Al-nanoholes, at low and high-power excitation.

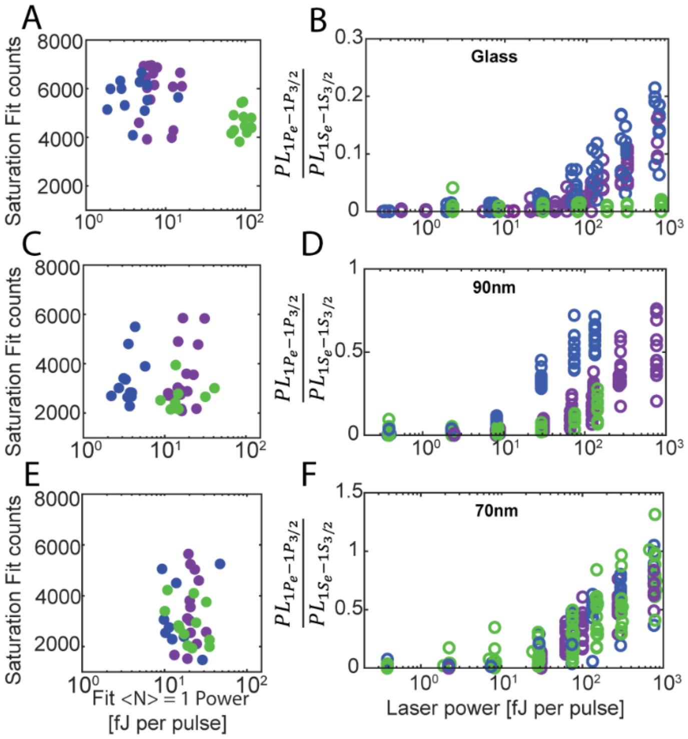

Supporting Figure S26: wavelength and power dependent saturation fit values and MX emission ratio. (A) fitted Saturation counts (Q parameter) versus laser power needed to reach C1 (equals $\tilde{\sigma}^{-1}$ from equation 2 in main text) for different QDs excited by different laser wavelengths (405 nm-purple, 450 nm-blue, 515 nm-green) on glass. (B) The $\frac{PL_{1P_e-1P_{3/2}}}{PL_{1S_e-1S_{3/2}}}$ area ratio for the QDs on glass versus laser excitation power. The higher values for the 405 nm and 450 nm excitation are linked with a higher <N> value due to the smaller $\tilde{\sigma}$ values seen in A. (C,E) Saturation counts versus $\tilde{\sigma}^{-1}$ from saturation fit, same as in panel A for QDs in 90 nm (C) and 70 nm (E) diameter Al holes. (D,F) $\frac{PL_{1P_e-1P_{3/2}}}{PL_{1S_e-1S_{3/2}}}$ area ratio

for the QDs in C, E, versus laser excitation power. From these fitted single particle results we calculate the average $\langle\tilde{\sigma}\rangle$ for the 405nm excitation laser on glass: $\langle\tilde{\sigma}_{glass}\rangle = 7 \pm 4\, fJ^{-1}$ and in the 70nm Al-nanoholes: $\langle\tilde{\sigma}_{70nm}\rangle = 20 \pm 5\, fJ^{-1}$, which are used to approximate the <N> values at the excitation powers for the QD-aggregates shown in figure 2 in the main text and Supporting figure S6.

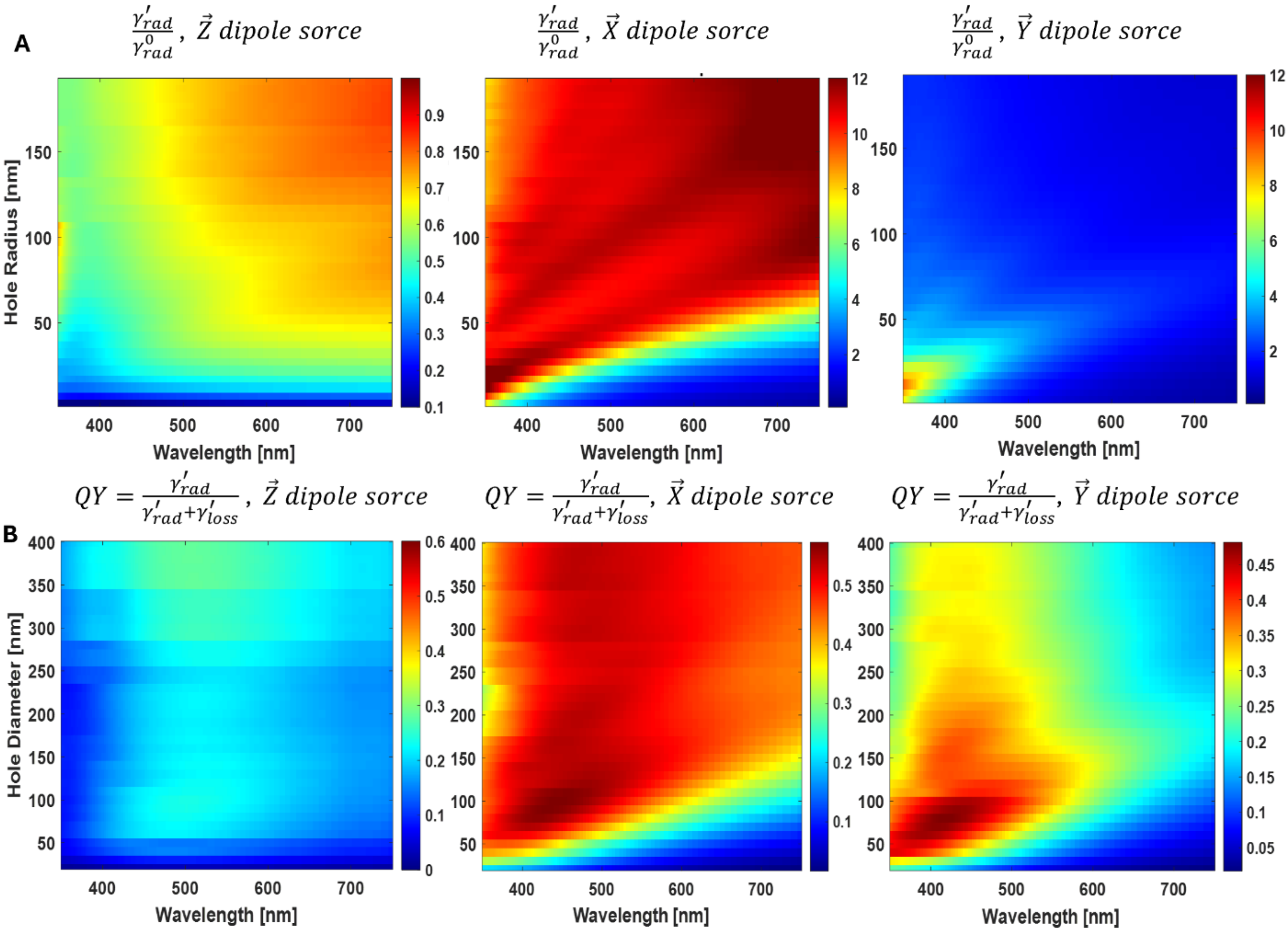

Supporting Figure S27: FDTD simulation of dipole relative quantum yield and radiative rate enhancement for different dipole orientations. (A) The simulated power radiated outside an Al-nanohole with varying diameter by a point dipole located 8 nm from the nanohole side wall on the X axis, relative to the simulated power radiated by the same dipole on a glass substrate. Each row displays 3 different dipole orientations (the electromagnetic radiation propagating mostly in the direction of the plane normal to this direction), showing high enhancement for dipoles oriented in the $\vec{X}$ and $\vec{Y}$ directions. (B) the relative quantum yield (QY) of the dipole source calculated by the power radiated outside the nanohole divided by the total radiated power in the nanohole by the dipole, the loss is the metal absorption (ohmic loss) not considering charge dynamics and Auger recombination in a QD.

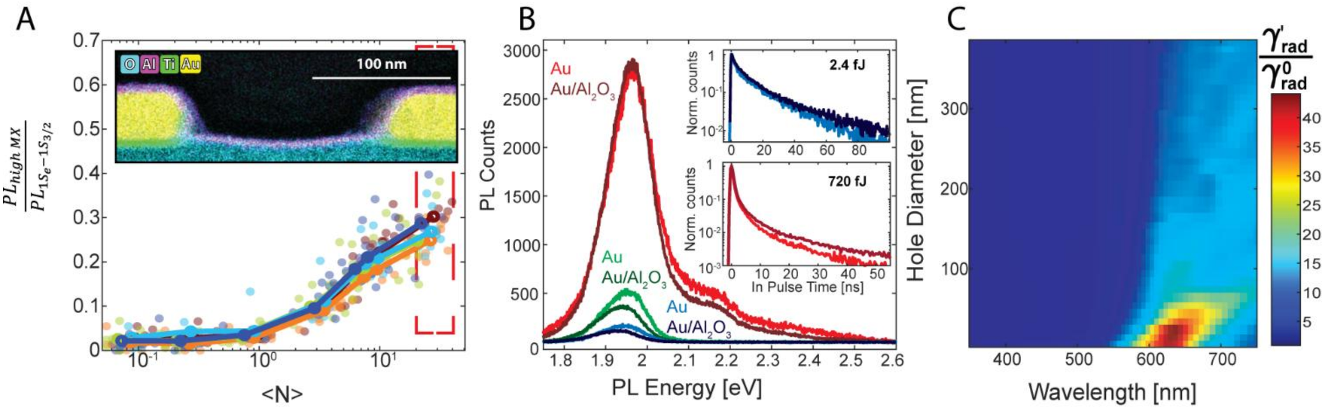

Supporting figure S28: MX PL measurements and FDTD simulation of single QDs in Au-nanoholes with a coating Al₂O₃ layer. (A) Area ratio of $\frac{PL_{high\,MX}}{PL_{1S_e-1S_{3/2}}}$ for 10 single QDs in nanoholes of varying diameter (300 nm- blue, 200 nm-cyan, 150 nm-dark green, 110 nm-light green, 90 nm-orange, 70 nm-dark orange, semi-transparent circles are single measurements, lines are the averaged values). Inset is an Energy-Dispersive X-ray Spectroscopy (EDS) electron microscopy image of a cross section of a 150 nm Au-nanohole with a 4 nm Al_2O_3 coating made by atomic layer deposition. The red rectangle shows the averaged $\frac{PL_{high\,MX}}{PL_{1S_e-1S_{3/2}}}$ values for the nanohole diameters of the gold + Al_2O_3 nanoholes shown in figure 6G in the main text. (B) PL spectra at 2.4 fJ (blue), 7.3 fJ (green), and 720 fJ (red) for a 90 nm nanohole of Au (lighter colors) and Au with the 4 nm AL_2O_3 layer (darker colors). Inset shows the lifetime of the compared QDs at 2 different excitation powers. This comparison reveals that the MX-PL is weaker and the recombination lifetime is extended in the Au/Al_2O_3 nanohole at both low and high excitation powers. While the 4 nm oxide barrier is still not sufficient for blocking the hot charge transfer from the metal to the coupled QD. (C) the FDTD simulated radiation enhancement of a polarization averaged dipole source in Au nanoholes with a 4 nm Al_2O_3 coating, showing a ~40 nm redshift of the plasmon resonance spectra due to the extra layer, now aligning solely with S-band emission energies, further reducing the plasmonic MX enhancement compared to aluminum and gold nanoholes.

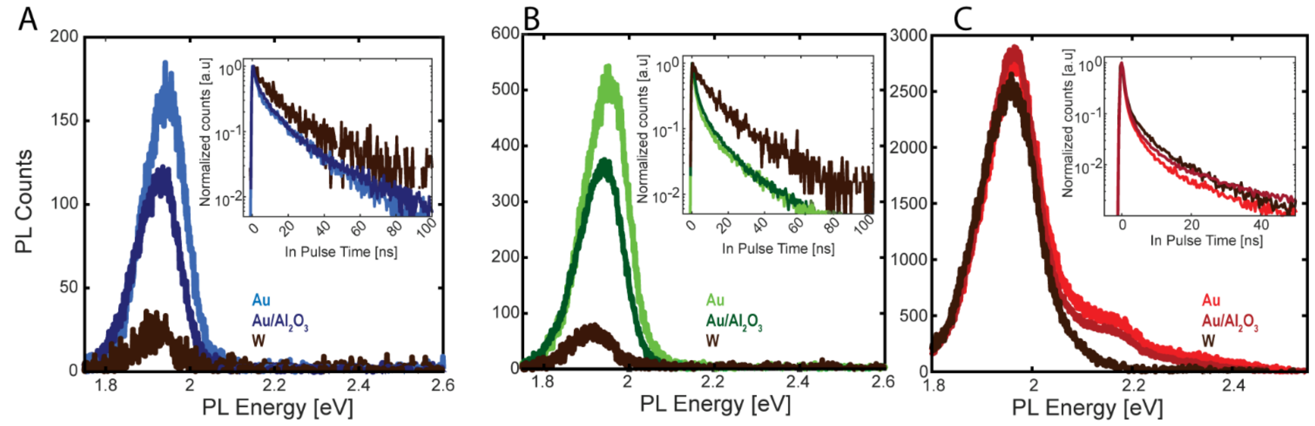

Supporting figure S29: PL spectra and Lifetimes of different metal nanoholes: A-C) PL spectra and lifetime (insets) at 2.4 fJ (A), 7.3 fJ (B), and 720 fJ (C) for the nanoholes from supporting figure S28B compared to a 90nm nanohole in Tungsten (brown lines). The PL of the QD in Tungsten manifests a red shifted PL peak and longer lifetime, indicating no plasmonic MX enhancement and high Ohmic loss, consistent with FDTD simulations in figure 6F in the main text.

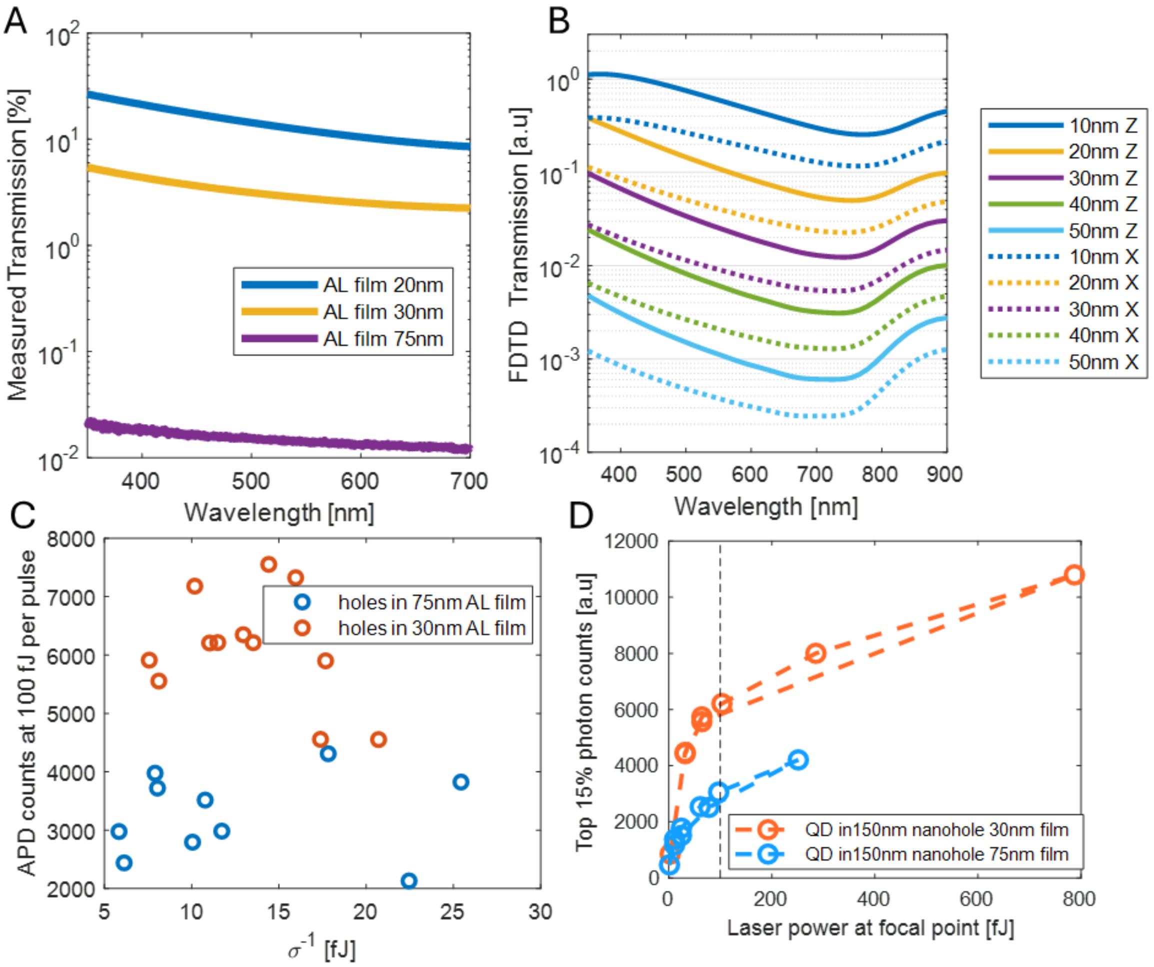

Supporting figure S30: Dependence of nanohole transmission and QD emission on Al film thickness. (A) Measured transmission through nanohole substrates with varying Al film thickness, obtained using a Jasco V-570 UV–vis–NIR spectrophotometer. (B) FDTD simulation of dipole emission transmission for an X/Z-polarized dipole positioned 8 nm above an Al film on glass. The transmitted power is collected in the substrate using a monitor corresponding to a 135° collection angle. For film thicknesses above 30 nm, the transmitted emission is reduced to below ~5% of that of a QD on glass, effectively isolating emission from QDs coupled to nanoholes. (C) PL intensity of single QDs in 150 nm diameter nanoholes for different Al film thicknesses, showing substantially higher emission for thinner films. (D) Power-dependent PL intensity curves for two representative nanoholes from (C), exhibiting similar trends, with consistently higher emission for the 30 nm film. The dashed black line marks the 100-fJ excitation power used for the data shown in (C).

References:

(1) Wenger, J.; Gérard, D.; Dintinger, J.; Mahboub, O.; Bonod, N.; Popov, E.; Ebbesen, T. W.; Rigneault, H. Emission and Excitation Contributions to Enhanced Single Molecule Fluorescence by Gold Nanometric Apertures. *Opt. Express* **2008**, *16* (5), 3008–3020. https://doi.org/10.1364/OE.16.003008.

(2) Park, Y. S.; Malko, A. V.; Vela, J.; Chen, Y.; Ghosh, Y.; García-Santamaría, F.; Hollingsworth, J. A.; Klimov, V. I.; Htoon, H. Near-Unity Quantum Yields of Biexciton Emission from CdSe/CdS Nanocrystals Measured Using Single-Particle Spectroscopy. *Phys. Rev. Lett.* **2011**, *106* (18). https://doi.org/10.1103/PhysRevLett.106.187401.

(3) Scharf, E.; Liran, R.; Levi, A.; Alon, O.; Chefetz, N.; Oron, D.; Banin, U. *Unraveling Size Dependent Bi-and Tri-Exciton Characteristics in CdSe/CdS Core/Shell Quantum Dots via Ensemble Time Gated Heralded Spectroscopy*.

(4) Wang, Z.; Tang, J.; Han, J.; Xia, J.; Ma, T.; Chen, X. W. Bright Nonblinking Photoluminescence with Blinking Lifetime from a Nanocavity-Coupled Quantum Dot. *Nano Lett.* **2024**, *24* (5), 1761–1768. https://doi.org/10.1021/acs.nanolett.3c04661.

(5) Taminiau, T. H.; Stefani, F. D.; Van Hulst, N. F. Single Emitters Coupled to Plasmonic Nano-Antennas: Angular Emission and Collection Efficiency. *New J. Phys.* **2008**, *10*. https://doi.org/10.1088/1367-2630/10/10/105005.

(6) Indukuri, S. R. K. C.; Bar-David, J.; Mazurski, N.; Levy, U. Ultrasmall Mode Volume Hyperbolic Nanocavities for Enhanced Light-Matter Interaction at the Nanoscale. *ACS Nano* **2019**, *13* (10), 11770–11780. https://doi.org/10.1021/acsnano.9b05730.
